# A Stable Isopterin Mimic of a Potent Bacterial Stimulant of T Cells

**DOI:** 10.1002/chem.71494

**Published:** 2026-08-14

**Authors:** Jeffrey Y. W. Mak, Huy N. Hoang, Wael Awad, Xin Yi Lim, Damica D. Laurie, Ryan J. D. Rivero, James McCluskey, Alexandra J. Corbett, Jamie Rossjohn, David P. Fairlie

**Affiliations:** ^1^ Centre for Chemistry and Drug Discovery and ARC Centre of Excellence for Innovations in Peptide and Protein Science, Institute for Molecular Bioscience The University of Queensland Brisbane Queensland Australia; ^2^ Infection and Immunity Program, Department of Biochemistry and Molecular Biology, Biomedicine Discovery Institute Monash University Clayton Victoria Australia; ^3^ Department of Microbiology and Immunology The University of Melbourne, at the Peter Doherty Institute For Infection and Immunity Melbourne Victoria Australia

**Keywords:** conformation analysis, covalent ligands, immunology, natural products, Schiff bases

## Abstract

Mucosal‐associated invariant T cells (MAIT cells) are innate‐like immune cells (T lymphocytes) that protect against bacterial infections. They are potently activated by the microbial metabolite 5‐(2‐oxopropylideneamino)‐d‐ribitylaminouracil (5‐OP‐RU), which bonds covalently to an amine of a specific lysine residue of the antigen‐presenting protein MR1 to form an imine (Schiff base). However, 5‐OP‐RU undergoes rapid degradative intramolecular cyclisation in water, and more robust analogues are needed for therapeutic applications. Here, inspired by the structural similarities between 5‐OP‐RU and 6‐formylpterin, a water‐stable covalent MR1 ligand that does not activate MAIT cells, we describe the design and synthesis of a novel isopterin analogue of this microbial natural product, and show that it is both water‐stable and activates reporter cells expressing the MAIT T cell receptor. Further, we present a protein‐bound crystal structure and molecular dynamics simulations of this stable analogue to show that it mimics the MR1‐bound conformation of 5‐OP‐RU. This study demonstrates the potential of this bicyclic scaffold for stabilising 5‐OP‐RU mimetics, advances our understanding of the molecular requirements for MR1 binding and MAIT cell activation, and informs the design and synthesis of future MAIT cell antigens as potential immunotherapeutics.

## Graphical abstract

A microbial metabolite covalently binds immunological protein MR1 to potently activate specific lymphocytes called MAIT cells, but it is too unstable for biological applications. Here, using synthesis, assays, protein crystallography, and computer modelling, we report a water‐stable analogue that activates reporter cells expressing the MAIT T cell receptor and mimics the protein‐bound conformation of the natural product, forming a basis for designing new immunotherapeutics.

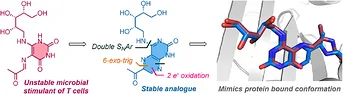

## 1 Introduction

Human mucosal‐associated invariant T cells (MAIT cells) [1] are unusual T cells found in relatively high abundance in blood, liver, lungs and gut [2]. They have attracted considerable recent attention due to their capacity to kill myeloma cells in vitro [3], protect against bacterial [4, 5, 6, 7] and viral [8, 9, 10] infections, autoimmune encephalomyelitis [11], cancer [12], and promote wound healing [13, 14], suggesting their potential as new therapeutic targets [15, 16, 17]. MAIT cells are unlike most other T cells in being activated by small heterocyclic ligands (antigens) rather than peptides or glycolipids [18]. The most potent activator is the Schiff base‐containing microbial natural product 5‐(2‐oxopropylideneamino)‐d‐ribitylaminouracil (5‐OP‐RU, 1, Figures 1 and 2), which features a uracil ring joined to a ribityl chain and an iminocarbonyl warhead [19, 20, 21].

**FIGURE 1:**
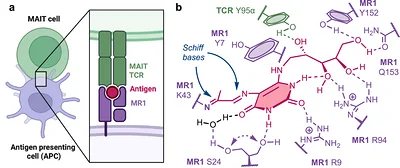
(a) MAIT cells are activated by formation of an MR1‐antigen‐TCR complex at the APC‐MAIT cell‐cell interface. (b) Interactions of 5‐OP‐RU (**1**, pink) with MR1 (purple) and a MAIT TCR (green) protein as revealed in a crystal structure [19]. Adapted with colour and font adjustments from ref. [22], licensed under the Creative Commons Attribution 4.0 International (CC BY 4.0) License.

To activate MAIT cells, 5‐OP‐RU (**1**) initially binds to the immunological protein Major Histocompatibility Complex class I‐related protein 1 (MR1) inside an antigen‐presenting cell [23, 24]. The MR1‐antigen complex then translocates to the cell surface for interaction with the MAIT T cell receptor (MAIT TCR) at the cell‐cell interface of an antigen‐presenting cell (APC) and MAIT cells to trigger MAIT cell activation (Figure 1a). A crystal structure [19] of 5‐OP‐RU (**1**), simultaneously bound to MR1 and TCR proteins, showed an extensive H‐bond network, as well as a covalent imine bond (i.e., a second Schiff base) between its ketone and MR1 Lys43 that confers potency [20] and promotes MR1 folding [24] (Figure 1b). The 5‐OP‐RU antigen is effectively sandwiched between the MR1 and TCR proteins, thereby acting as a bridge between two different cell types (APC and MAIT cells, Figure 1).

Despite the exquisite biological properties of metabolite **1** (Figure 2), its usefulness as a tool and therapeutic lead is limited by instability, as it undergoes rapid cyclisation in water (t_1/2_ 88 min, pH 7.4, 37 °C) to the much less active lumazine **2** (Figure 2) [21]. Stable analogues have been developed, but none has matched the MAIT cell activation potency [21] of 5‐OP‐RU (**1**, EC_50_ 2 pM). For example, we previously replaced the sp^2^‐hybridised exocyclic nitrogen atoms of 5‐OP‐RU (**1**) with carbons (e.g. JYM72 (**4**)) [21, 22, 25], while Inuki and co‐workers replaced the iminocarbonyl warhead with a more electrophilic saturated ketone (**5**) (Figure 2) [26]. However, those modifications increased ribityl chain or warhead flexibility, and/or altered ligand binding conformation, reducing MAIT cell activation potency (EC_50_ 2–50 nM) [25, 26]. Prodrug forms of the biosynthetic precursor of 5‐OP‐RU have been reported [27, 28], but they do not address the instability of 5‐OP‐RU (**1**). New strategies to more rigidly mimic the protein‐bound conformation of 5‐OP‐RU with water‐stable analogues are required.

**FIGURE 2:**
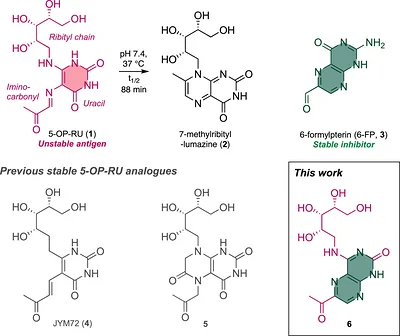
5‐OP‐RU (**1**) undergoes rapid intramolecular cyclisation and inactivation to **2**, which, together with stable inhibitor 6‐formylpterin (6‐FP, **3**), inspired the creation of stable analogue **6**. JYM72 (**4**) [21] and ribityllumazine **5** [26] are known analogues of **1** shown for comparison.

## 2 Results and Discussion

### 2.1 Design of a More Stable Antigen

An alternative strategy to stabilise the labile imine of 5‐OP‐RU (**1**) is to incorporate it into a ring, while retaining the exocyclic nitrogens. This is expected to rigidly project both the warhead and the ribityl chain for interactions with MR1 and the MAIT TCR, while preventing C═N bond hydrolysis and intramolecular cyclisation, giving a stable scaffold that mimics the conformational range of 5‐OP‐RU (**1**). As proof‐of‐principle, we designed compound **6** (Figure 2), inspired by its structural analogy with 5‐OP‐RU (**1**) and also 6‐formylpterin (6‐FP, **3**), a water‐stable bicyclic photodegradation product of folic acid that binds covalently to MR1 but does not activate MAIT cells as it has no ribityl chain [18, 29]. Accordingly, we hypothesised that **6**, an isopterin with a ribityl chain and similar decoration to 5‐OP‐RU (**1**), might exemplify a new strategy for mimicking 5‐OP‐RU. A ketone warhead was selected for its anticipated superior metabolic stability.

### 2.2 Chemical Synthesis

There is no synthetic precedent for a comparably substituted isopterin, but we envisaged that compound **6** could be formed via successive S_N_Ar reactions of appropriate *N*‐nucleophiles to 2,4,6‐trichloronitropyrimidine (**14**, Scheme 2), followed by a tandem reduction/regioselective intramolecular cyclisation.To form the first *N*‐nucleophile, d‐ribose was first reductively ring‐opened with benzylamine to give **7**, before treating with ethyl formate in ethanol. These mild conditions solubilised the highly polar **7** and effected a chemoselective protection of its amine with a base‐labile formyl group (to **8**, Scheme 1a). With its amine now fully protected, isopropylidene protection of the tetraols (**9**), formyl deprotection with lithium hydroxide (to give **10**), and then hydrogenolysis gave amine **11**. This five‐step sequence converted the water‐soluble sugar into an organic‐soluble derivative on gram scale via only one purification. For the second *N*‐nucleophile, we prepared amine **13** via a lithium aluminium hydride reduction of methacrylaldehyde‐derived cyanohydrin **12** (Scheme 1b).

**SCHEME 1:**
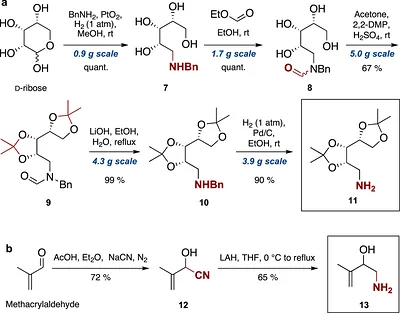
(a) Synthesis of *N*‐nucleophile **11** from d‐ribose; (b) synthesis of *N*‐nucleophile **13** from methacrylaldehyde.

With both amines in hand, one‐pot treatment of trichloronitropyrimidine (**14**) with **11** at −78 °C, and then with **13** at 0 °C, gave the chromatographically stable double S_N_Ar adduct **15** with excellent regioselectivity in 45% yield (Scheme 2). A final S_N_Ar reaction with aqueous lithium hydroxide at 50 °C yielded advanced intermediate **16** (95%).

**SCHEME 2:**
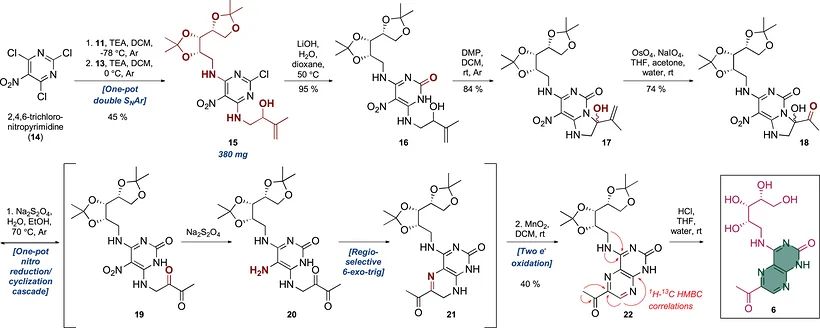
Synthesis of water‐stable isopterin **6** via a one‐pot double S_N_Ar backbone assembly, followed by an oxidation/reduction sequence to form an acetylpyrazine ring.

Next, we performed an oxidation/reduction sequence to form the acetylpyrazine ring, the order of which was important (Scheme 2). The congested and densely functionalised **16** was oxidised with Dess‐Martin periodinane to the corresponding ketone (trapped as diastereoisomeric hemiaminals **17**, 84%). Oxidative cleavage to ketone **18** (74%), followed by chemoselective nitro reduction, led to concomitant and spontaneous regioselective 6‐*exo*‐trig cyclocondensation (likely through **19**–**21**). After workup, the crude dihydropyrazine product was treated with activated manganese dioxide in refluxing toluene to effect a two‐electron oxidation and yield acetylpyrazine **22**. This multi‐process sequence converted **18** to **22** in 40% yield. The structure of **22** was established by ^1^H‐^13^C HMBC NMR spectroscopy and DFT calculations (Figure S1).

Final deprotection in aqueous hydrochloric acid gave acetylisopterin **6**. Density Functional Theory (DFT) calculations suggested that tautomer A was the most stable tautomer by 7.4 kcal/mol, and best matched the NMR data (Figure 3). Consistent with its stability in aqueous acid (Scheme 2), **6** was stable in phosphate‐buffered saline (pH 7.4, 37 °C) over 24 h, unlike 5‐OP‐RU (**1**) [21], which underwent intramolecular cyclization under the same conditions (t_1/2_ 88 min) (Figure 4a).

**FIGURE 3:**
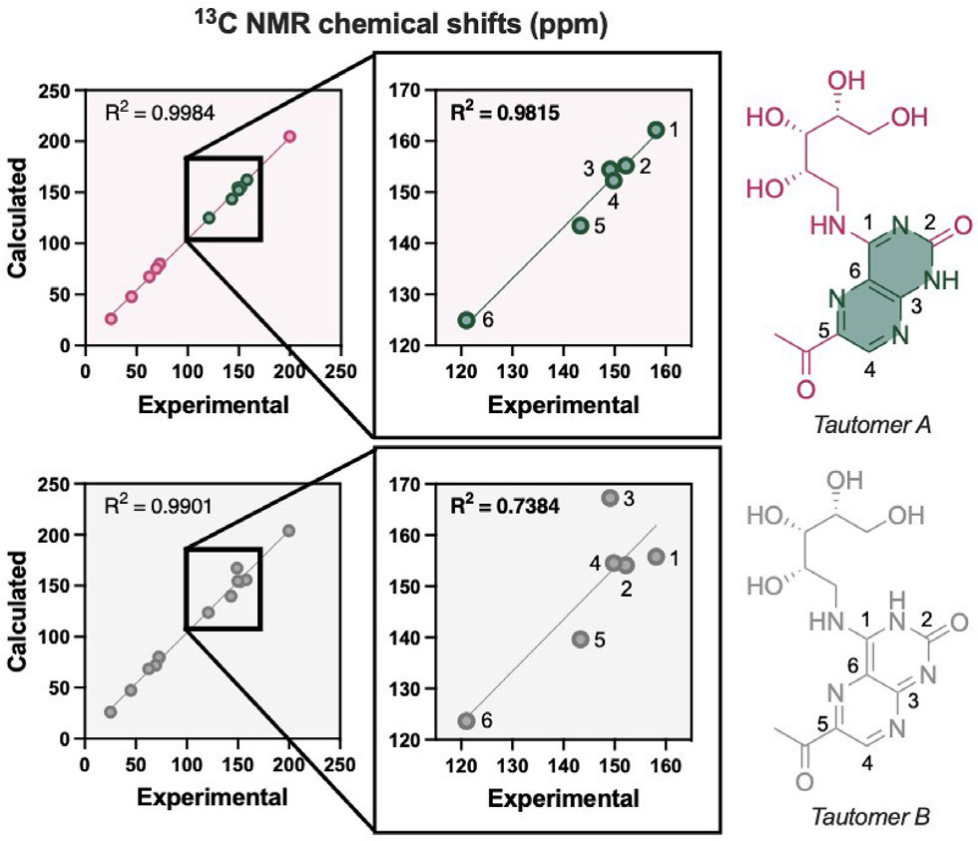
DFT‐calculated ^13^C NMR chemical shifts (under PCM water solvation conditions) for two tautomers of compound **6** plotted against experimental ^13^C NMR chemical shifts (measured in 10% D_2_O/H_2_O). Chemical shifts of ring carbons shown in insets.

**FIGURE 4:**
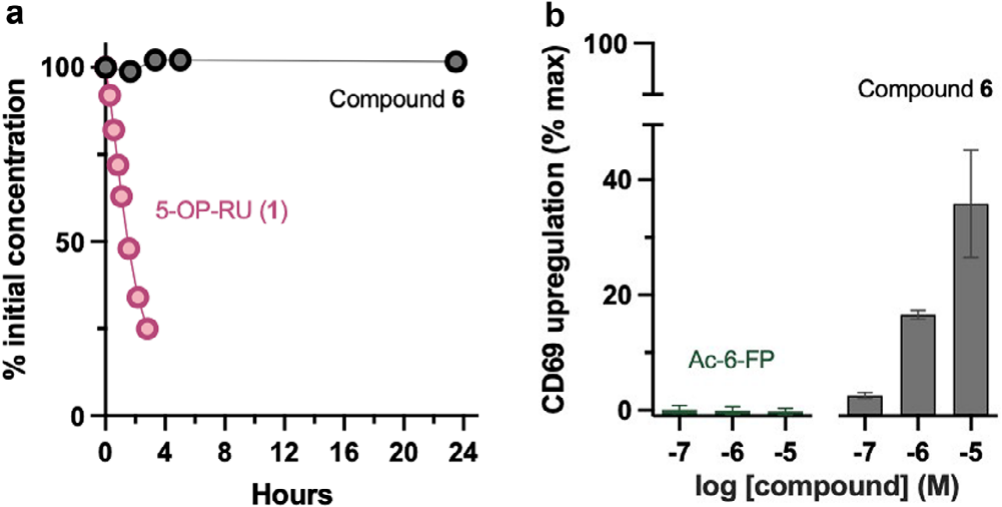
(a) Stability of compound **6** versus 5‐OP‐RU (**1**) [21] in phosphate buffered saline (pH 7.4, 37 °C). (b) Activation of Jurkat.MAIT cells by compound **6**, compared to control Ac‐6‐FP, the *N*‐acetylated analogue of 6‐FP (**3**), as measured by upregulation of protein surface marker CD69. Response to 100 nM 5‐OP‐RU (**1**) defined as 100%. Bars represent means ± SEM (*n* = 3).

### 2.3 Cell Surface Upregulation of MR1 and MAIT Reporter Cell Activation

We used an established assay [19] to test whether **6** could bind MR1 and enable it to translocate to the cell surface. Compound **6** was incubated with C1R.MR1 cells, and MR1 surface expression was detected with an MR1 antibody. Surprisingly, no increase in cell surface MR1 was detected (up to 100 µM, Figure S2). Next, we used another established but more sensitive assay [18] to test whether **6** could bind to MR1 and then activate MAIT TCR‐expressing reporter cells. Compound **6** was co‐incubated with C1R.MR1 cells and Jurkat cells transfected with the A‐F7 MAIT TCR (Jurkat.MAIT cells [18]). The upregulated expression of a protein marker (CD69) on the surface of Jurkat.MAIT cells was then used as a quantitative measure of T cell activation [19]. The assay showed that **6** bound MR1 and activated Jurkat.MAIT cells in a dose‐dependent manner at 1–10 µM (Figure 4b), albeit with lower potency [22] than 5‐OP‐RU (**1**, EC_50_ 56 pM). While the EC_50_ value for **6** could not be determined, it is expected to be greater than 10 µM. By comparison, other stable antigens with ketone warheads (**4** and **5**, Figure 2) activate MAIT reporter cells with EC_50_ 1.6–26 nM [21, 22, 26], while a metabolite of the drug diclofenac [30] and lumazines from *Mycobacterium smegmatis* [31, 32] non‐covalently bind MR1 and activate MAIT cell clones only at 10–200 µM.

### 2.4 Crystal Structure of MAIT TCR in Complex With MR1 and Compound 6

To determine the binding mode of **6** and the effectiveness of its mimicry of 5‐OP‐RU (**1**), a ligand exchange approach was used to determine a high‐resolution (∼2 Å) crystal structure of MAIT A‐F7 TCR in complex with MR1 and **6** (Figure 5, Table S1). In contrast to bicyclic **5** [26], compound **6** adopted a very similar protein‐bound structure to 5‐OP‐RU (including an intramolecular ribityl…heterocycle H‐bond [33]). Overall, the structure recapitulated the hydrogen bonding interactions between 5‐OP‐RU and the two proteins, as well as Schiff base formation between the ketone of **6** and MR1 Lys43, albeit with slight movement of the Lys43 side chain.

**FIGURE 5:**
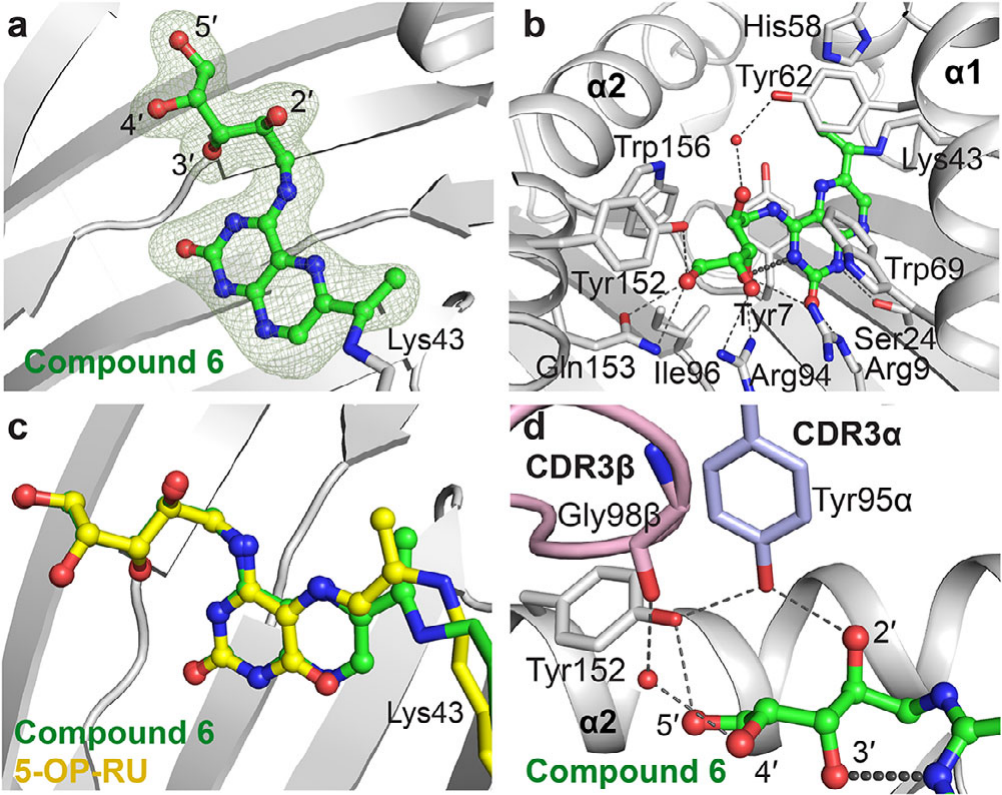
Crystal structures of MR1 and MAIT TCR proteins bound to **6** (PDB 9Z4R) and 5‐OP‐RU (**1**, PDB 4NQC) [19] show conserved ligand‐protein interactions and binding modes. (a) Omit map contoured at 3σ (Fo–Fc map; green mesh) of compound **6** after simulated annealing refinements in the Phenix crystallography package. (b) Conserved interactions between compound **6** and residues in the MR1 pocket. (c) Superposition of **6** (green) and **1** (yellow) within the MR1 binding cleft, showing Schiff bases between MR1 Lys43 and both ligands. (d) Conserved H‐bonding interactions between compound **6** (green) and Tyr152 (grey) of MR1, Tyr95a (lilac) and Gly98b (pink) of the complementarity determining regions (CDR3α and CDR3β) of the A‐F7 MAIT TCR, as well as between the 3′‐OH and a ring nitrogen (black dash).

### 2.5 Computer Modelling

Computer modelling was used to more closely examine the protein binding of **6**. DFT calculations on ribityl‐truncated models of **4**, **5** and **6** (Figures S3 and S4) indicated similar partial charges of +0.544, +0.605, and +0.568, respectively, on their ketone carbon atoms. Despite this, while the Gibbs free energy of imine formation with methylamine (a model of MR1 Lys43) was 0.98 kcal/mol and 0.65 kcal/mol more favourable for **6** over **4** and **5**, respectively, the initial nucleophilic attack was 1.78 kcal/mol and 5.28 kcal/mol higher in energy, possibly due to the steric bulk of the pyrazine ring.

Next, 200 ns molecular dynamics simulations were performed for 5‐OP‐RU (**1**) and analogues **4**, **5**, and **6** in complexes with MR1 and the MAIT TCR (Figure S5). Although modifications to the uracil ring can significantly interfere with binding of ligands to MR1 [22], **6** was found to bind stably to the MR1 binding cleft throughout, as quantified by the distance between MR1‐Tyr7 and the ribityl‐adjoining ring [22], with or without a covalent bond to Lys43, with or without the MAIT TCR (Figure S5). This supported the notion [22] that noncovalent interactions alone between MR1 and **6** could sequester the ligand for subsequent covalent linkage formation.

For the simulations with covalent complexes, we compared the projections of the ribityl chains for interaction with the MAIT TCR, first quantifying the torsion of the two bonds (designated α and β) between the ribityl chain and the cyclic scaffolds (Figure 6a,b). Compound **6** best mimicked the conformation of 5‐OP‐RU, with **5** exhibiting a similar but more flexible torsional profile. Compound **4** had the least rigid chain, as its α bond uniquely had no double bond character.

**FIGURE 6:**
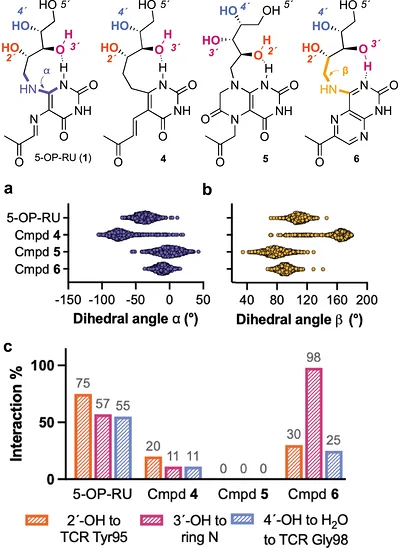
Molecular dynamics simulations (200 ns) of MR1 covalently bound to antigens 5‐OP‐RU (PDB 4NQC), **4** (PDB 6PUK), **5** (PDB 8Y6X), or **6** (PDB 9Z4R) with the MAIT TCR. (a, b) Torsional profiles of the two bonds (labelled α and β) between the ribityl chain and the cyclic scaffolds. (c) Percentage of time in which key H‐bond interactions were observed from ribityl 2´‐OH, 3´‐OH, and 4´‐OH.

Examining the ligand‐protein interactions (Figure 6c), the 3′‐OH of 5‐OP‐RU maintained its intramolecular H‐bond with uracil 57% of the time. This rigidified the projections of 2′‐OH and 4′‐OH for H‐bonding with TCR Tyr95 (75%) and Gly98 (water‐bridged, 55%), respectively. Amongst the synthetic antigens, **6** was most similar to 5‐OP‐RU with respect to these parameters, followed by the more flexible **4**. The interaction profile of **5** was distinct from 5‐OP‐RU, as its uracil NH was H‐bonded to the 2′‐OH instead of the 3′‐OH, in both the solid state and simulations.

Together, the MAIT reporter cell activation assay, crystallographic, and computational data indicate that non‐covalent interactions can template Schiff base formation between MR1 Lys43 and the electrophilic ketone of **6**. This ketone was less reactive to Lys43 than the ketones of **4** and **5**, potentially leading to its reduced ability to activate reporter cells expressing the MAIT TCR (although the degree of ligand cell penetration may also contribute to potency). However, once covalently bound, **6** engaged MR1 and the MAIT TCR in a manner that closely mimicked 5‐OP‐RU (**1**).

## 3 Conclusion

By targeting MAIT cells, the compound 5‐OP‐RU (**1**) has shown potential as a vaccine adjuvant (antibacterial [4, 5, 6] and antiviral [8, 34, 35]), for overcoming antibiotic resistance [36, 37], for reduction of tumour metastases [12], and in tissue repair [14, 38]. However, there are few water‐stable analogues of 5‐OP‐RU [21, 22, 26], due to the considerable challenge of designing a warhead‐containing ligand that can effectively and simultaneously bind to two target proteins to link two separate cell types. Here, we have demonstrated through design, synthesis, cellular assays, protein crystallography, and computational modelling that incorporation of the labile imine bond of potent MAIT cell stimulant 5‐OP‐RU (**1**) into an aromatic ring afforded a water‐stable analogue that activated the MAIT reporter cells at low micromolar concentrations. Compound **6** recapitulated the protein binding profile of 5‐OP‐RU (**1**), rigidly projecting its covalent warhead for reaction with MR1 Lys43, and once covalently bound to MR1, orientating its ribityl chain for interaction with the MAIT TCR. This study demonstrates the potential of this bicyclic scaffold for developing mimics of 5‐OP‐RU, and provides new insights into electrophilic warhead electronics that can potentially inform the design and synthesis of MAIT cell antigens for development into immunotherapeutics.

## Conflicts of Interest

JYWM, JMc, AJC, JR, and DPF are among inventors on patents describing MR1 ligands and tetramers, including MAIT cell activators.

## Supporting information



Supplementary Figures, Supplementary Tables, experimental methods, and spectral data for all new products are within the Supporting Information. The crystal structure of compound **6** bound to the MR1 and MAIT TCR proteins is deposited in the Protein Data Bank (PDB 9Z4R; https://doi.org/10.2210/pdb9Z4R/pdb). The authors have cited additional references within the Supporting Information [39, 40, 41, 42, 43, 44, 45, 46, 47, 48, 49, 50].
**Supporting File 1**: chem71494‐sup‐0001‐SupMat.pdf

## Data Availability

The data that support the findings of this study are available in the supplementary material of this article.
