# Supplementary material for: A Stable Isopterin Mimic of a Potent Bacterial Stimulant of T Cells
Source: Chemistry. 2026 Aug 14;32(41):e71494. doi: 10.1002/chem.71494 (PMC13729931; doi:10.1002/chem.71494)
Supplement: Supplementary file 1 [file CHEM-32-e71494-s001.pdf]

Supporting information

A Stable Isopterin Mimic of a Potent Bacterial Stimulant of T cells

Jeffrey Y. W. Mak,*[a] Huy N. Hoang,[a] Wael Awad,[b] Xin Yi Lim,[c] Damica D. Laurie,[a] Ryan J. D. Rivero,[a] James McCluskey,[c] Alexandra J. Corbett,[c] Jamie Rossjohn,[b] David P. Fairlie*[a]

[a] Centre for Chemistry and Drug Discovery and ARC Centre of Excellence for Innovations in Peptide and Protein Science, Institute for Molecular Bioscience, The University of Queensland, 4072, Brisbane, Queensland, Australia.

[b] Infection and Immunity Program, Department of Biochemistry and Molecular Biology, Biomedicine Discovery Institute, Monash University, Clayton, Victoria 3168, Australia.

[c] Department of Microbiology and Immunology, The University of Melbourne, at the Peter Doherty Institute for Infection and Immunity, Melbourne, Victoria 3000, Australia.

Table of Contents

Figure S1: DFT calculated ^{13}C NMR chemical shifts (under PCM chloroform solvation conditions) for two tautomers of compound **22** versus experimental data (obtained in $CDCl_3$). Chemical shifts of ring carbons shown in inset. 2

Figure S2: Dose-dependent upregulation of MR1 expression on the surface of C1R.MR1 cells after 16 h exposure to compound **6** or acetyl 6-formylpterin (Ac-6-FP). Upregulation by 0.1 mM Ac-6-FP defined as 100 %. Each data point represents mean±SEM (*n*=3). 3

Figure S3: Heavy atom partial charges of truncated models of ligands **4**,[1] **5** and **6**...4

Figure S4: Calculated Gibbs free energies for the gas phase reaction between methylamine and truncated models of compounds **4**, **5**, and **6** at 298 K (calculated with Schrodinger 2023-3 at the B3LYP/6-311+ G(2d,2p) level of theory). 5

Figure S5: Ligand **6** bound stably within MR1, as quantified by the distance between the centres of the ribityl-adjoining ring and the phenol of MR1 Tyr7, with or without a covalent bond between ligand and MR1 Lys43, with or without MAIT TCR. 6

Table S1: Crystallography data collection and refine statistics 7

Computer calculations methods...8

Crystallography methods..10

Biological assay methods...11

General chemical methods...12

Synthetic procedures..13

NMR Spectra..26

References...42

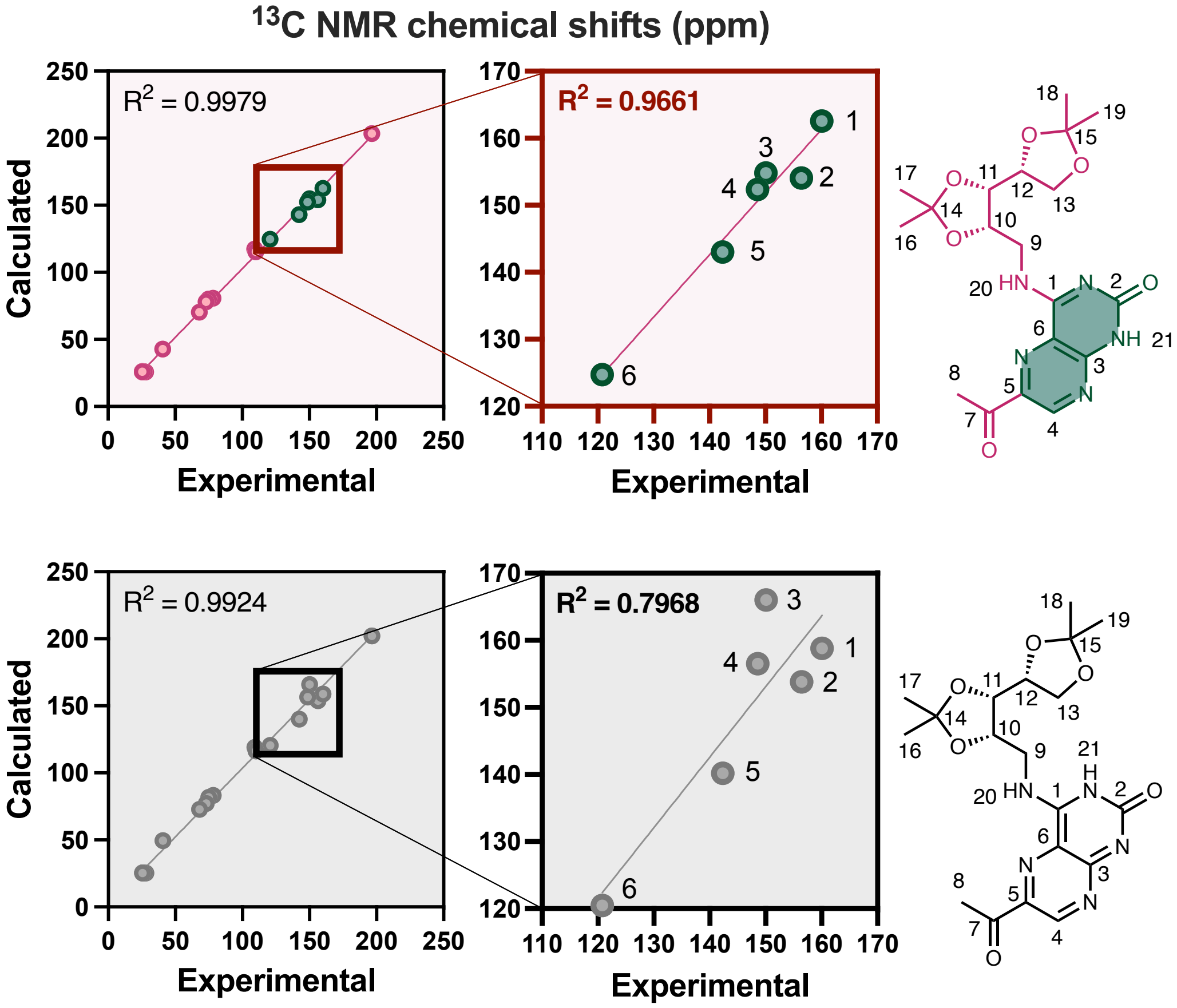

Figure S1: DFT calculated ^{13}C NMR chemical shifts (under PCM chloroform solvation conditions) for two tautomers of compound **22** versus experimental data (obtained in $CDCl_3$). Chemical shifts of ring carbons shown in inset.

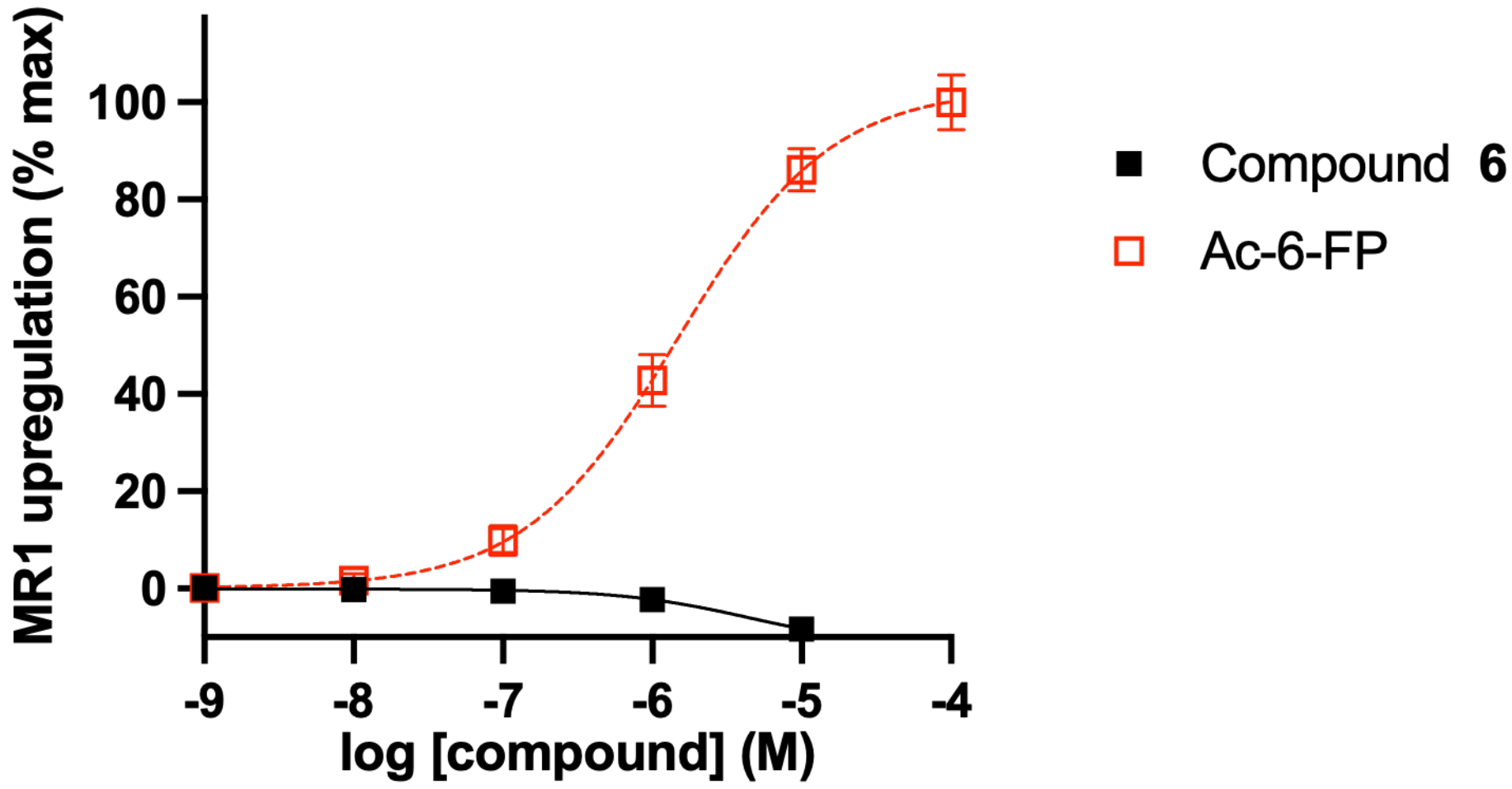

Figure S2: Dose-dependent upregulation of MR1 expression on the surface of C1R.MR1 cells after 16 h exposure to compound **6** or acetyl 6-formylpterin (Ac-6-FP). Upregulation by 0.1 μM Ac-6-FP defined as 100 %. Each data point represents mean±SEM (*n*=3).

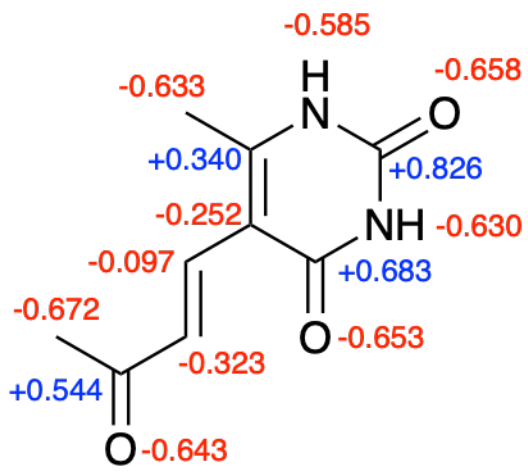

Truncated **4**

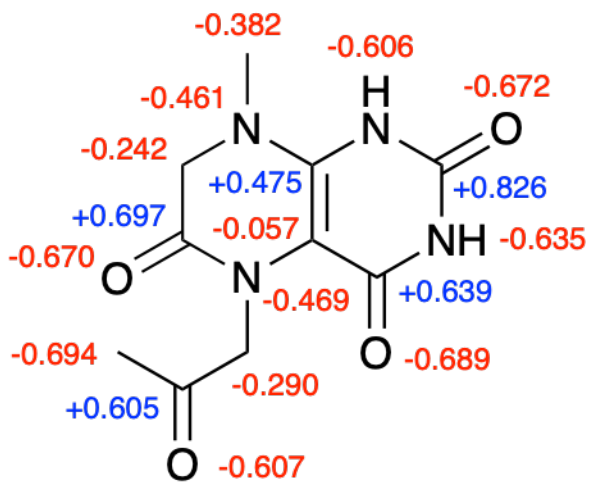

Truncated **5**
(rotamer from ligand-protein
crystal structure PDB 8Y6X)

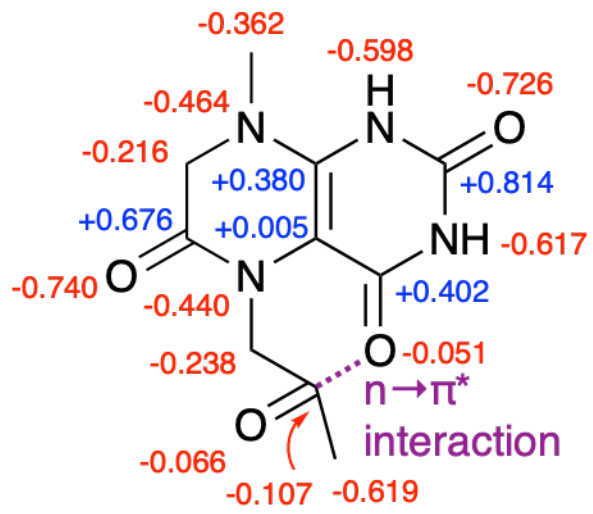

Truncated **5**
(lowest energy
conformation *in vacuo*)

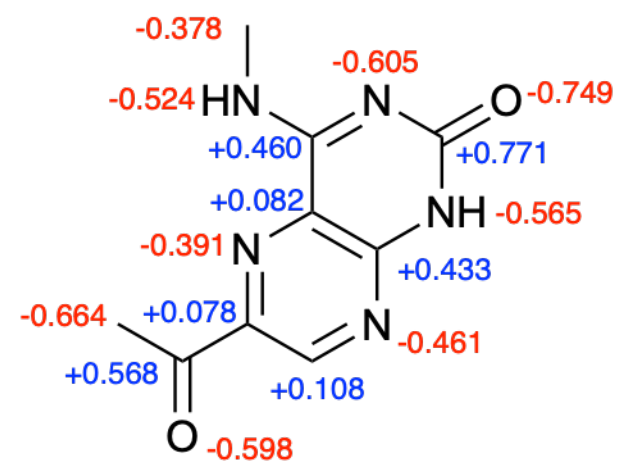

Truncated **6**

Figure S3: Heavy atom partial charges of truncated models of ligands **4**,[1] **5** and **6**.

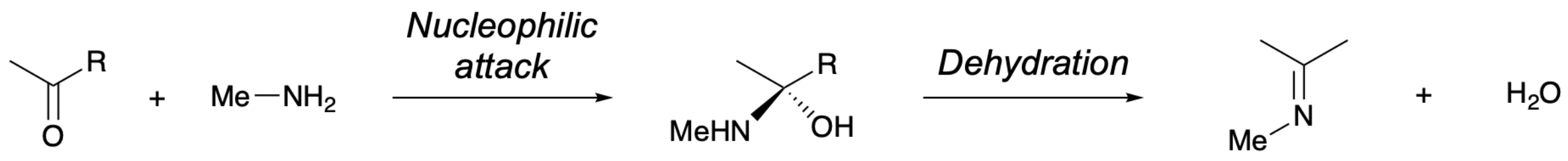

Ligand	Nucleophilic attack	Dehydration	Overall
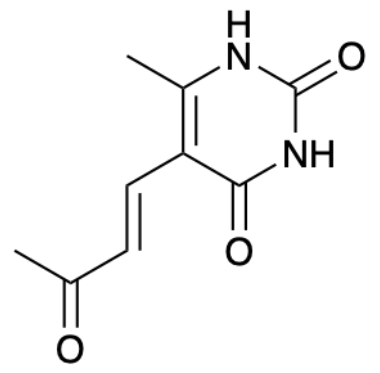 Truncated **4**	$\Delta G^{\circ}_{vac,298}$ 8.78 kcal/mol	$\Delta G^{\circ}_{vac,298}$ -1.55 kcal/mol	$\Delta G^{\circ}_{vac,298}$ 7.23 kcal/mol
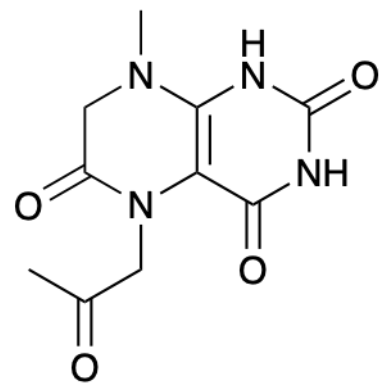 Truncated **5**	$\Delta G^{\circ}_{vac,298}$ 5.30 kcal/mol	$\Delta G^{\circ}_{vac,298}$ 0.60 kcal/mol	$\Delta G^{\circ}_{vac,298}$ 6.90 kcal/mol
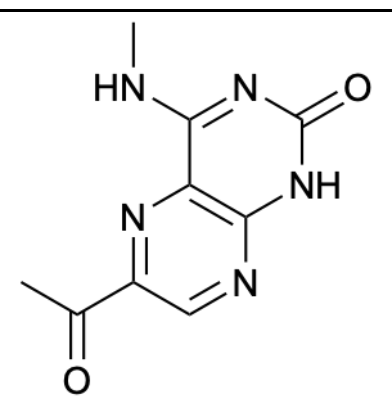 Truncated **6**	$\Delta G^{\circ}_{vac,298}$ 10.58 kcal/mol	$\Delta G^{\circ}_{vac,298}$ -4.33 kcal/mol	$\Delta G^{\circ}_{vac,298}$ 6.25 kcal/mol

Figure S4: Calculated Gibbs free energies for the gas phase reaction between methylamine and truncated models of compounds **4**, **5**, and **6** at 298 K (calculated with Schrodinger 2023-3 at the B3LYP/6-311+ G(2d,2p) level of theory).

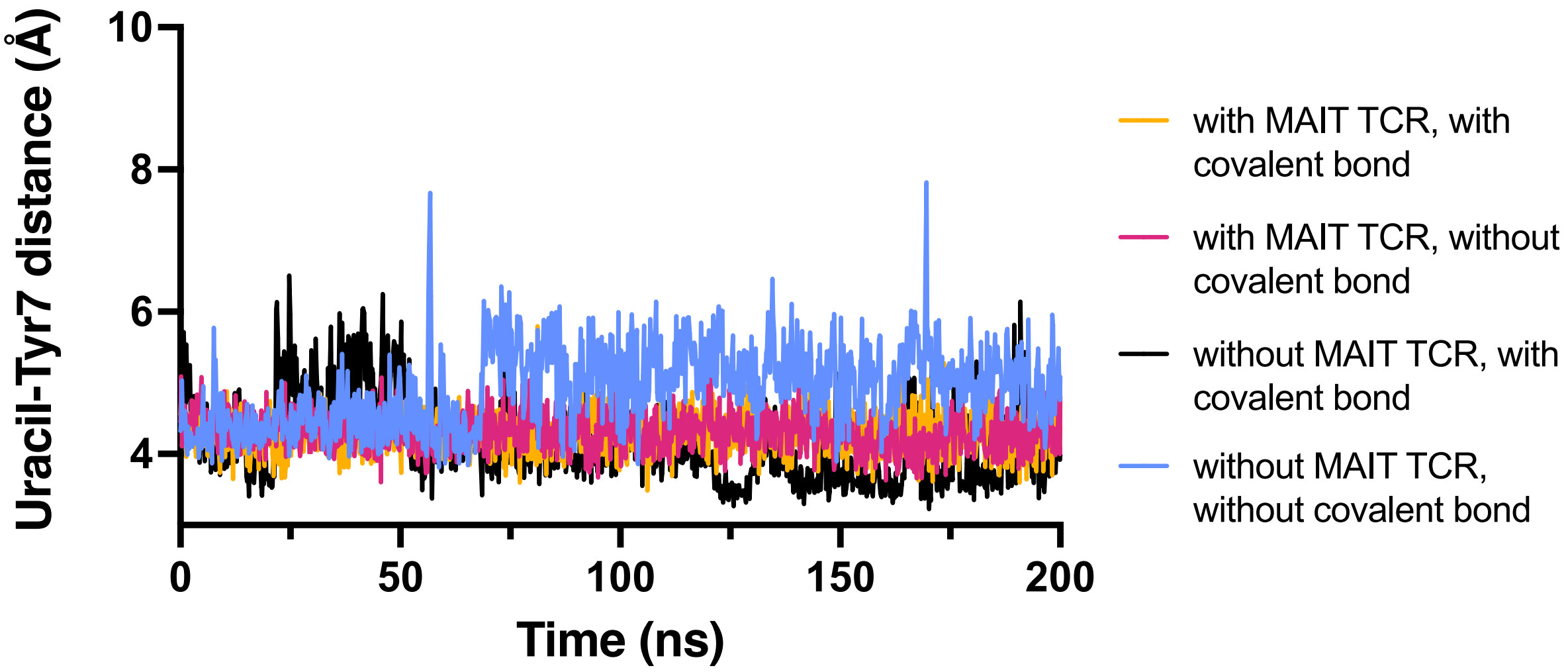

Figure S5: Ligand **6** bound stably within MR1, as quantified by the distance between the centres of the ribityl-adjoining ring and the phenol of MR1 Tyr7, with or without a covalent bond between ligand and MR1 Lys43, with or without MAIT TCR.

Table S1: Crystallography data collection and refine statistics

	TCR-MR1-compound 6*
**Wavelength**	0.9537
**Resolution range**	39.8 – 2.08 (2.1 – 2.08)
**Space group**	C 1 2 1
**Unit cell**	216.433 69.897 142.599 90 104.21 90
**Total reflections**	600982 (19213)
**Unique reflections**	242078 (8059)
**Multiplicity**	2.5 (2.4)
**Completeness (%)**	99.32 (92.12)
**Mean I/sigma(I)**	8.88 (1.05)
**Wilson B-factor**	32.04
**R-merge**	0.08281 (0.8054)
**R-pim**	0.06507 (0.6316)
**CC1/2**	0.996 (0.476)
**CC***	0.999 (0.803)
**R-work**	0.1810 (0.3015)
**R-free**	0.2147 (0.3017)
**Number of non-hydrogen atoms**	14940
**macromolecules**	13094
**ligands**	92
**solvent**	1754
**Protein residues**	1610
**RMS (bonds)**	0.003
**RMS (angles)**	0.63
**Ramachandran favored (%)**	98.49
**Ramachandran allowed (%)**	1.51
**Ramachandran outliers (%)**	0.00
**Rotamer outliers (%)**	2.60
**Average B-factor**	39.58
**macromolecules**	38.90
**ligands**	36.52
**solvent**	44.87

Statistics for the highest-resolution shell are shown in parentheses.

Computer calculations methods

Tautomer energies of ligands were calculated using the Schrodinger 2022-3 software suite. Ligands were drawn using the 2D Sketcher, submitted to the LigPrep module of for preparation using the OPLS3e force field, before energy calculations using the Single Point Energy Optimization function in the Jaguar module (v 11.7) at the B3LYP-D3 6-31G** level of theory under PCM water solvation conditions.

Atomic partial charges of compounds were obtained using *ab initio* density functional theory (DFT) calculations at the B3LYP/6-311+ G(2d,2p) level of theory using the NBO 7.0 program within Gaussian 16 via the graphical interface GaussView 9.

NMR chemical shifts were calculated using the using Schrodinger 2022-3 software suite. Ligands were drawn using the 2D Sketcher, submitted to the LigPrep module of for preparation using the OPLS3e force field, before performing the Optimization function in Jaguar module at the Accurate level and B3LYP-D3 6-31G** level of theory, under PCM solvation conditions, to obtain the 2D averaged chemical shifts.

Gas phase reaction Gibbs free energies were calculated using Jaguar module at the B3LYP/6-311+ G(2d,2p) level of theory in the Schrodinger 2023-3 software package. All compounds were first constructed as 2D structures with ChemDraw, converted to 3D SDF (Structure Data File) format, submitted to the LigPrep module of the Schrödinger package for preparation using the OPLS3e force field, before being submitted for reaction energy calculations using the Jaguar Reaction Energies function at the “Accurate” level under gas phase solvation conditions. The “pre-optimise geometry” option was selected.

All molecular dynamics simulations were performed using Maestro in the Schrodinger simulation package 2023-3. The force field topologies of the small molecule ligands were derived from the OPSL3e parameter set. The crystal structures of the ligand-protein complexes (with or without the TCR removed) were prepared with the Prepare function in Desmond module using the OPLS3e force field, before placing in a pre-equilibrated truncated 10 Angstrom sided square box filled with SPC water molecules. To generate the non-covalent ligand-protein complexes, the covalent imine bond between ligand and MR1 Lys43 was removed using the Builder module in Maestro to regenerate the carbonyl of the ligand and the amine of MR1 Lys43, prior to employing the Prepare function. The simulations were carried out at 300 K. After a steepest descent minimization step, the protein-ligand complexes were each subjected to a 100 ps simulation with position restraints on the complex to relax the solvent. The systems were subsequently equilibrated for 1 ns without restraints. The simulations were performed at constant temperature (300 K) and pressure (1 atm). This was achieved using a Berendsen thermostat with a coupling time of 0.1 ps and a Berendsen barostat

with a coupling time of 0.5 ps. Non-bonded interactions were calculated using a twin-range cut-off. Interactions within the short-range cut-off of 0.8 nm were updated every time-step. Interactions within the longer-range cut-off of 1.4 nm were updated every 5 time-steps together with the pairlist. All bonds were constrained using the SHAKE algorithm with a geometric tolerance of 0.0001. Initial velocities were taken from the Maxwell-Boltzmann distribution at 300 K and were acquired for 200 ns.

Crystallography methods

Protein expression, refold and purification of MAIT TCRs and MR1 – Soluble AF7 MAIT TCR (TRBV6-1) TCR were refolded from inclusion bodies and refolded as described previously.[2] Human MR1-β2m was refolded in the presence of 2-hydroxy-5-methoxybenzaldehyde, and empty MR1-β2m complex was prepared as described previously.[3] Three steps of protein purification were conducted by sequential DEAE anion exchange, S200 15/60 column size exclusion chromatography and finally HiTrap-Q HP anion exchange. Protein purity was assessed using SDS-PAGE and concentrations were calculated from A280 nm values measured using a NanoDrop-spectrophotometer.

Ternary complexes crystallisation and structure determination and refinement – AF7-TCR was mixed with empty MR1-β2m and compound **6** in 1:1:1 ratio at a concentration of 4-5 mg/ml, and kept on ice for 1 h. Crystals of the ternary complexes were obtained by hanging drop crystallisation with a precipitant consisting of 100 mM BTP (pH 6.0 - 6.5), 8-20% PEG3350 and 200 mM sodium acetate, as established previously.[4] Hexagonal crystals formed over a week in dark conditions at 20 °C and were further soaked in reservoir solution with 12% glycerol for cryo-protection, then flashed frozen in liquid nitrogen. X-ray diffraction data were collected at 100 K on the Australian Synchrotron at MX1 beamlines. Diffraction images were indexed, integrated and scaled using XDS,[5] and then further processed using programs from the Phenix package[6] and CCP4 suite[7]. Phases were calculated by molecular replacement using PHASER,[8] where a modified TCR-MR1 ternary complex (PDB; 4L4T) was used as search model. Manual model building was performed in COOT,[9] which was interspersed with iterative rounds of refinement using Phenix.refine[6]. The Grade Webserver and Phenix tools were used to build the ligand and to generate ligand restraints. The models were validated using MolProbity[10] and graphical representations were generated using PyMOL Molecular Graphics System, Version 2, (Schrödinger, LLC, New York, NY).

Biological assay methods

Aqueous stability – Compound aqueous stability assay was performed as previously described.[1] A solution of compound (0.1 mM) in phosphate buffered saline (pH 7.4) was incubated at 37 °C. Aliquots (0.001 mL) were analysed by HPLC (0 to 100 % solvent B in solvent A; solvent A = 0.1 % trifluoroacetic acid in water, solvent B = 0.1 % trifluoroacetic acid in 10 % acetonitrile/water) at various time points, as indicated in the figure. Relative concentrations were quantified by measuring the area under the curve of the compound peaks at the absorption wavelength of 315 nm.

MR1 cell surface upregulation assay – MR1 surface expression upregulation assays were performed essentially as previously reported.[11] $1x10^5$ C1R cells (RRID:CVCL_3714) over-expressing MR1 (C1R.MR1 cells)[12] were incubated in U-bottom 96 well plates in RPMI media containing 10% fetal calf serum with test compounds, at a range of concentrations as indicated, for 16 h at 37 °C. Cells were then stained with 7-aminoactinomycin D (7AAD, BD) and MR1-PE (26.5, Biolegend, 1:200) or with biotinylated anti-MR1 mAb 26.5 (in-house) followed by PE-conjugated streptavidin (1:1000) before data acquisition on a BD Fortessa flow cytometer (BD Biosciences). MR1 expression was analysed on gated live (7AAD negative) C1R.MR1 cells. Data analysis was conducted using FlowJo software (version 10).

Jurkat.MAIT cell activation assay – MAIT cell reporter activation assays were performed essentially as previously described.[12] Jurkat-76 cells[13] (a sub-line of Jurkat E6.1; RRID:CVCL_0367) overexpressing the MAIT TCR clone AF7 (Jurkat.MAIT cells)[12] were co-incubated with C1R.MR1 cells (1×10^5 each cell line) and test compounds for 16 h at 37 °C. Cells were subsequently stained with PE-Cy7-conjugated anti-CD3 (UCHT1, eBioscience, 1:300), and APC-conjugated anti-CD69 (FN50, BD, 1:50) and 7AAD or fixable viability dye (FVD, 1:1000) before data acquisition on a BD Fortessa flow cytometer (BD Biosciences). Activation of Jurkat.MAIT cells was measured by an increase in surface CD69 expression on gated live Jurkat.MAIT cells (CD3+).

General chemical methods

***Chemicals* –** Anhydrous diethyl ether, tetrahydrofuran and dichloromethane were purchased from Sigma-Aldrich, then stored and dispensed from an Innovative Technology PureSolv solvent purification system. All other chemicals were used as purchased without further purification.

***Purification* and analysis –** HPLC mobile phases were defined as follows: solvent A was MilliQ H_2O with 0.1% trifluoroacetic acid, and solvent B was MeCN–H_2O (90:10, v/v) with 0.1% trifluoroacetic acid. LCMS mobile phases were defined as follows: solvent A was MilliQ H_2O with 0.1% formic acid, and solvent B was MeCN–H_2O (90:10, v/v) with 0.1% formic acid. LCMS analysis was carried out on a Shimadzu LCMS-2020 system equipped with a Phenomenex Luna 3 micron C18(2) 150 × 2.00 mm column (P/No 00F-4251-BO) and a SPD-M20A diode array detector.

***Characterisation* –** Electrospray ionisation high- resolution mass spectra (ESI-HRMS) measurements were obtained on a Bruker micrOTOF mass spectrometer equipped with an Agilent 1100 Series LC/MSD mass detector in positive ion mode by direct infusion in water at 100 µL/h using sodium formate clusters as an internal calibrant. ^{1}H and ^{13}C NMR spectra were recorded on Bruker Avance 600 MHz spectrometers at 298 K in deuterated solvents indicated and referenced to tetramethylsilane (^{1}H δ 0.00 for $CDCl_3$) or residual solvent signals (^{13}C: δ 77.0 for $CDCl_3$; $^{1}H/^{13}C$: δ 3.31/49.0 for CD_3OD).

Synthetic procedures

*Synthesis of compound **7***

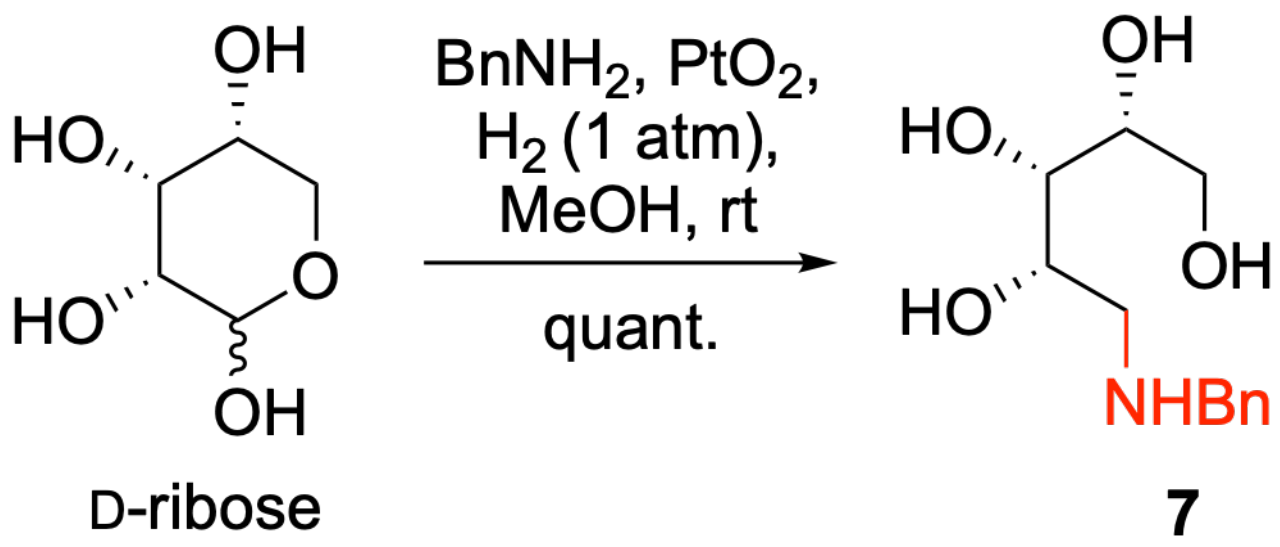

Adapting a procedure by Davoll,[14] a solution of D-ribose (911 mg, 6.07 mmol) and freshly distilled benzylamine (0.662 mL, 6.07 mmol) in methanol (6.6 mL) was stirred for 24 h at room temperature. After adding platinum dioxide (Adam's catalyst, 34 mg, 0.15 mmol), the reaction vessel was twice purged and filled with hydrogen gas using a balloon. The mixture was stirred at room temperature under the hydrogen atmosphere for 24 h, at which point LCMS indicated complete consumption of the imine intermediate. The mixture was filtered through celite under nitrogen, eluted with methanol, then concentrated *in vacuo* to give compound **7** as a viscous gum (1.70 g, quant.) that was used in the next step without further purification.

^{1}H NMR (600 MHz, CD_3OD): δ 7.34 – 7.31 (m, 4H), 7.27 – 7.24 (m, 1H), 3.88 (m, 1H), 3.81 (d, J = 13.0 Hz, 1H), 3.77 – 3.73 (m, 2H), 3.66 – 3.61 (m, 2H), 3.55 (m, 1H), 2.90 (dd, J = 12.1, 4.3 Hz, 1H), 2.74 (dd, J = 12.3, 7.0 Hz, 1H).

^{13}C NMR (150 MHz, CD_3OD): δ 140.5, 129.5 (2× carbons), 128.3, 75.8, 74.1, 71.8, 64.7, 54.5, 52.1.

HRMS: m/z calcd for $C_{12}H_{20}NO_4^+$: 242.1387 [M+H]$^+$; found: 242.1386.

Synthesis of compound ***8***

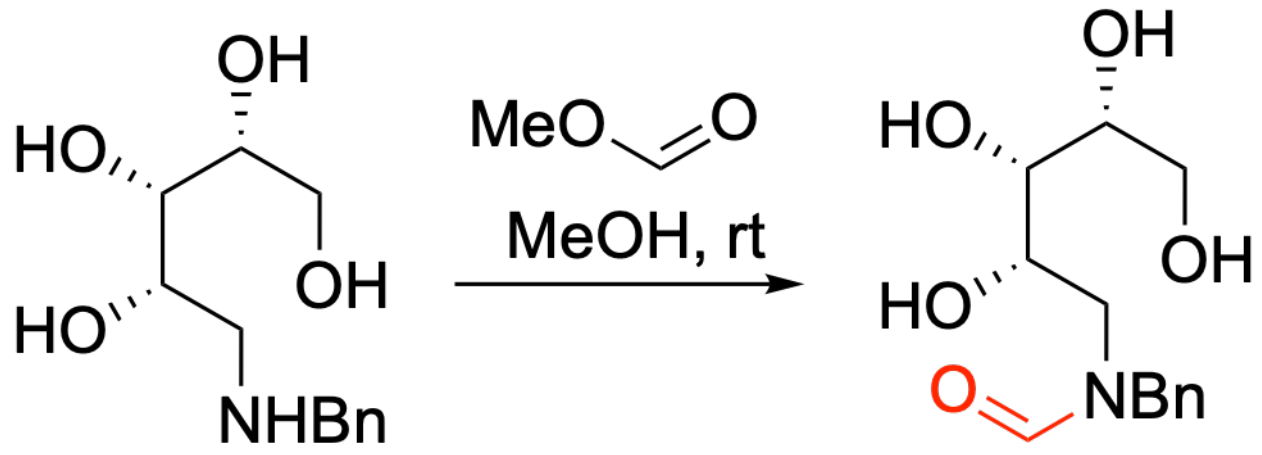

7 **8**

To a solution of amine **7** (1.70 g from previous step, 6.07 mmol assumed) in methanol (39 mL) was added methyl formate (2.9 mL, 47 mmol). The reaction was stirred at room temperature for 7 h, at which point amine **7** was completely consumed, as indicated by the NMR spectrum of a reaction aliquot. The solvents were concentrated *in vacuo* to give compound **8** as a mixture of *cis* and *trans* amides that was used in the next step without further purification.

^{1}H NMR (600 MHz, CD_3OD): δ 8.38 (s, 0.33H, CHO) and 8.19 (s, 0.67H, CHO), 7.41 – 7.25 (m, 5H, Ar), 4.92 (d, *J* = 14.8 Hz, 0.67H, $PhCH_2$) and 4.65 (s, 0.67H, $PhCH_2$) and 4.31 (d, *J* = 14.9 Hz, 0.67H, $PhCH_2$), 3.99 – 3.97 (m, 0.33H, CHCHCH), 3.88 (m, 0.66H, CHCHCH), 3.75 – 3.72 (m, 1H, CH_2OH), 3.69 – 3.62 (m, 1H, $CHCH_2OH$), 3.65 – 3.58 (m, 1H, CH_2OH), 3.52 (t, *J* = 6.3 Hz, 0.67H, $NHCH_2CH$) and 3.49 (t, *J* = 6.7 Hz, 0.33H, $NHCH_2CH$), 3.44 – 3.34 (m, 1.33H, NCH_2) and 3.44 – 3.41 (m, 0.67H, NCH_2).

^{13}C NMR (150 MHz, CD_3OD): δ 166.4 (CHO), 166.2 (CHO), 138.0 (q Ar), 137.8 (q Ar), 129.9 (Ar CH), 129.7 (Ar CH), 129.2 (Ar CH), 129.0 (Ar CH), 128.6 (Ar CH), 74.54 ($NHCH_2CH$), 74.49 ($NHCH_2CH$), 74.25 ($CHCH_2OH$), 74.20 ($CHCH_2OH$), 72.42 (CHCHCH), 70.63 (CHCHCH), 64.61 (CH_2OH), 64.58 (CH_2OH), 53.83 ($PhCH_2$), 50.52 (NCH_2), 46.97 ($PhCH_2$), 45.84 (NCH_2).

HRMS: m/z calcd for $C_{13}H_{20}NO_5^+$: 270.1336 $[M+H]^+$; found: 270.1341.

*Synthesis of compound **9***

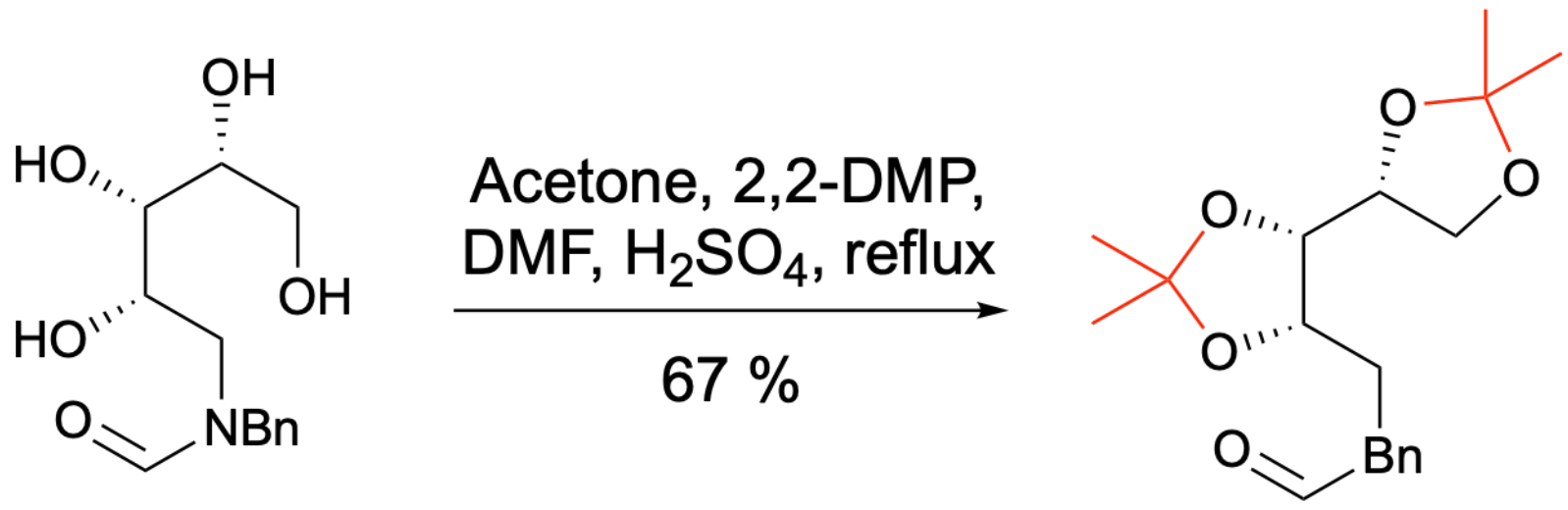

8 **9**

To a solution of tetraol **8** (4.95 g, 18.4 mmol) in acetone (300 mL) was added 2,2-dimethoxypropane (30 mL), dimethylformamide (30 mL), and then sulfuric acid (14 drops). The mixture was refluxed for 1.5 h. After cooling the mixture to room temperature, sodium hydrogen carbonate was added to neutralise the mixture, and then stirred at room temperature for 15 hours. The mixture was filtered, concentrated *in vacuo*, and then partitioned between petroleum spirit and water. The aqueous phase was extracted twice with petroleum spirit. The combined extracts were washed with brine, dried over magnesium sulfate, and then concentrated *in vacuo*. The crude product was purified by chromatography (silica gel, 15 to 40 % ethyl acetate/petroleum spirit) to give compound **9** as a pale yellow oil (4.31 g, 12.3 mmol, 67 %) as a mixture of *cis* and *trans* amides (approximately 1:1).

^{1}H NMR (600 MHz, $CDCl_3$): δ 8.32 (s, 0.5H, CHO) and 8.25 (s, 0.5H, CHO), 7.38 – 7.23 (m, 5H, Ar), 4.68 (d, *J* = 14.9 Hz, 0.5H $PhCH_2$) and 4.62 (d, *J* = 14.5 Hz, 0.5H, $PhCH_2$) and 4.59 (s, 1H, $PhCH_2$), 4.45 (m, 0.5H) and 4.18 (m, 0.5H), 4.10 – 3.85 (m, 5H), 3.58 (dd, *J* = 15.0, 2.2 Hz, 0.5H), 3.34 (dd, *J* = 15.0, 9.9 Hz, 0.5H) and 3.03 (dd, *J* = 14.3, 10.3 Hz, 0.5H), 1.43 (s, 1.5H, Me), 1.41 (s, 1.5H, Me), 1.363 (s, 1.5H, Me), 1.360 (s, 1.5H, Me), 1.30 (s, 1.5H, Me), 1.28 (s, 1.5H, Me), 1.25 (s, 1.5H, Me), 1.24 (s, 1.5H, Me).

^{13}C NMR (150 MHz, $CDCl_3$): δ 163.6 (CHO), 163.2 (CHO), 136.7, 136.3, 128.8, 128.6, 128.4, 127.9, 127.6, 127.5, 109.92, 109.85, 109.2, 109.0, 78.0, 77.8, 76.0, 75.8, 73.0, 68.0, 67.9, 52.2, 47.1, 46.4, 41.8, 28.0, 26.78, 26.75, 25.37, 25.35, 25.2

HRMS: m/z calcd for $C_{19}H_{28}NO_5^+$: 350.1962 $[M+H]^+$; found: 350.1960.

*Synthesis of compound **10***

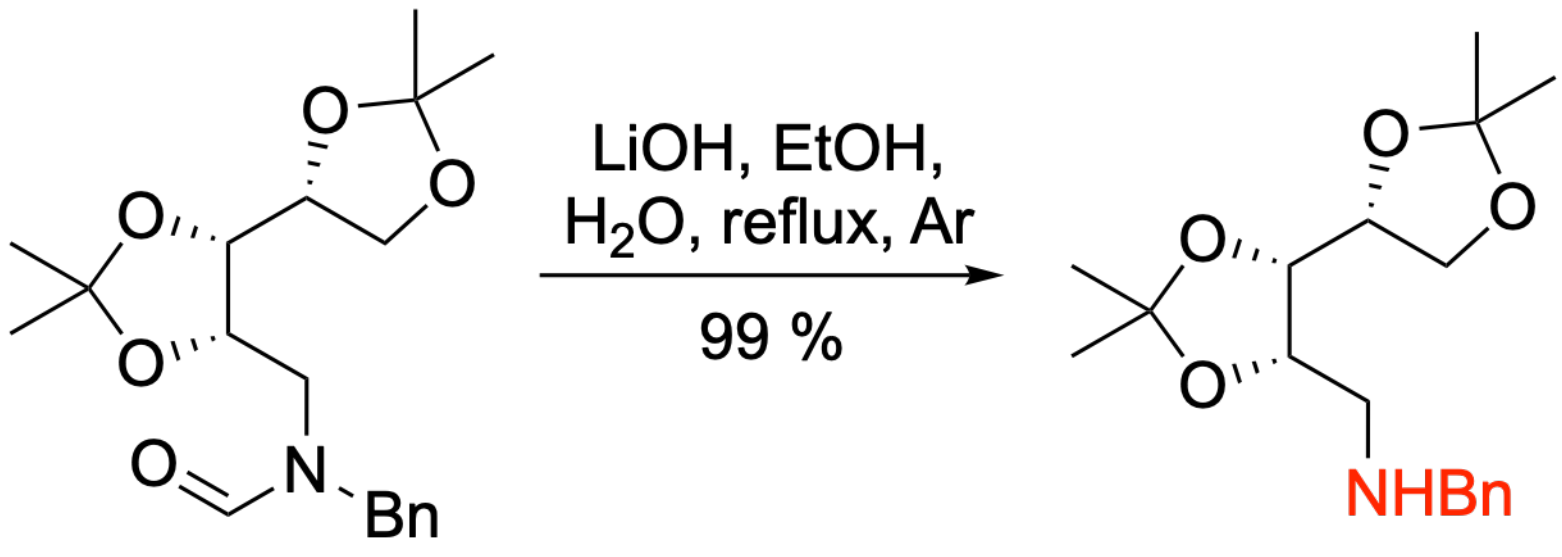

9 **10**

A mixture of compound **9** (4.31 g, 12.3 mmol), lithium hydroxide monohydrate (15.5 g, 369 mmol), ethanol (60 mL), and water (15 mL) was refluxed under argon for 42 hours, until the starting material was completely consumed. Ethanol was removed *in vacuo* at 50 °C, and then water was added. The mixture was extracted with ethyl acetate (× 3). The combined extracts were washed with brine, dried over magnesium sulfate, then evaporated *in vacuo* to give compound **10** as a pale yellow gum (3.92 g, 12.2 mmol, 99 %). The product was used in the next step without further purification.

^{1}H NMR (600 MHz, $CDCl_3$): δ 7.33 – 7.30 (m, 4H), 7.26 – 7.23 (m, 1H), 4.39 (m, 1H), 4.10 – 4.06 (m, 2H), 4.00 – 3.98 (m, 1H), 3.90 (m, 1H), 3.83 (s, 2H), 2.97 (dd, *J* = 12.1, 5.1 Hz, 1H), 2.85 (dd, *J* = 12.1, 7.8 Hz, 1H), 1.37 (s, 6H), 1.323 (s, 3H), 1.320 (s, 3H).

^{13}C NMR (150 MHz, $CDCl_3$): δ 140.3, 128.4, 128.1, 126.9, 109.7, 108.4, 78.3, 77.1, 73.3, 68.0, 54.3, 48.6, 28.0, 26.8, 25.5, 25.4.

HRMS: m/z calcd for $C_{18}H_{28}NO_4^+$: 322.2013 $[M+H]^+$; found: 322.2015.

*Synthesis of compound **11***

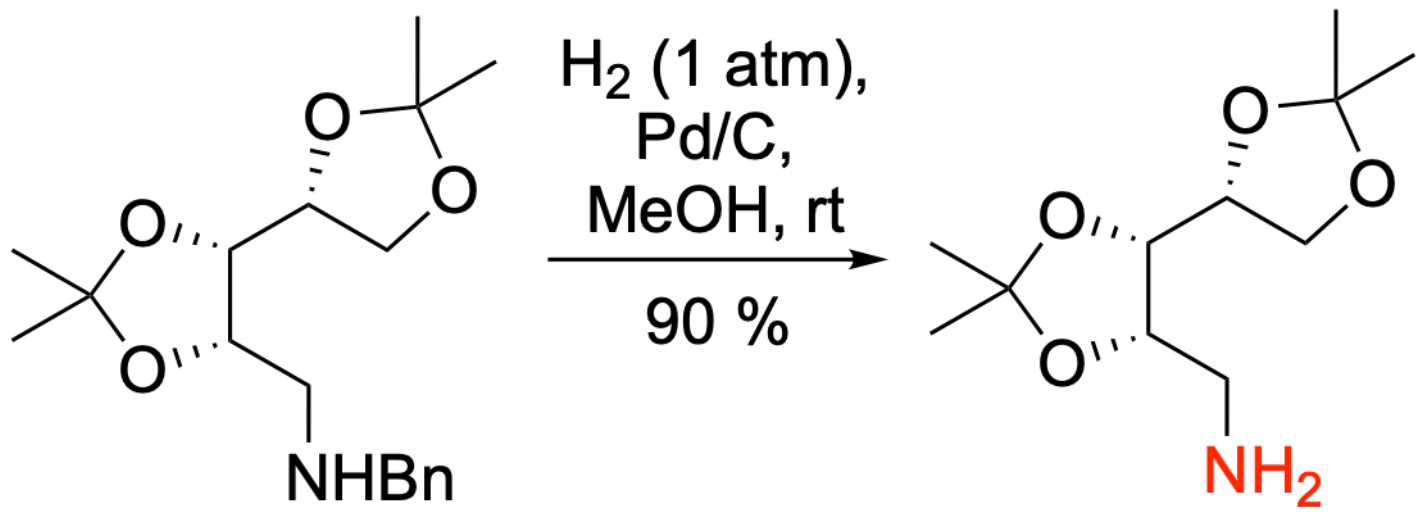

10 **11**

A mixture of compound **10** (3.92 g, 12.2 mmol), palladium on charcoal (10%, 400 mg) and methanol (80 mL) was stirred at room temperature under a hydrogen atmosphere (with a balloon). After 22 h, LCMS indicated complete consumption of compound **10**. The mixture was filtered and eluted with methanol through celite under nitrogen. The solvent was removed *in vacuo* to give compound **11** as a colourless oil (2.54 g, 11.0 mmol, 90 %), which was used in the next step without further purification.

^{1}H NMR (600 MHz, $CDCl_3$): δ 4.29 (m, 1H), 4.12 (m, 1H), 4.08 – 4.05 (m, 1H), 4.01 (dd, *J* = 9.4, 5.7 Hz, 1H), 3.92 (dd, *J* = 8.6, 5.5 Hz, 1H), 3.50 (br s, 2H), 3.11 (dd, *J* = 13.6, 5.3 Hz, 1H), 2.97 (dd, *J* = 13.1, 8.0 Hz, 1H), 1.42 (s, 3H), 1.40 (s, 3H), 1.35 (s, 3H), 1.34 (s, 3H).

^{13}C NMR (150 MHz, $CDCl_3$): δ 109.8, 108.7, 78.2, 78.1, 73.1, 68.0, 41.0, 28.0, 26.7, 25.38, 25.36.

HRMS: m/z calcd for $C_{11}H_{22}NO_4^+$: 232.1543 $[M+H]^+$; found: 232.1548.

*Synthesis of compound **12***

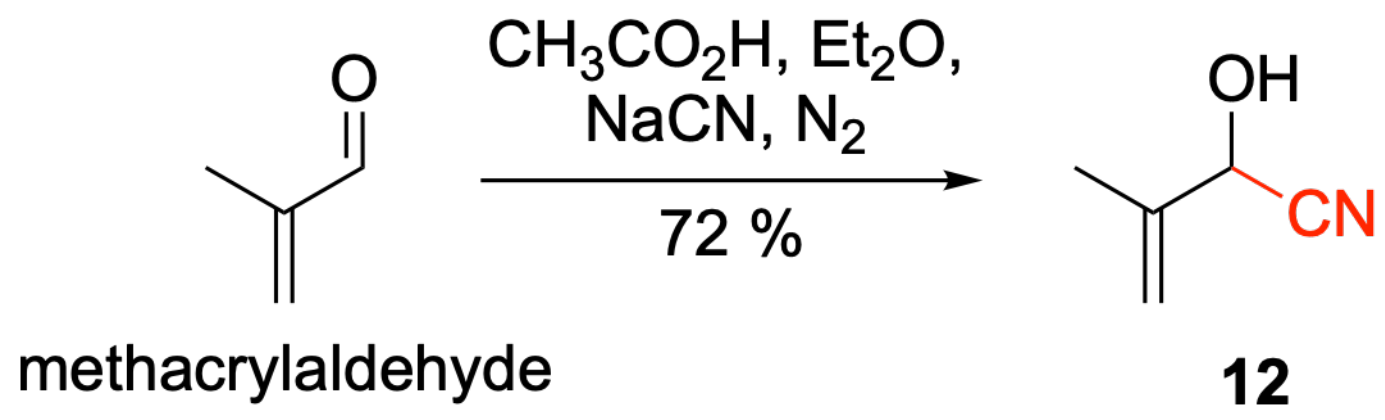

Warning – *highly toxic hydrogen cyanide gas is generated in this procedure*

Adapting a procedure by Sharma et al.,[15] sodium cyanide (4.96 g, 101 mmol) was dried in a round bottom flask under high vacuum at 100 °C for 2 h. After filling with argon and then keeping the flask under a positive pressure with a nitrogen balloon, dry diethyl ether (80 mL) was added. After cooling in an ice bath, glacial acetic acid (5.78 mL, 101 mmol) was added dropwise, and then the mixture was stirred for 30 min under nitrogen. Freshly distilled methacrylaldehyde (4.13 g, 59.0 mmol) was added dropwise to the mixture in an ice bath, and then allowed to warm slowly to room temperature overnight in the ice bath under nitrogen. After 64 h, sodium hydrogen carbonate was added to neutralise the mixture. The solids were removed by filtration and washed with diethyl ether. Careful concentration of the filtrate *in vacuo* at 30 °C with a rotary evaporator, and then brief exposure to high vacuum (to remove unreacted methacrylaldehyde), gave the cyanohydrin product **12** (4.10 g, 42.2 mmol, 72 %) as a pale yellow liquid. The NMR spectra matched that reported in the literature.[15]

^{1}H NMR (600 MHz, $CDCl_3$): δ 5.34 (s, 1H), 5.15 (s, 1H), 4.89 (br s, 1H), 2.41 (br s, 1H), 1.92 (br s, 3H).

^{13}C NMR (150 MHz, $CDCl_3$): δ 139.2, 118.0, 115.8, 65.2, 18.0.

*Synthesis of compound **13***

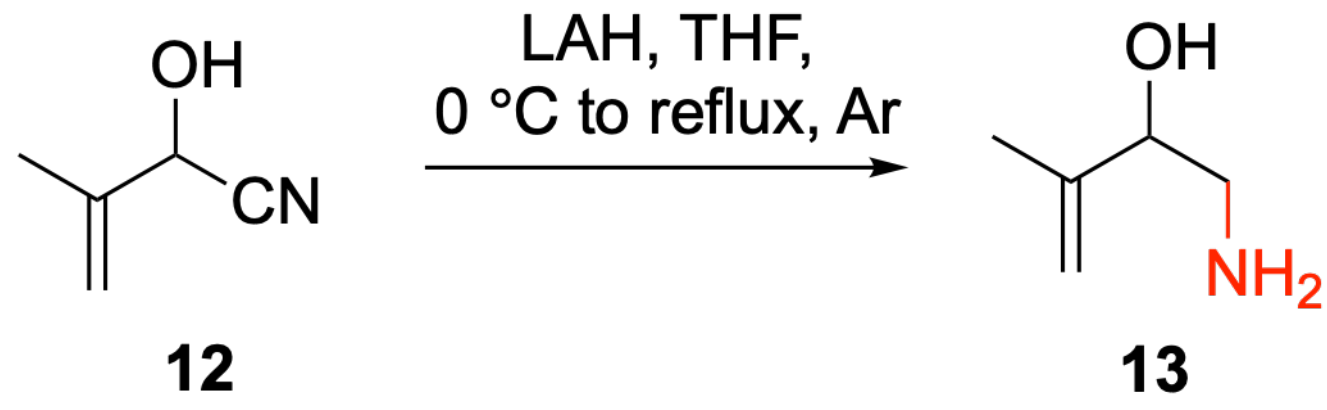

To a mixture of compound **12** (500 mg, 5.15 mmol) in dry tetrahydrofuran (10 mL) in an ice bath under argon was added lithium aluminium hydride (391 mg, 10.6 mmol) portionwise. After refluxing for 2 h under argon, TLC indicated complete consumption of compound **12**. The mixture was cooled in an ice bath, and then solid sodium sulfate decahydrate was added and stirred. After 30 min, 0.4 mL of 1.25 M sodium hydroxide solution was added. The solids were removed by filtration, and then washed with dichloromethane. The filtrate was concentrated *in vacuo* to give the compound **13** as a yellow oil (340 mg, 3.37 mmol, 65 %) that was used in the next step without further purification.

^{1}H NMR (600 MHz, $CDCl_3$): δ 5.03 (s, 1H), 4.92 (s, 1H), 4.00 (m, 1H), 2.88 (dd, *J* = 12.6, 4.0 Hz, 1H), 2.70 (dd, *J* = 12.6, 6.9 Hz, 1H), 1.73 (s, 3H).

^{13}C NMR (150 MHz, $CDCl_3$): δ 145.4, 111.2, 75.4, 45.4, 18.6.

HRMS: m/z calcd for $C_5H_{12}NO^+$: 102.0913 [M+H]$^+$; found: 102.0917.

*Synthesis of compound **15***

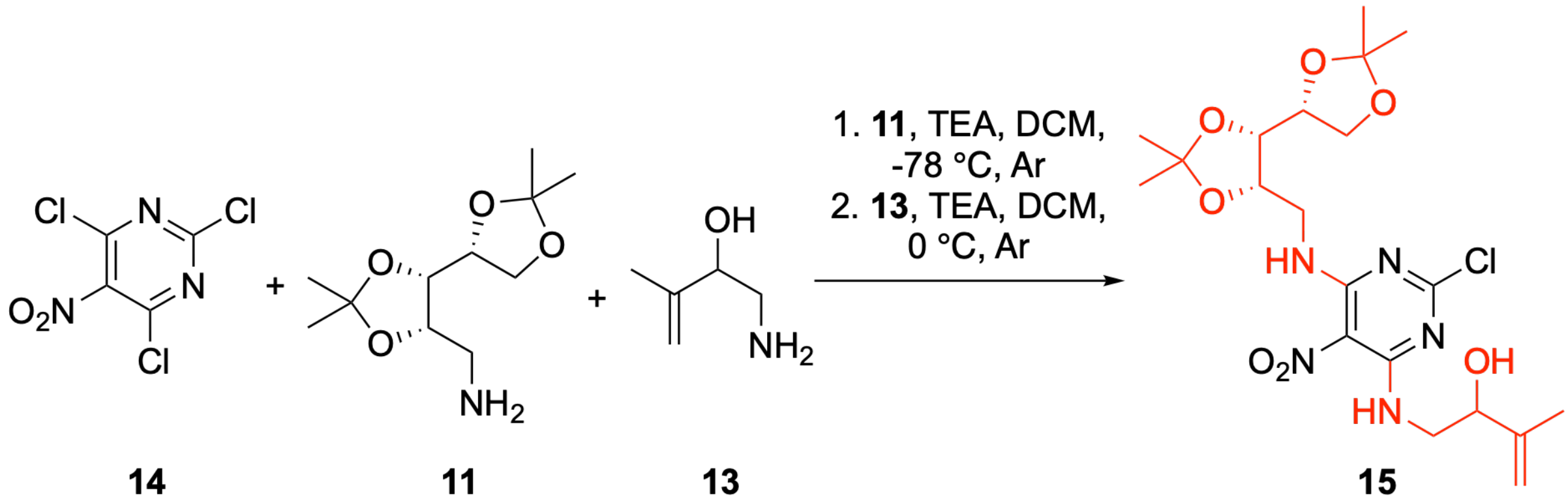

To a solution of 2,4,6-trichloronitropyrimidine (**14**, 104 mg, 0.455 mmol) in dry dichloromethane (1 mL) was successively added triethylamine (67 µL, 0.48 mmol) and a solution of ribitylamine **11** (111 mg, 0.478 mmol) in dry dichloromethane (1 mL) at -78 °C under argon. After 2 h, the reaction was warmed to 0 °C, at which point TLC indicated almost complete consumption of **14**. Triethylamine (67 µL, 0.48 mmol) and then a solution of **13** (52 mg, 0.51 mmol) in dry dichloromethane (0.5 mL) was added at 0 °C under argon. After 1 h, a further portion of **13** (20 mg, 0.20 mmol) in dry dichloromethane (0.25 mL) was added. The mixture was stirred at 0 °C for 40 min and then at room temperature for 1 h. Saturated ammonium chloride solution and water were added. The mixture was extracted with dichloromethane (× 3). The combined extracts were dried with magnesium sulfate, and then concentrated *in vacuo*. The crude product was purified by chromatography (silica gel, 10 to 30 % ethyl acetate/petroleum spirit) to give **15** as a pale yellow oil (100 mg, 0.205 mmol, 45 %).

^{1}H NMR (600 MHz, $CDCl_3$): δ 9.70 (t, *J* = 5.5 Hz, 1H), 9.56 (t, *J* = 5.0 Hz, 1H), 5.12 (s, 1H), 4.99 (s, 1H), 4.36 (m, 1H), 4.33 (m, 1H), 4.24 (m, 1H), 4.19 – 4.12 (m, 2H), 4.04 (dd, *J* = 9.4, 5.7 Hz, 1H), 3.92 (dd, *J* = 8.4, 5.7 Hz, 1H), 3.92 – 3.87 (m, 1H), 3.73 (ddd, *J* = 13.6, 7.2, 5.0 Hz, 1H), 3.67 – 3.63 (m, 1H), 2.54 (dd, *J* = 7.3, 3.5 Hz, 1H), 1.81 (s, 3H), 1.46 (s, 3H), 1.45 (s, 3H), 1.39 (s, 3H), 1.35 (s, 3H).

^{13}C NMR (150 MHz, $CDCl_3$): δ 162.4, 158.4, 158.0, 144.6, 112.4, 111.8, 110.5, 109.2, 78.5, 75.1, 73.97 and 73.95 (diastereomers), 73.1, 68.2, 45.86 and 45.83 (diastereomers), 40.7, 27.9, 26.5, 25.6, 25.4, 18.64 and 18.62 (diastereomers).

HRMS: m/z calcd for $C_{20}H_{31}ClN_5O_7^+$: 488.1907 [M+H]$^+$; found: 488.1909.

Synthesis of compound ***16***

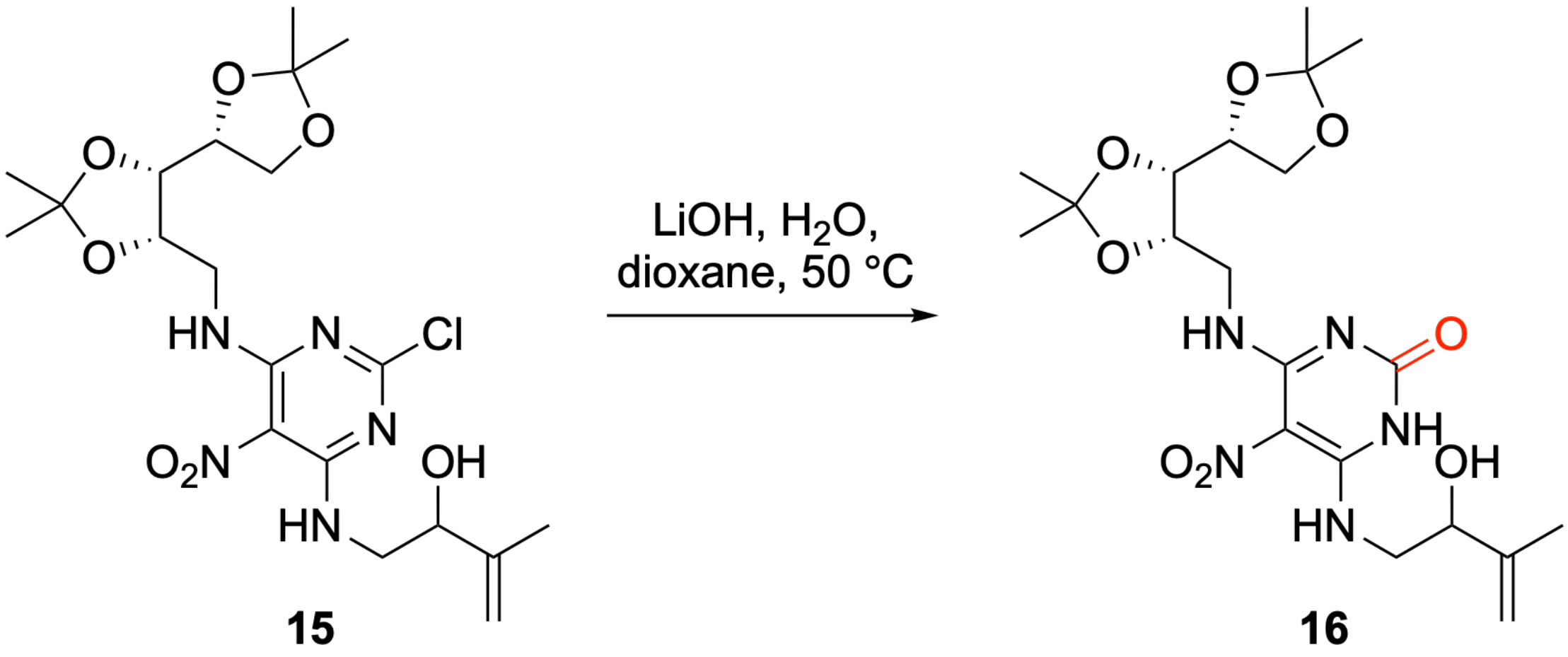

A mixture of **15** (37 mg, 0.076 mmol) in 1,4-dioxane (2.4 mL), water (1.2 mL) and lithium hydroxide monohydrate (265 mg, 6.32 mmol) was stirred under argon at 50 °C. After 1.5 h, TLC indicated complete consumption of **15**. Saturated ammonium chloride solution was added to approximately pH 7, and then the mixture was extracted with ethyl acetate (× 2). The combined extracts were washed with brine, dried over magnesium sulfate, and then concentrated *in vacuo* to give **16** as a pale yellow oil (34 mg, 0.072 mmol, 95 %) that was used in the next step without further purification.

^{1}H NMR (600 MHz, $CDCl_3$): δ 11.45 (br s, 1H), 10.19 (br s, 1H), 9.96 (br s, 1H), 5.16 (s, 1H), 5.03 (s, 1H), 4.42 – 4.36 (m, 2H), 4.19 – 4.14 (m, 2H), 4.12 – 4.03 (m, 2H), 3.92 (s, 1H), 3.83 – 3.72 (m, 3H), 1.82 (s, 3H), 1.44 (s, 6H), 1.37 (s, 3H), 1.35 (s, 3H).

^{13}C NMR (150 MHz, $CDCl_3$): δ 157.5 (br), 154.9 (br), 154.3, 143.6, 113.1, 110.4, 109.2, 107.0, 78.4, 74.9, 73.4, 73.0, 68.1, 46.5, 40.9, 27.9, 26.5, 25.5, 25.4, 18.5 and 18.4 (diastereomers).

HRMS: m/z calcd for $C_{20}H_{32}N_5O_8^+$: 470.2245 $[M+H]^+$; found: 470.2243.

*Synthesis of compound **17***

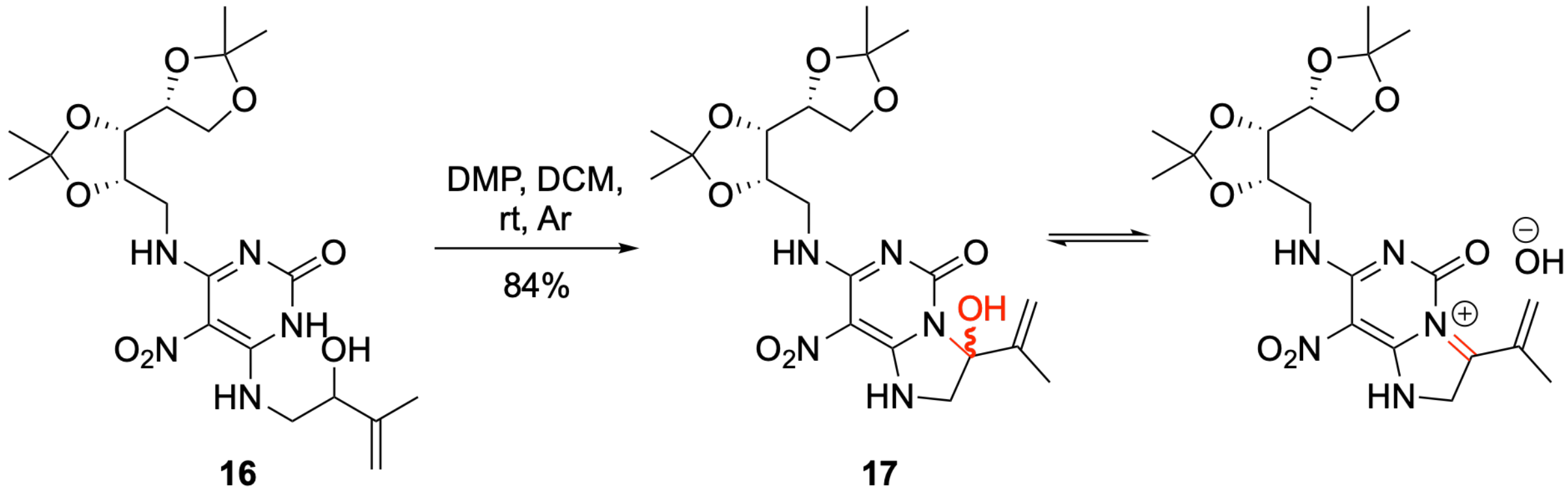

To a solution of alcohol **16** (70 mg, 0.15 mmol) in dry dichloromethane (1.6 mL) cooled in an ice bath under argon was added Dess-Martin Periodinane (66 mg, 0.16 mmol). The reaction was stirred in an ice bath for 1 h, and at room temperature for another 1 h, at which point LCMS indicated complete consumption of **16**. After adding saturated sodium hydrogen carbonate solution, the mixture was extracted with dichloromethane (× 3). The combined extracts were dried over magnesium sulfate, and then concentrated *in vacuo*. The crude product was carefully purified by chromatography (silica gel, 50 to 90 % ethyl acetate/petroleum spirit) to give **17** as a complex mixture of the diastereomeric hemiaminals and the corresponding ammonium salt (white solid, 59 mg, 0.13 mmol, 84 %), as characterised by ^{1}H, ^{13}C and HSQC NMR spectroscopy. Deconvoluted ^{1}H NMR spectral data listed here.

^{1}H NMR (600 MHz, $CDCl_3$): δ 9.34 (br t, *J* = 6.4 Hz, 0.3H, hemiaminal ribityl NH), 8.53 (s, 0.3H hemiaminal imidazolidinyl NH), 6.00 (br s, 1H, ammonium salt hydroxide), 5.40 (d, *J* = 12.5 Hz, 0.3H, hemiaminal alkene), 5.17 (d, *J* = 3.6 Hz, 0.3H, hemiaminal alkene), 4.88 (br s, 2H, ammonium salt alkene), 4.43 (br m, 1H, ammonium salt ribityl CH), 4.36 (p, *J* = 6.4 Hz, 0.3H, hemiaminal ribityl CH), 4.20 – 3.66 (m, 9H), 1.98 (s, 3H, ammonium salt CH_3), 1.83 (s, 1H, hemiaminal CH_3), 1.48 – 1.31 (m, 12H, isopropylidene CH_3).

HRMS: m/z calcd for $C_{20}H_{30}N_5O_8^+$: 468.2089 $[M+H]^+$; found: 468.2090.

*Synthesis of compound **18***

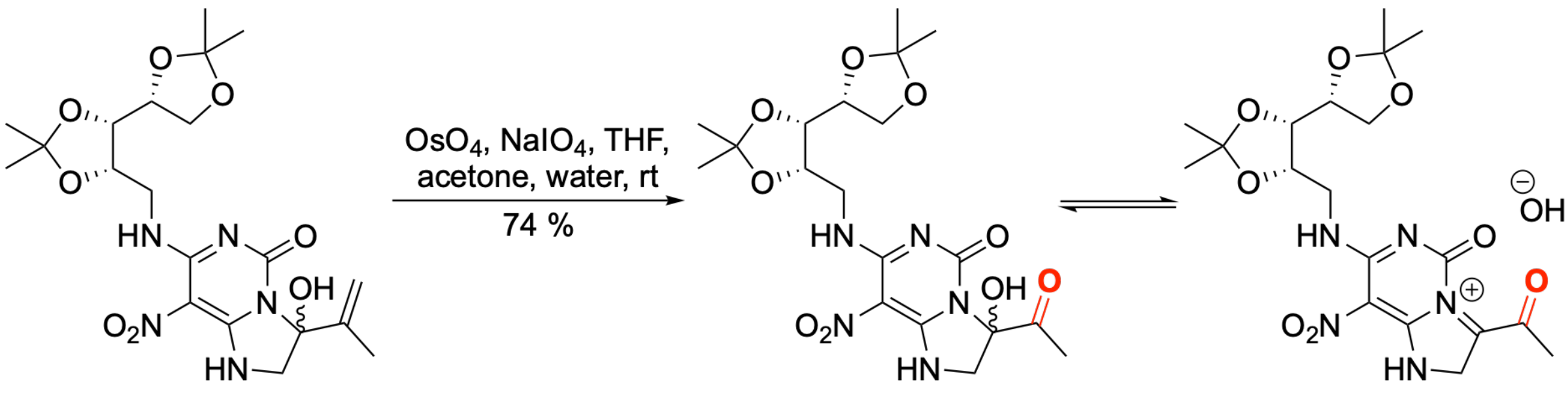

17 **18**

To a mixture of hemiaminal **17** (20 mg, 0.043 mmol) in tetrahydrofuran (0.74 mL), acetone (90 μL) and water (90 μL) was added osmium tetroxide (2.5 % solution in *tert*-butanol, 40 μL, 3.9 μmol), and then sodium periodate (28 mg, 0.13 mmol). After stirring at room temperature for 17 h, LCMS indicated complete consumption of the alkene. Sodium thiosulfate aqueous solution (10 % w/w) was added until the reaction was dark brown. The mixture was extracted with ethyl acetate (× 3). The combined extracts were washed with brine, dried over magnesium sulfate, and then concentrated *in vacuo*. The crude product was purified by chromatography (silica gel, 60 to 100 % ethyl acetate/petroleum spirit) to give a 1:1 diastereomeric mixture of ketone **18** (15 mg, 0.032 mmol, 74 %), which eliminated reversibly to form the corresponding ammonium salt upon prolonged standing. The NMR spectra of the hemiaminals below were obtained immediately after aqueous work up and chromatography.

^{1}H NMR (600 MHz, $CDCl_3$): δ 9.38 (q, *J* = 6.0 Hz, 1H), 8.77 (s, 0.5H) and 8.76 (s, 0.5H), 6.03 (s, 0.5H) and 5.91 (s, 0.5H), 4.37 (m, 1H), 4.19 – 4.10 (m, 3H), 4.08 (dd, *J* = 12.3, 3.8 Hz), 4.03 – 3.97 (m, 2H), 3.91 (ddd, *J* = 8.3, 5.8, 2.3 Hz, 1H), 3.71 (m, 1H), 2.47 (s, 1.5H) and 2.45 (s, 1.5H), 1.44 (s, 3H), 1.42 (s, 1.5H) and 1.41 (s, 1.5H), 1.39 (s, 1.5H) and 1.38 (s, 1.5H), 1.32 (s, 3H).

^{13}C NMR (150 MHz, $CDCl_3$): δ 202.0 and 201.6, 157.89 and 157.88, 154.67 and 154.65, 150.89 and 150.81, 110.41 and 110.37, 109.03 and 109.01, 106.0, 91.5 and 91.4, 78.45 and 78.40, 74.80 and 74.74, 73.0, 68.08 and 68.06, 54.7 and 54.6, 40.5 and 40.4, 27.9, 26.44 and 26.41, 25.55 and 25.52, 25.4, 24.8 and 24.6.

HRMS: m/z calcd for $C_{19}H_{28}N_5O_9^+$: 470.1882 [M+H]$^+$; found: 470.1879.

*Synthesis of compound **22***

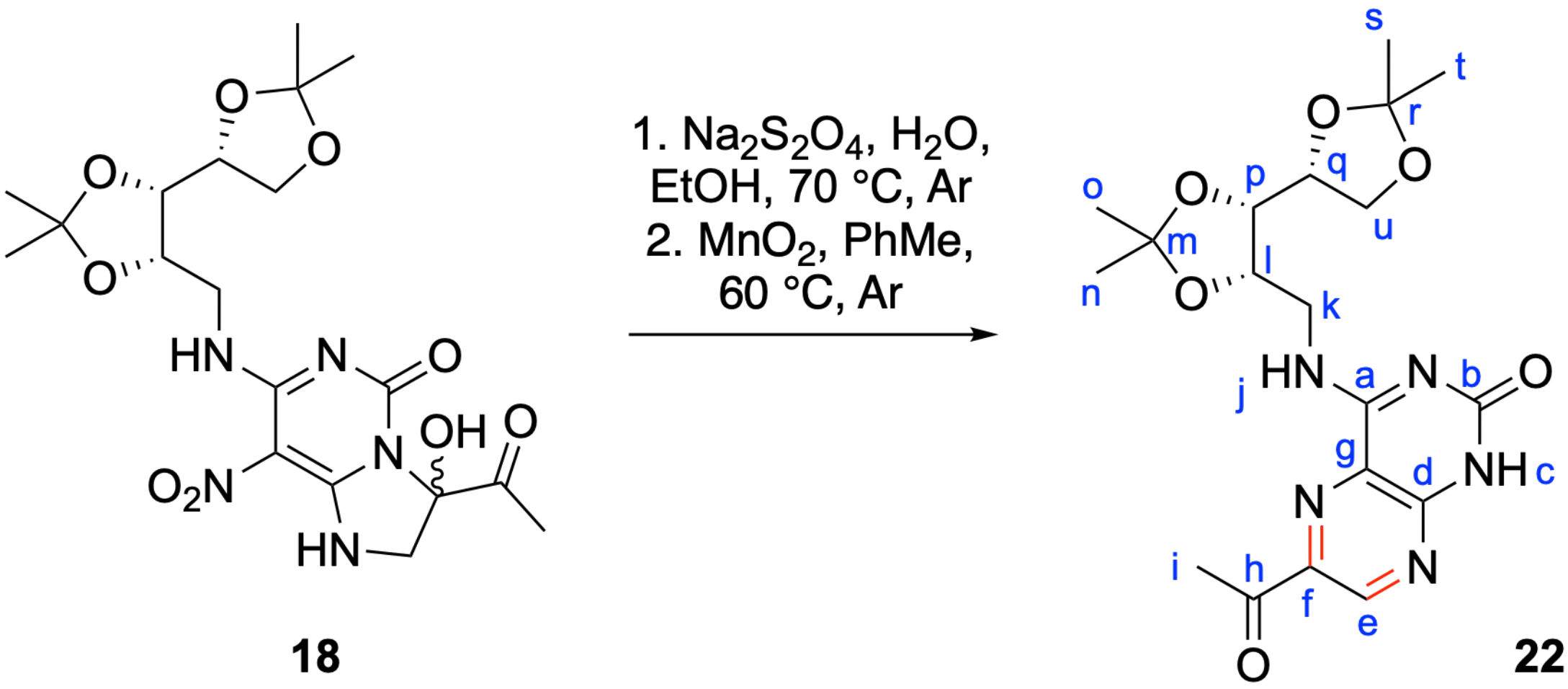

A solution of **18** (4 mg, 9 μmol) in ethanol (0.75 mL) and water (0.3 mL) was degassed with bubbling argon at 70 °C for 10 minutes. Sodium dithionite (12 mg, 69 μmol) was added, at which point the reaction became bright yellow. After 1 h of heating at 70 °C under argon, LCMS indicated complete consumption of starting material, and the reaction was colourless. Upon exposure to air, the reaction became bright yellow. The mixture was cooled to room temperature, diluted with water, and then extacted with ethyl acetate (× 3). The combined extracts were washed with brine, dried over magnesium sulfate, then concentrated *in vacuo*. The residue was dissolved in dichloromethane (5 mL), and then manganese dioxide (5 mg) was added. After stirring at room temperature for 17 h, LCMS indicated complete oxidation to **22**. The product mixture was filtered through celite, eluted with dichloromethane, concentrated *in vacuo*, and then purified by chromatography (silica gel, 66 to 100 % ethyl acetate/petroleum spirit) to give pyrazine **22** (1.5 mg, 3.6 μmol, 40 %).

^{1}H NMR (600 MHz, $CDCl_3$): δ 10.18 (s, 1H, c), 9.26 (s, 1H, e), 7.55 (t, *J* = 5.7 Hz, 1H, j), 4.51 (m, 1H, p), 4.18 – 4.14 (m, 2H, q & u), 4.13 – 4.07 (m, 2H, k & l), 4.02 – 3.94 (m, 2H, k & u), 2.73 (s, 3H, i), 1.48 (s, 3H, n/o), 1.44 (s, 3H, s/t), 1.37 (s, 6H, n/o & s/t).

^{13}C NMR (150 MHz, $CDCl_3$): δ 196.5 (h), 160.1 (a), 156.4 (b), 150.1 (d), 148.6 (e), 142.3 (f), 120.8 (g), 110.2 (r), 109.3 (m), 78.2 (l), 74.9 (p), 73.1 (q), 68.1 (u), 40.8 (k), 28.1 (n/o), 26.9 (s/t), 25.52 (n/o/s/t), 25.50 (n/o/s/t), 25.49 (i).

HRMS: m/z calcd for $C_{19}H_{26}N_5O_6^+$: 420.1878 [M+H]$^+$; found: 420.1866.

Synthesis of compound ***6***

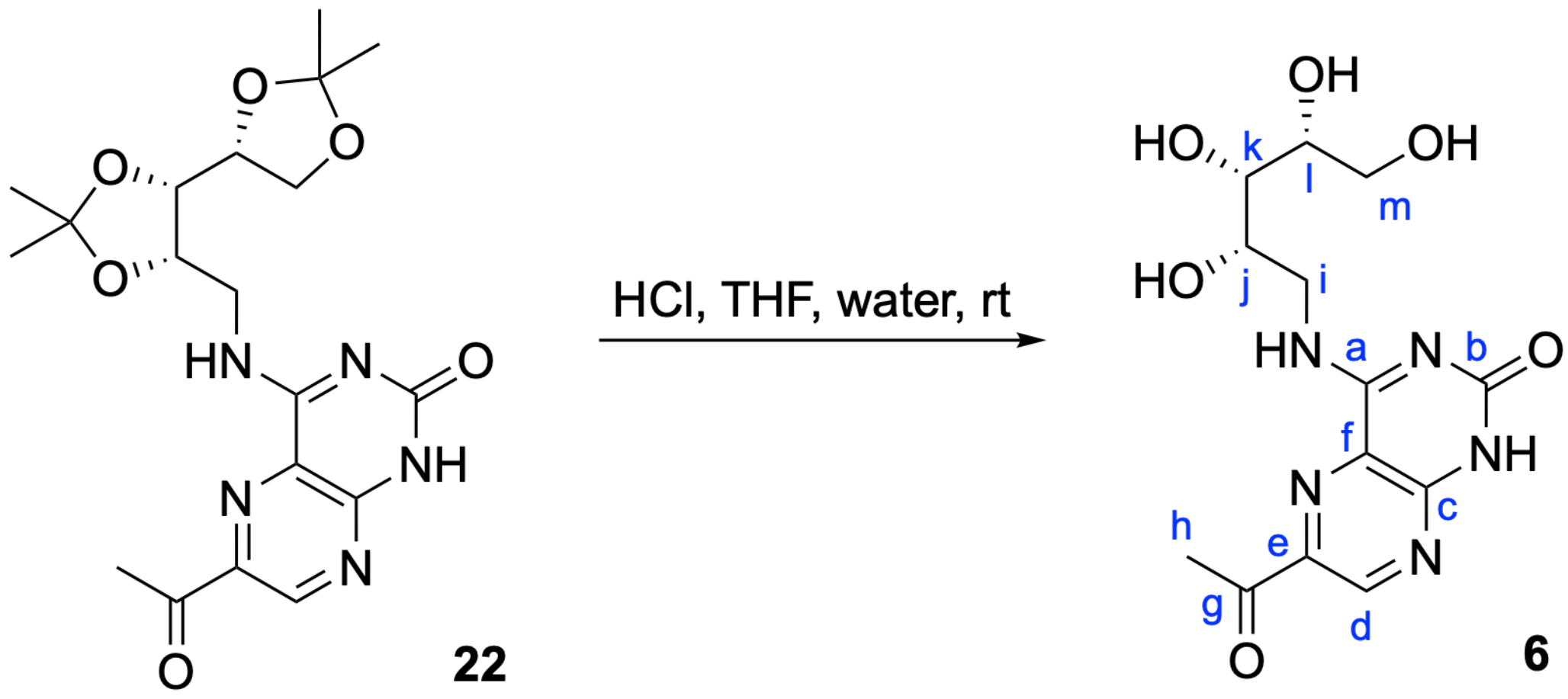

A solution of compound **22** (31 mg, 0.074 mmol) in tetrahydrofuran (11 mL) and aqueous hydrochloric acid (1 M, 11 mL) was stirred at room temperature for 4.5 h, at which point UPLC analysis indicated complete consumption of starting material. After evaporation of tetrahydrofuran *in vacuo*, the mixture was purified by reversed phase HPLC (0% to 100% solvent B over 40 min). Product containing fractions were diluted 3:1 v/v with ammonium acetate aqueous solution (20 mM, pH 5.4), and then concentrated *in vacuo* at 40 °C to approximately 2 mL in volume. Water was added, and then partially concentrated again *in vacuo*, repeating this process three times, to remove all of the acetonitrile. Compound **6** was obtained in 65 % (0.048 mmol), as determined by quantifying[16,17] the concentration of the aqueous solution by NMR spectroscopy.

^{1}H NMR (600 MHz, 10% D_2O/H_2O): δ 9.19 (s, 1H, d), 4.12 (m, 1H, k), 3.91 (dd, *J* = 14.3, 3.3 Hz, 1H, i), 3.84 (dd, *J* = 14.4, 7.3 Hz, 1H, i/m), 3.78 – 3.71 (m, 3H, j, l, & m/i), 3.59 (dd, *J* = 11.8, 6.5 Hz, 1H, m), 2.67 (s, 3H, h).

^{13}C NMR (150 MHz, 10% D_2O/H_2O): δ 199.8 (g), 158.1 (a), 152.2 (b), 149.8 (d), 149.1 (c), 143.3 (e), 121.0 (f), 73.0 (l), 72.3 (j), 69.5 (k), 62.6 (m), 45.1 (i), 25.2 (h).

HRMS: m/z calcd for $C_{13}H_{18}N_5O_6^+$: 340.1252 [M+H]$^+$; found: 340.1254.

NMR Spectra

^{1}H NMR spectrum (600 MHz, CD_3OD)

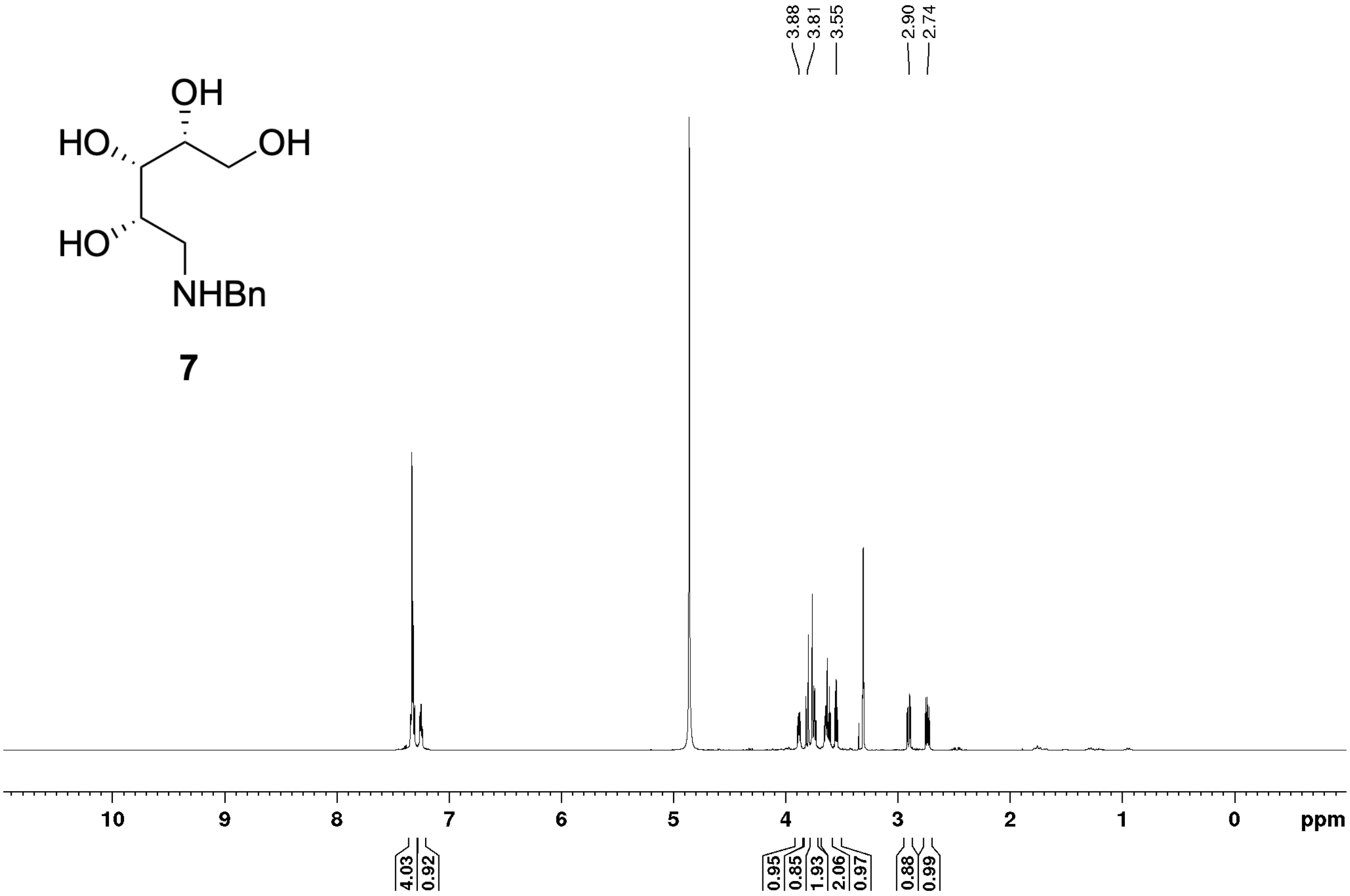

^{13}C NMR spectrum (150 MHz, CD_3OD)

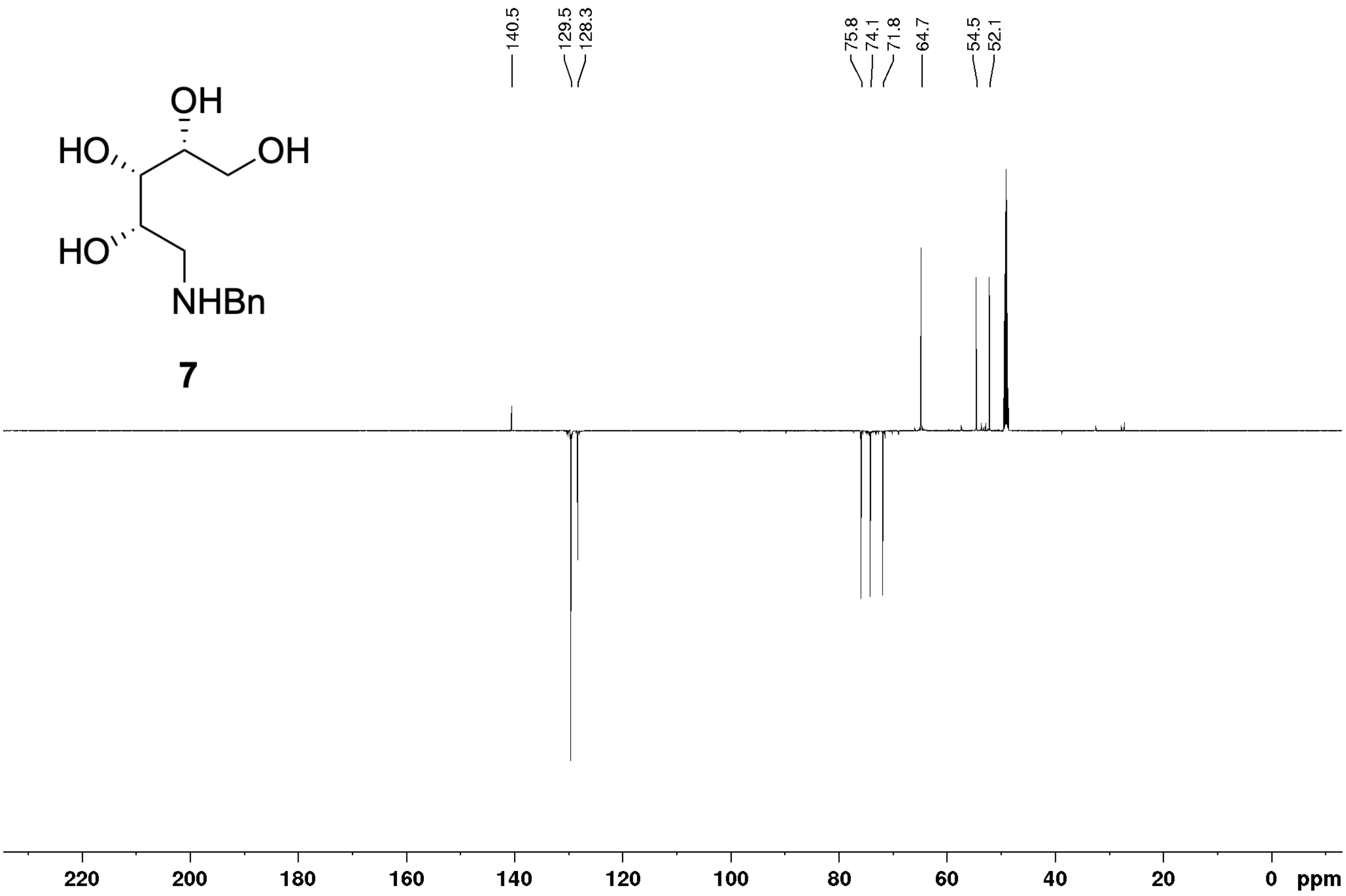

^{1}H NMR spectrum (600 MHz, CD_3OD)

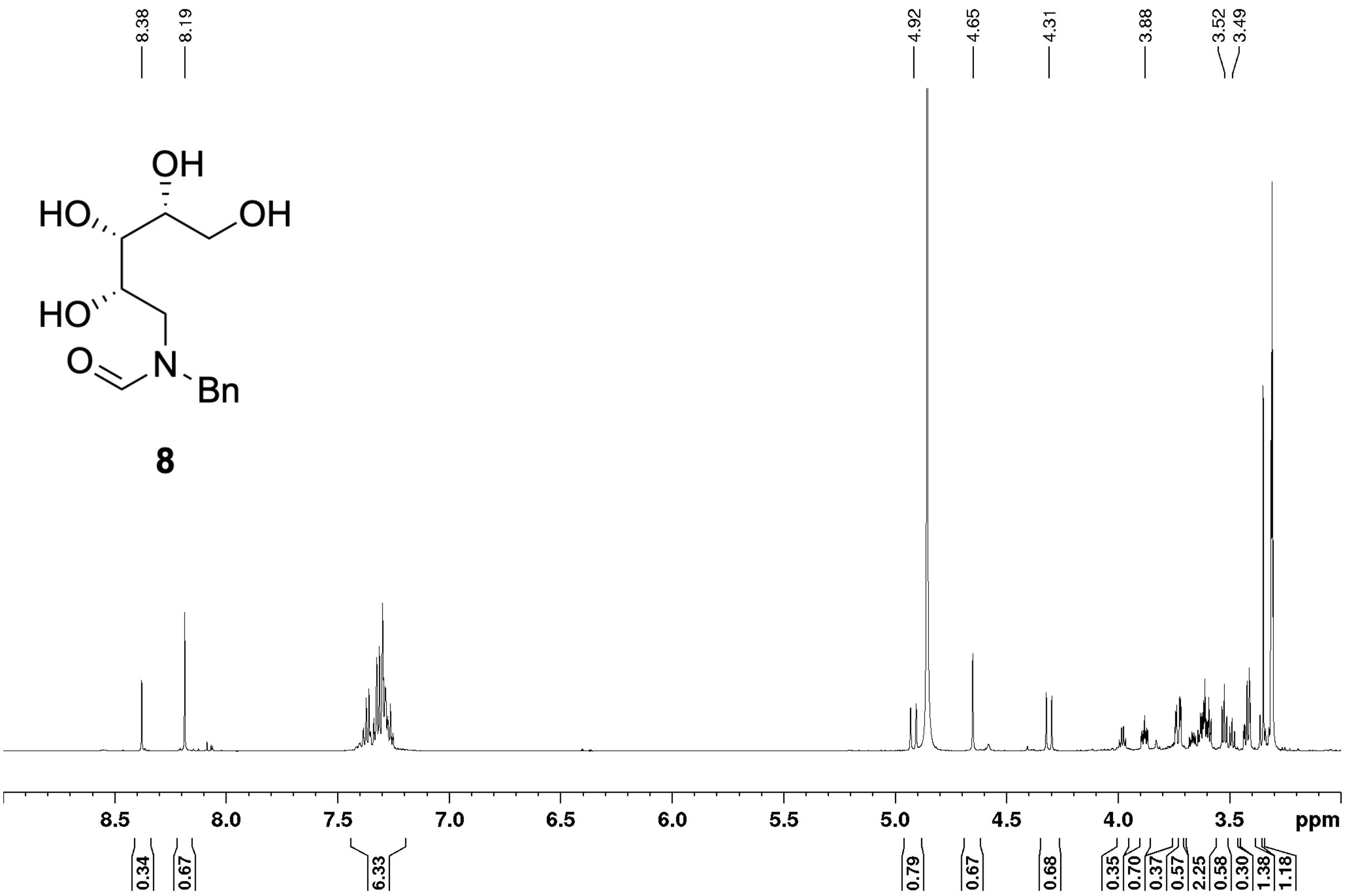

^{13}C JMOD NMR spectrum (150 MHz, CD_3OD)

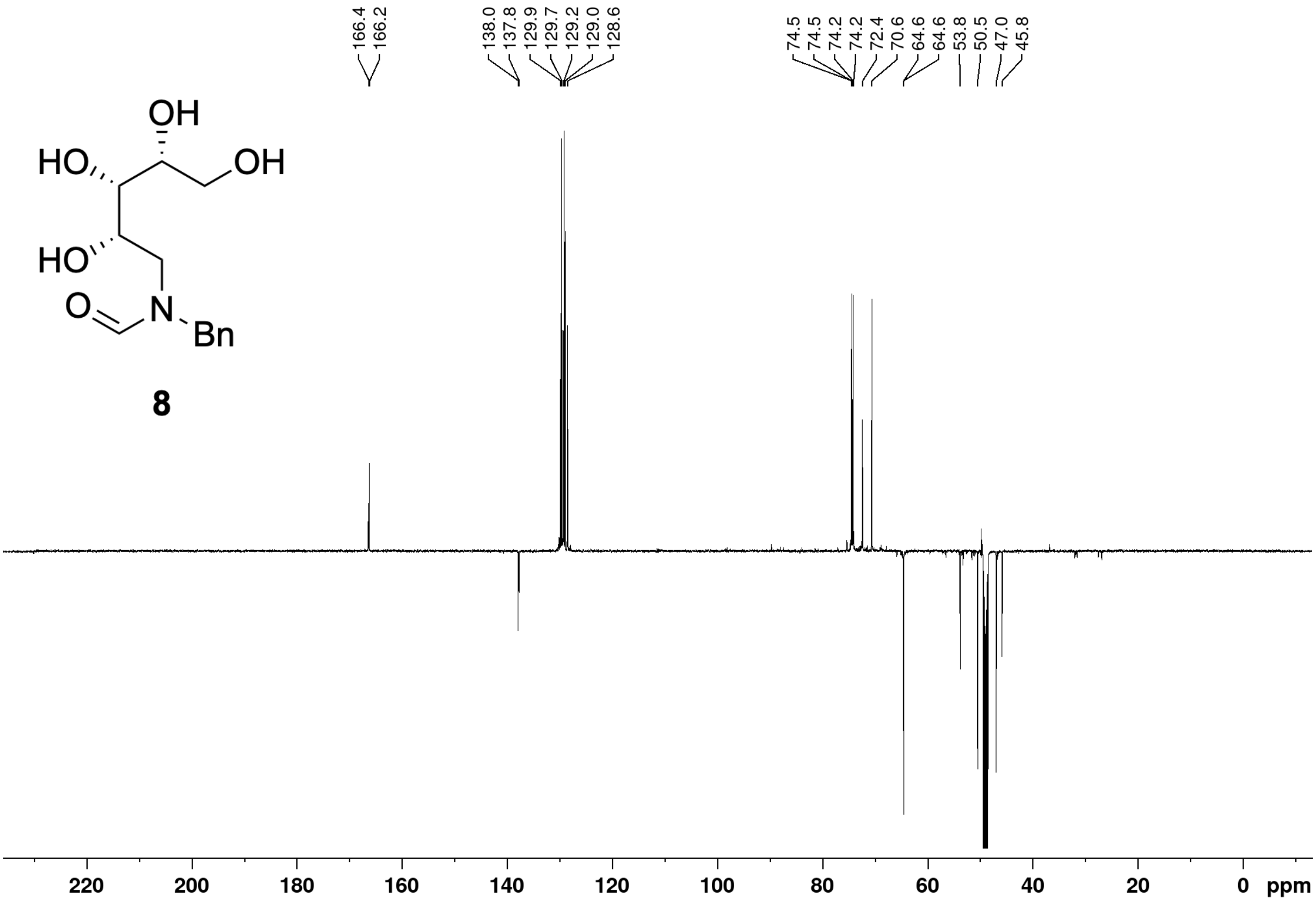

^{1}H NMR spectrum (600 MHz, $CDCl_3$)

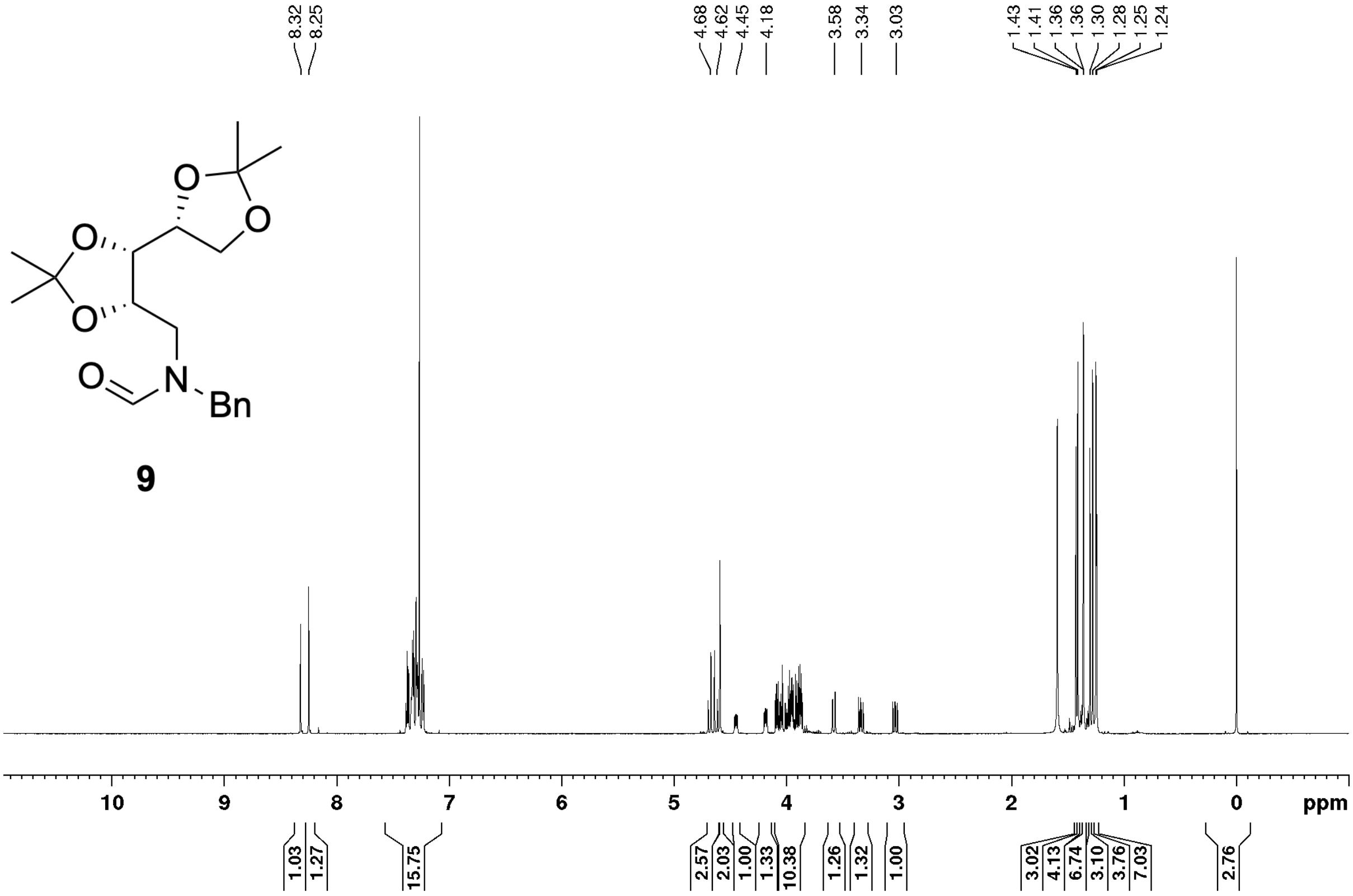

^{13}C NMR spectrum (150 MHz, $CDCl_3$)

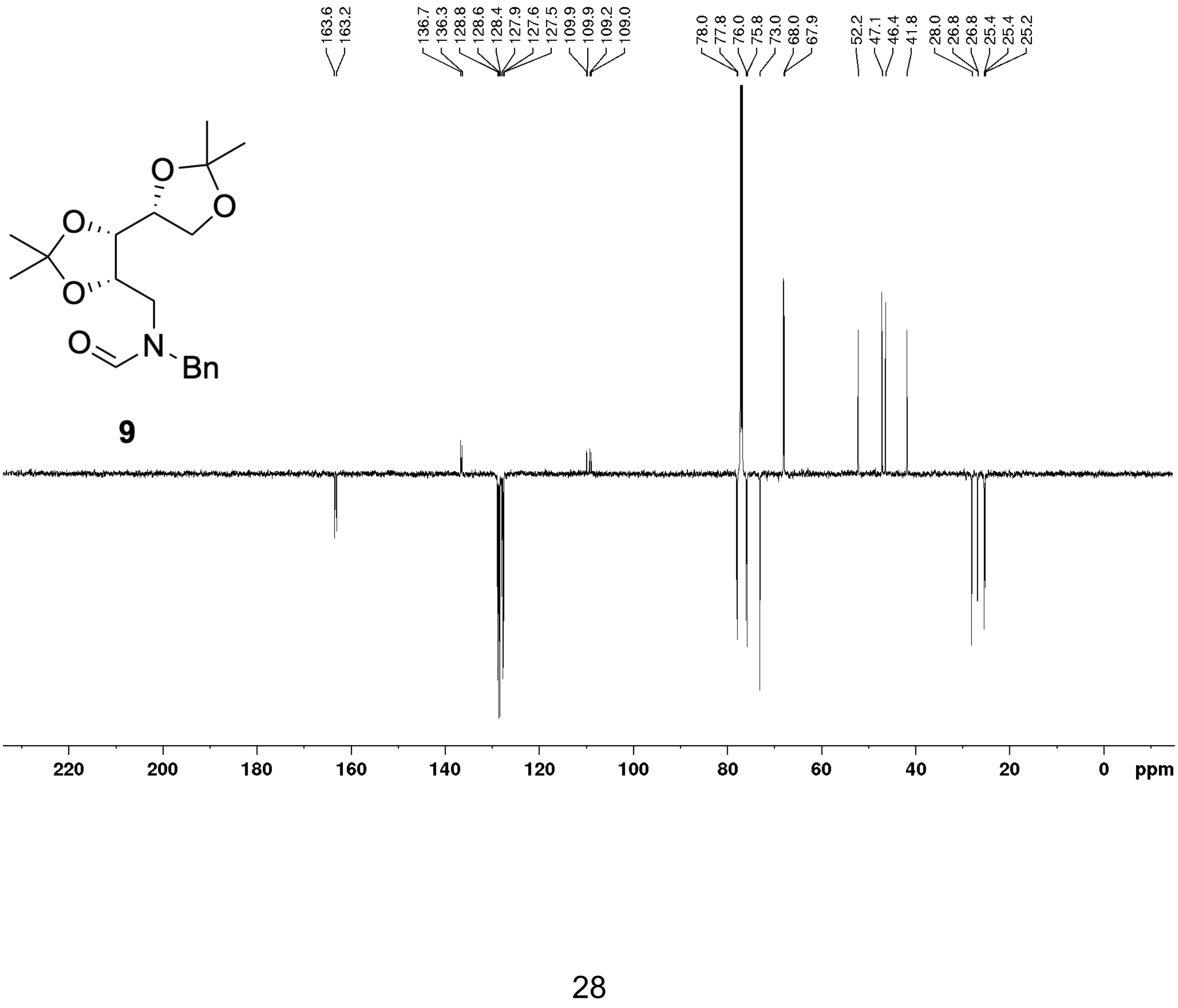

^{1}H NMR spectrum (600 MHz, $CDCl_3$)

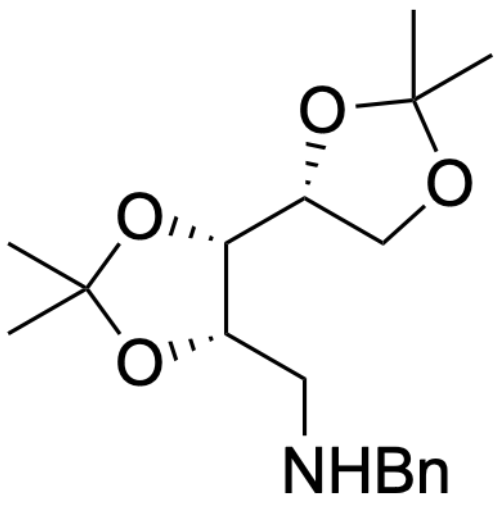

10

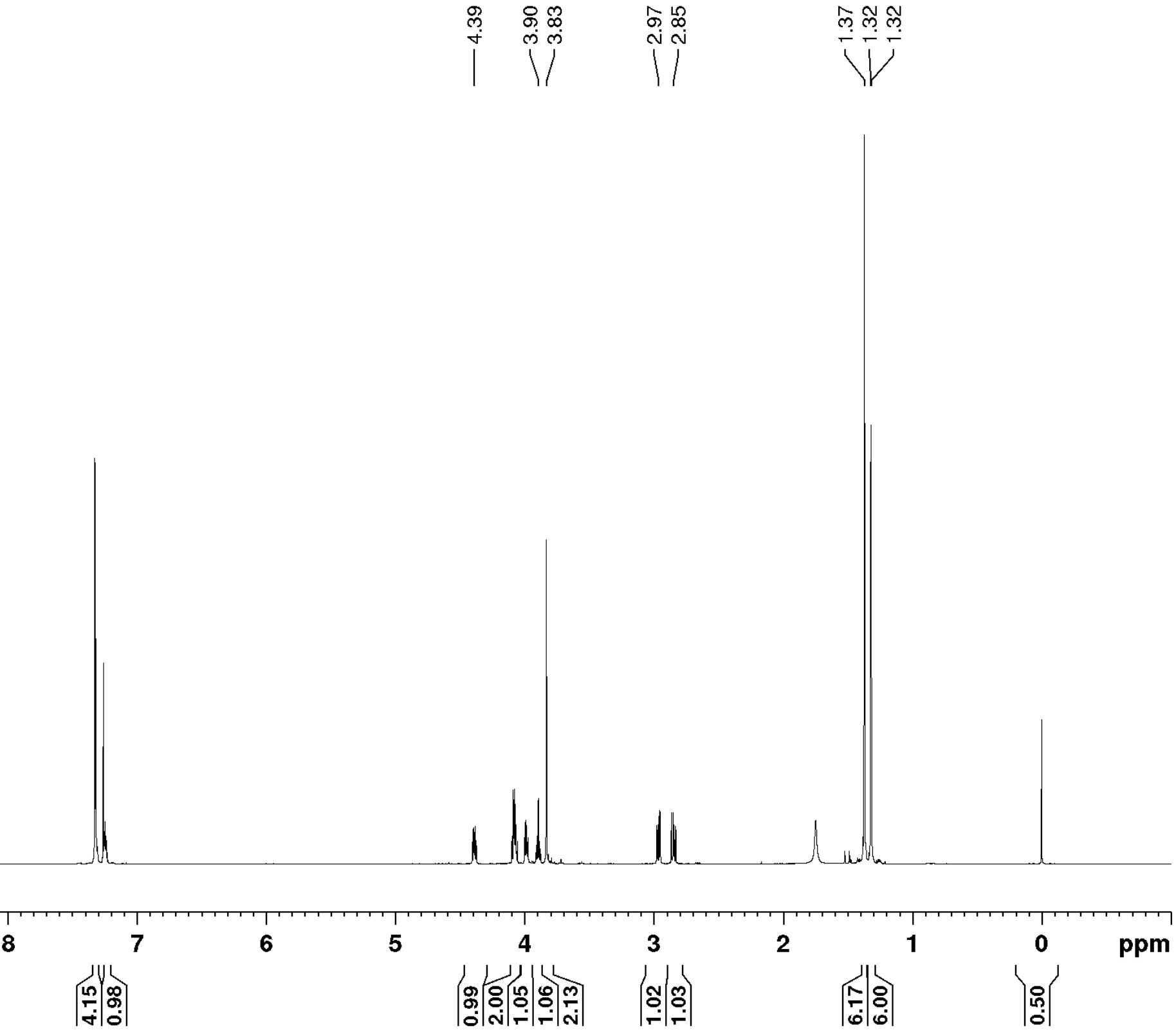

^{13}C NMR spectrum (150 MHz, $CDCl_3$)

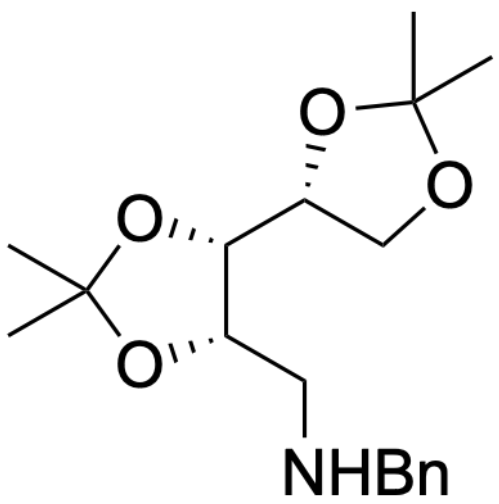

10

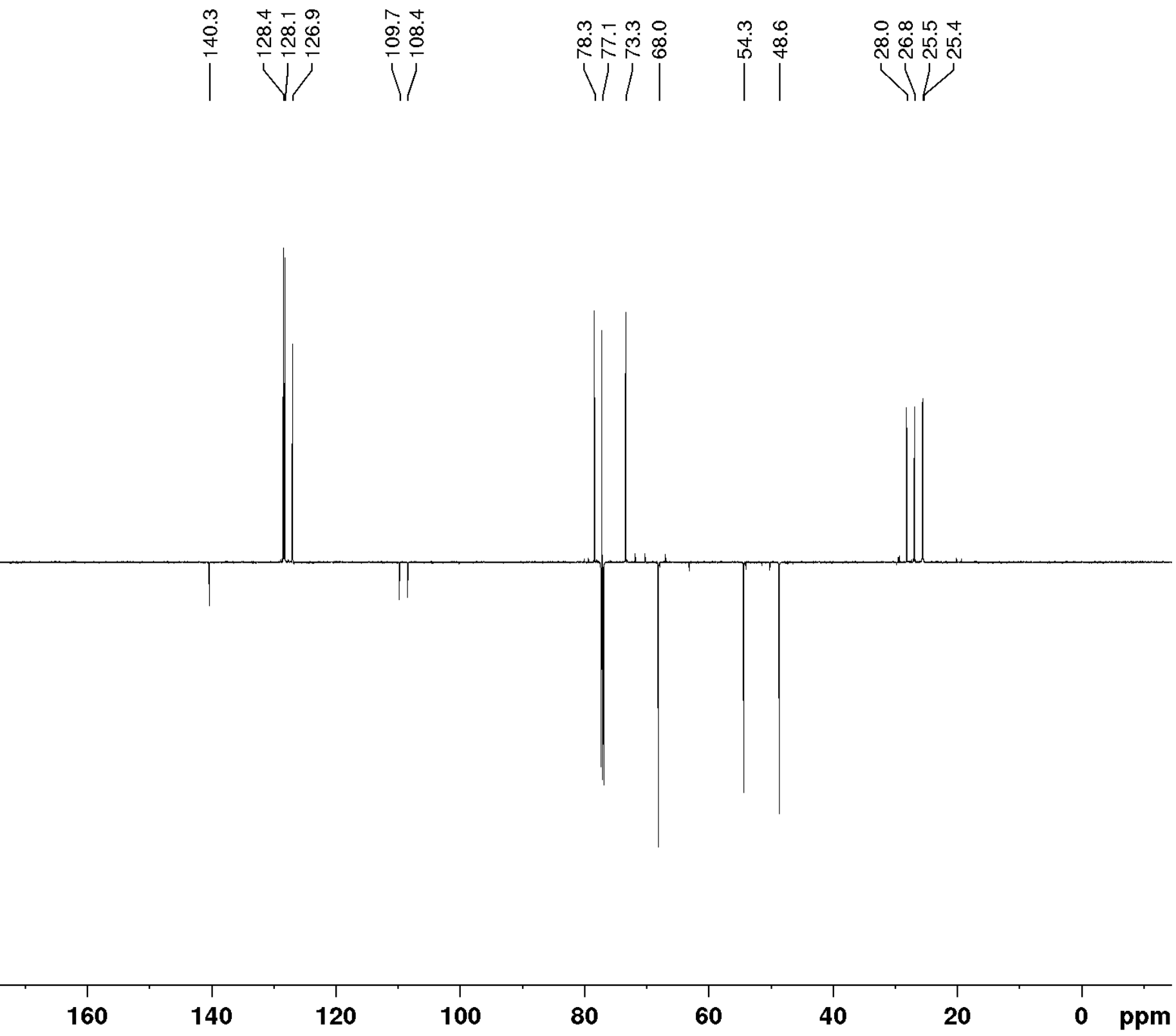

^1H NMR spectrum (600 MHz, $CDCl_3$)

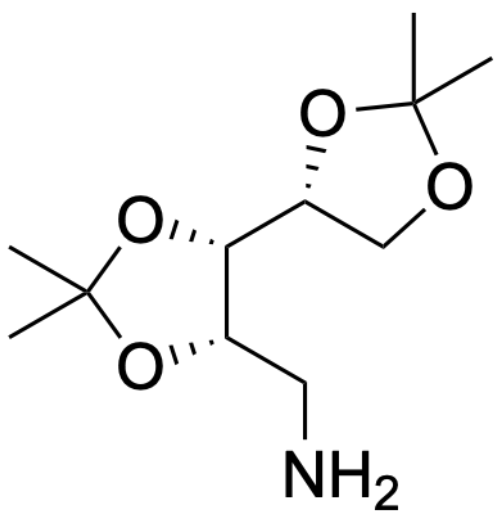

11

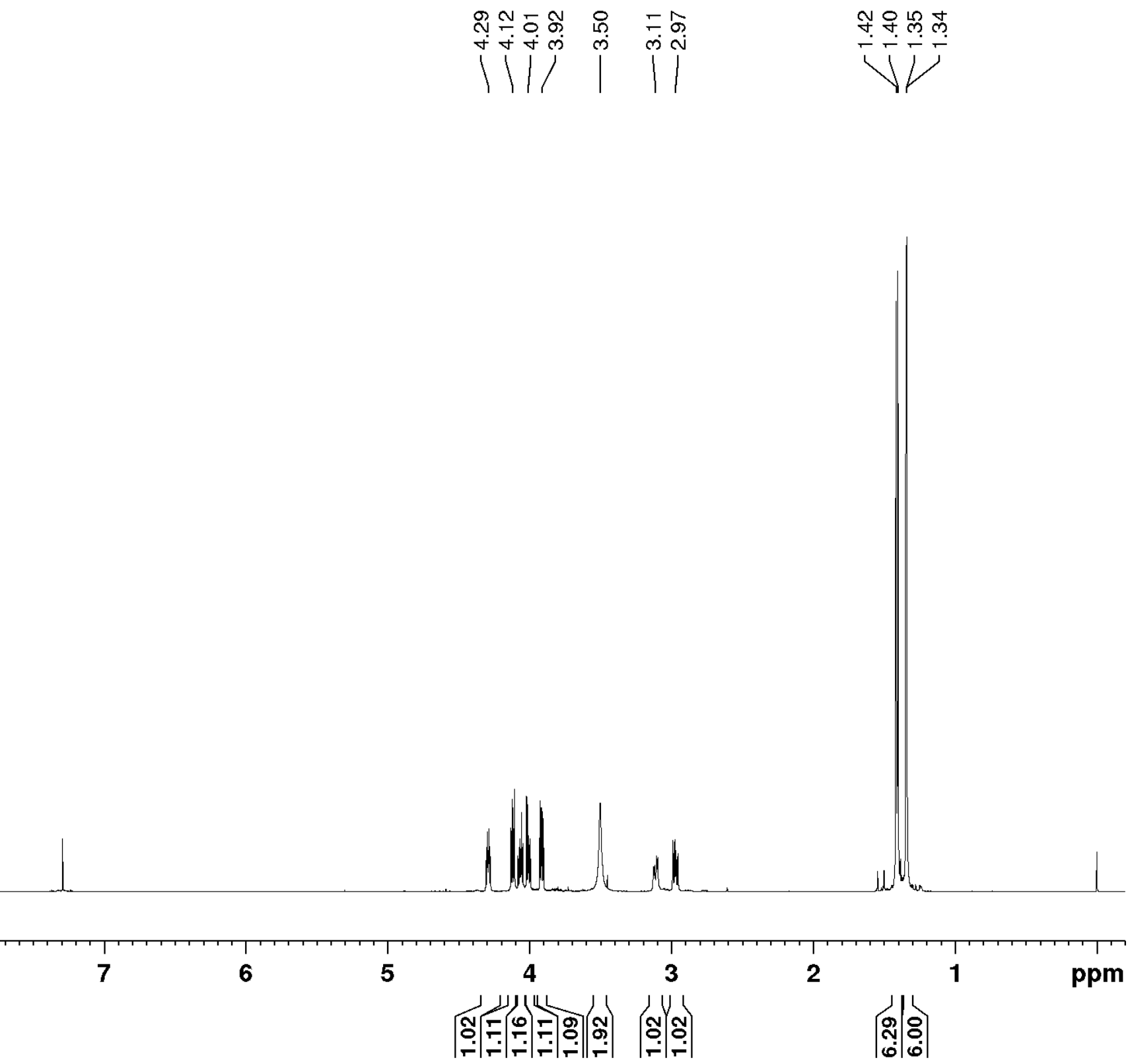

^{13}C NMR spectrum (150 MHz, $CDCl_3$)

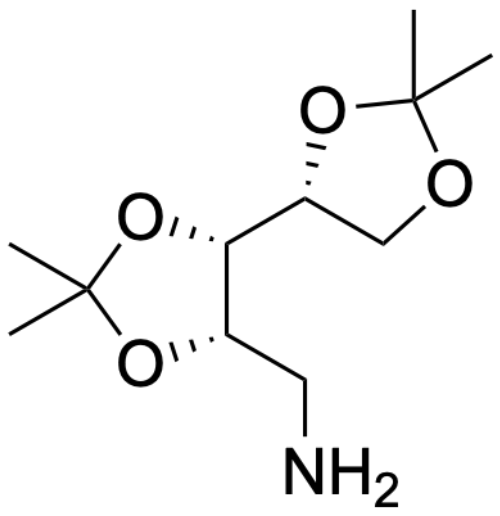

11

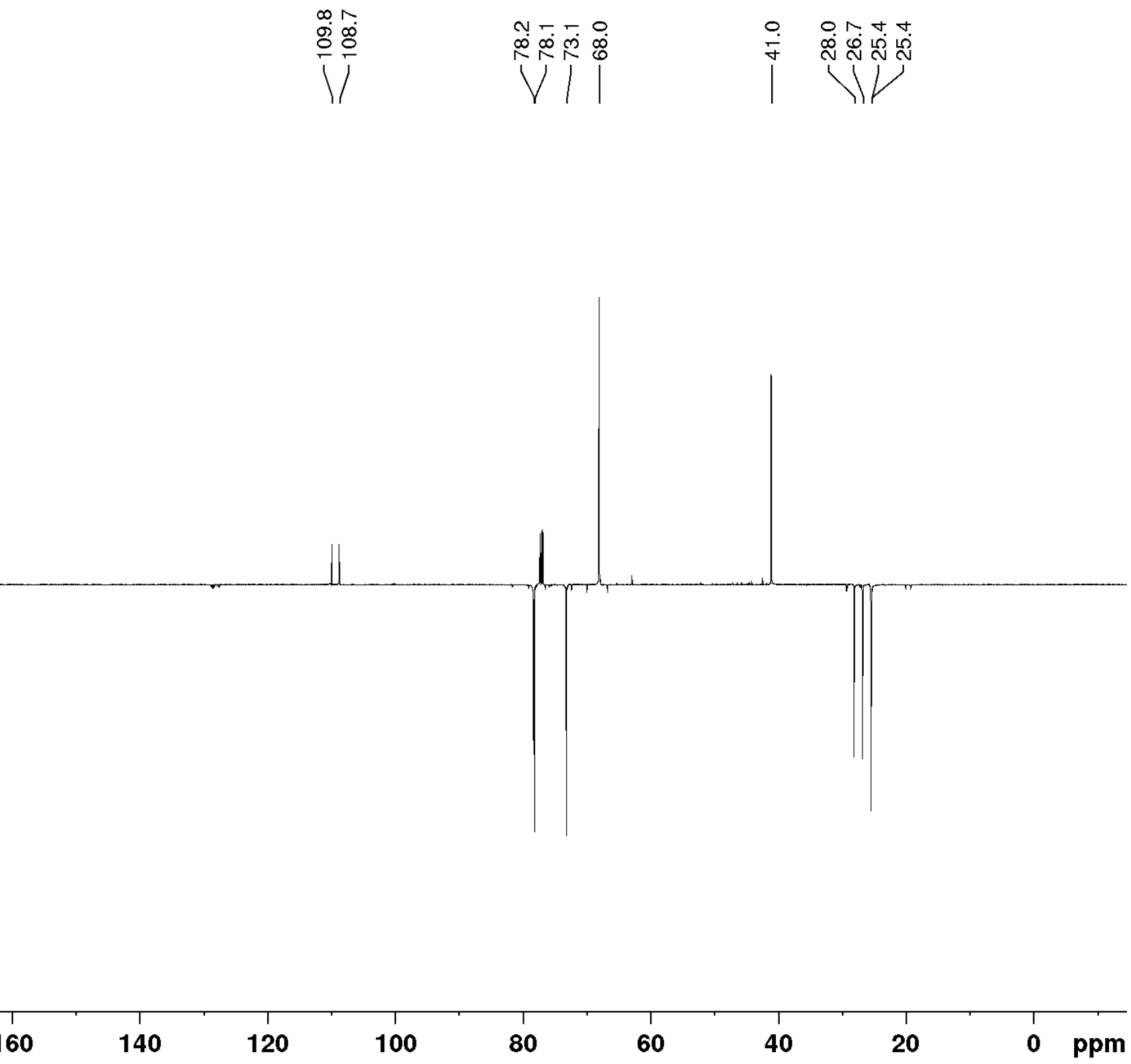

^{1}H NMR spectrum (600 MHz, $CDCl_3$)

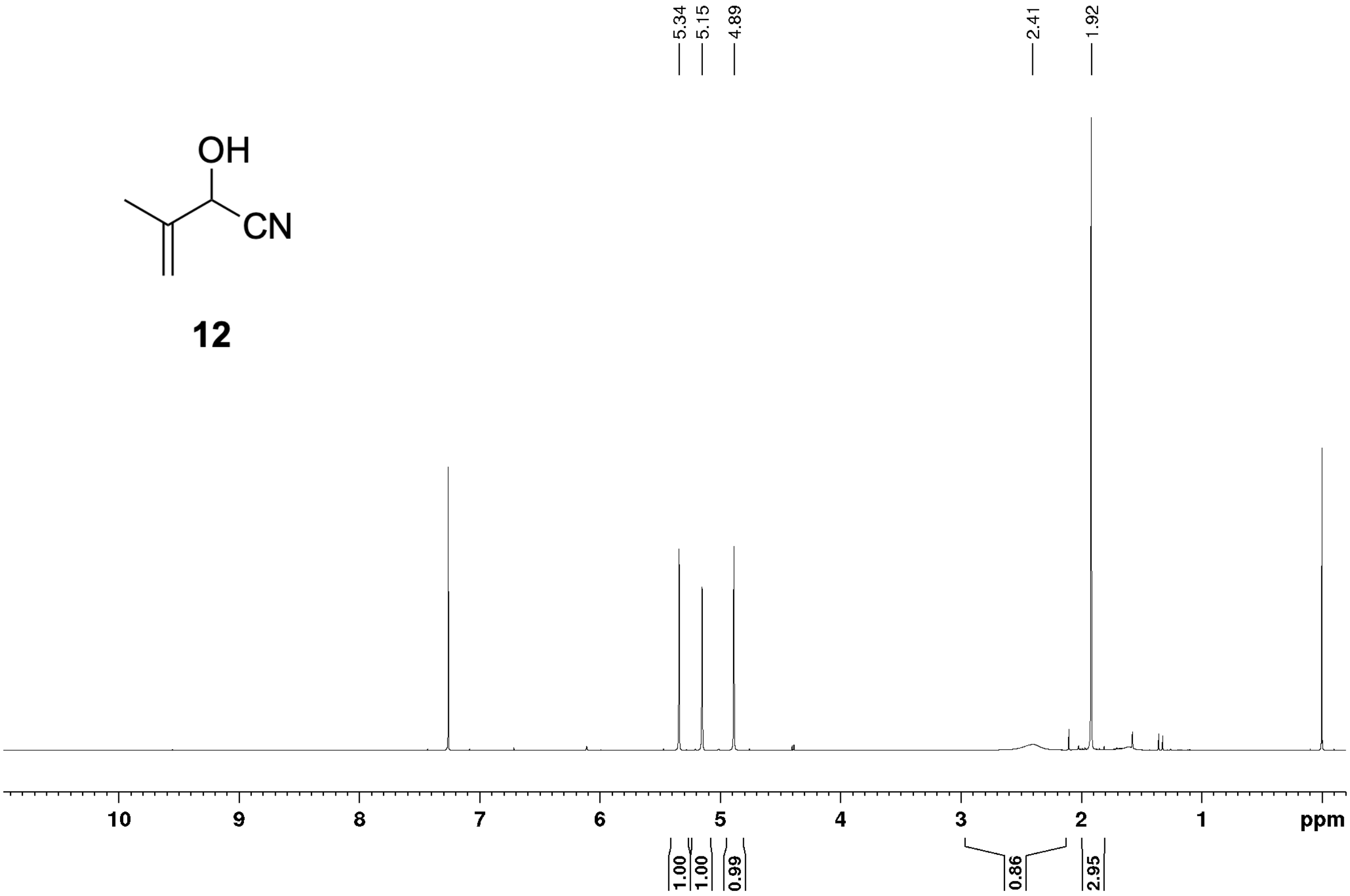

^{13}C NMR spectrum (150 MHz, $CDCl_3$)

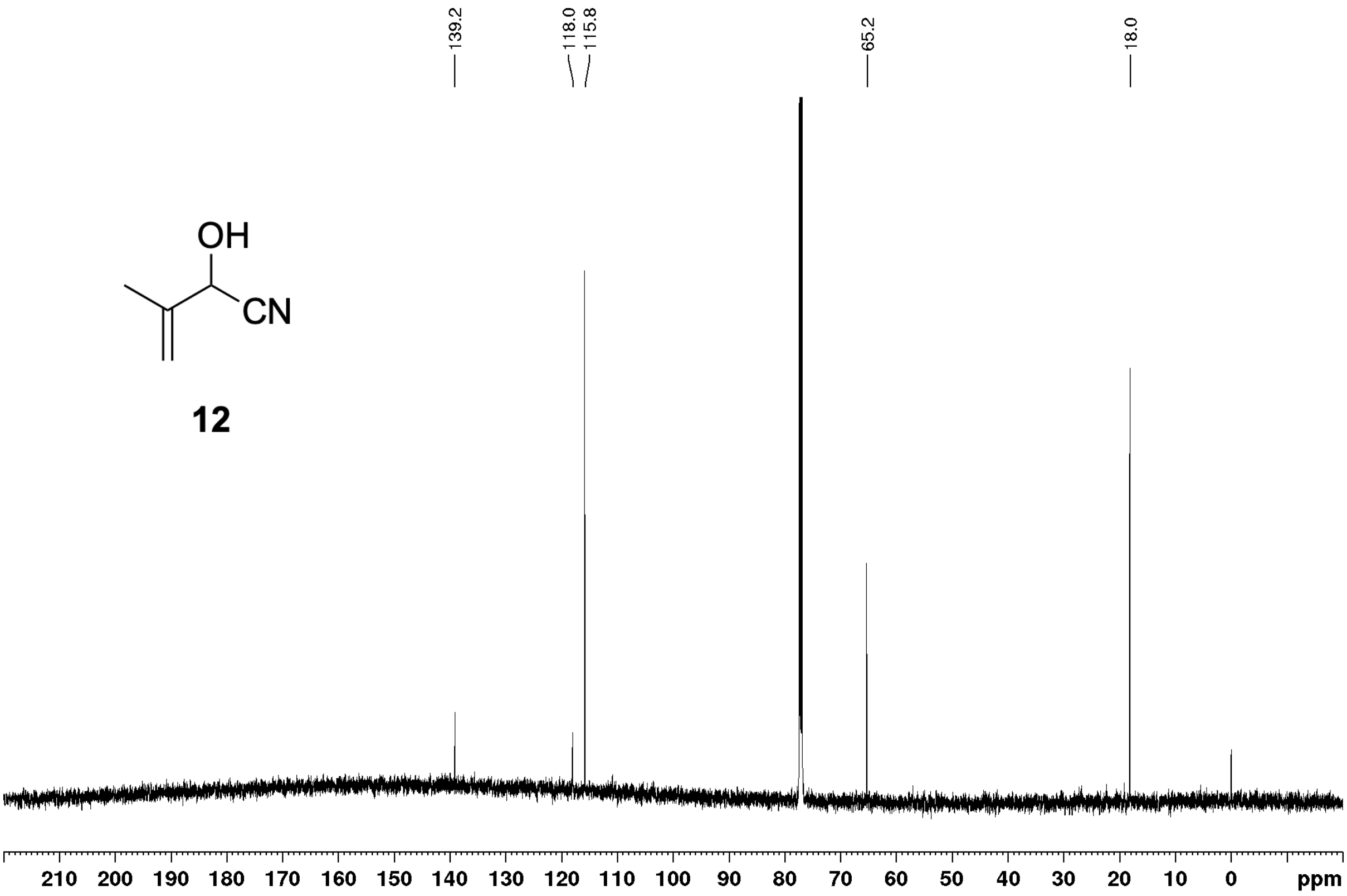

^{1}H NMR spectrum (600 MHz, $CDCl_3$)

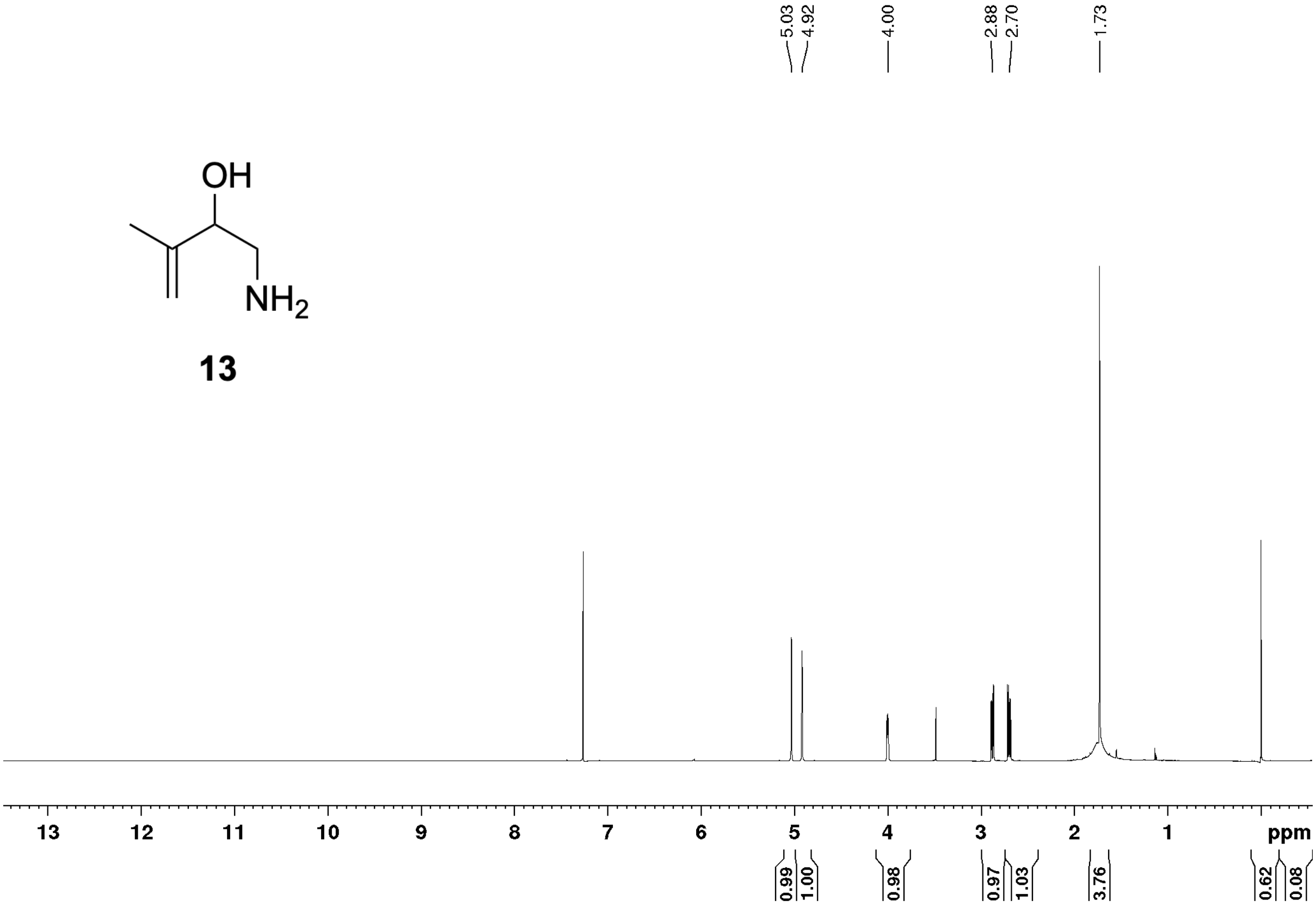

^{13}C NMR spectrum (150 MHz, $CDCl_3$)

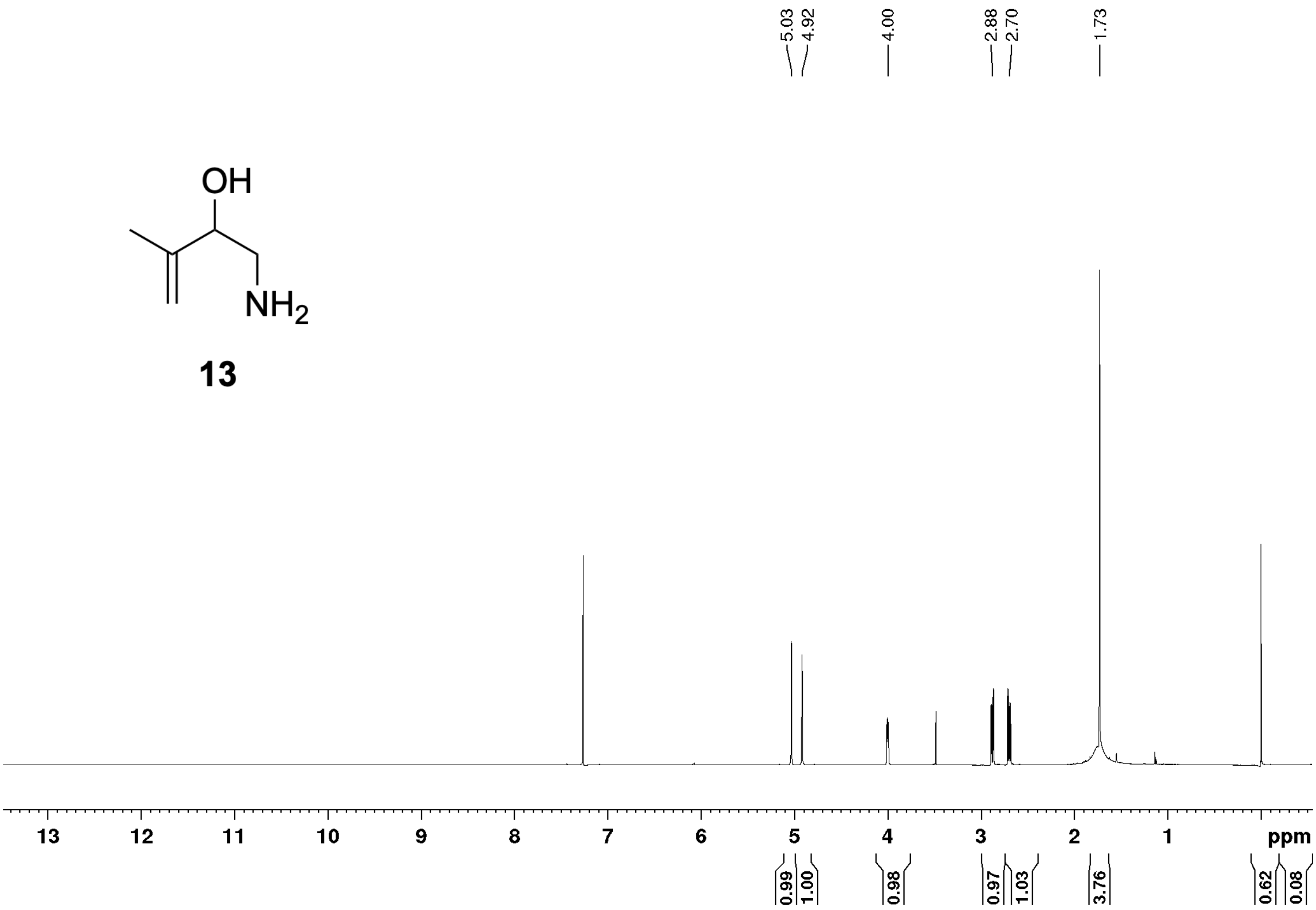

^{1}H NMR spectrum (600 MHz, $CDCl_3$)

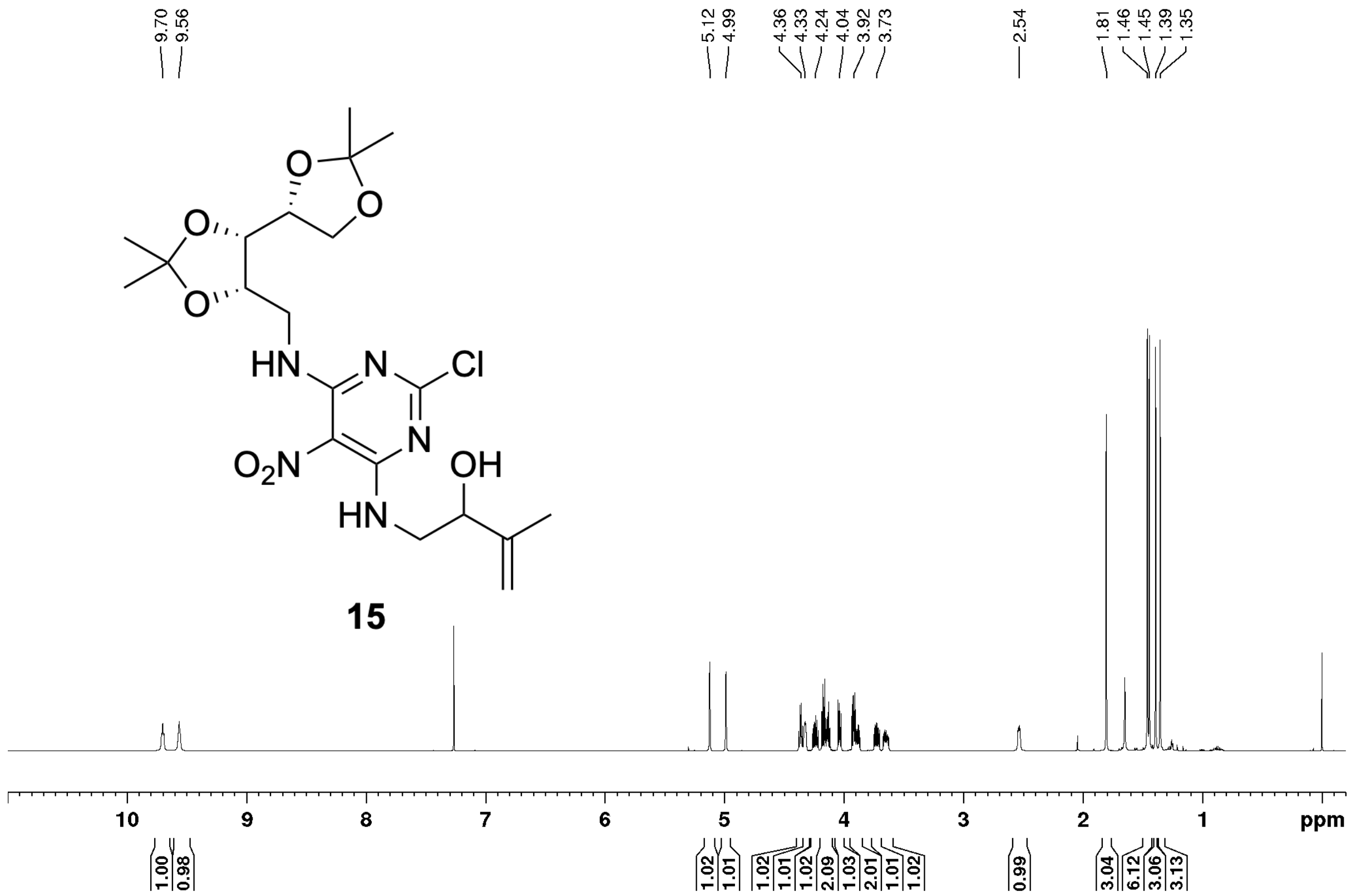

^{13}C NMR spectrum (150 MHz, $CDCl_3$)

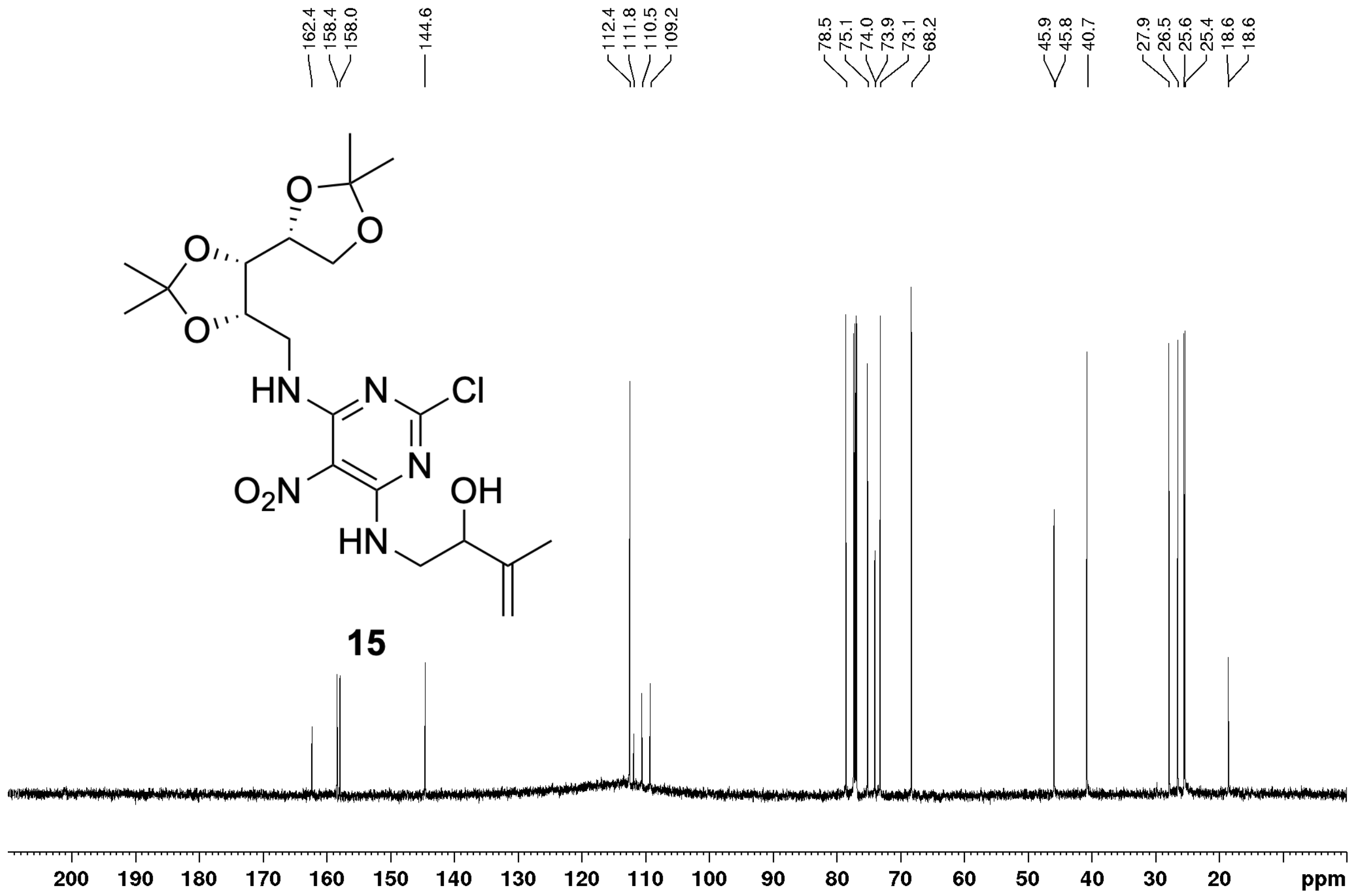

^{1}H NMR spectrum (600 MHz, $CDCl_3$)

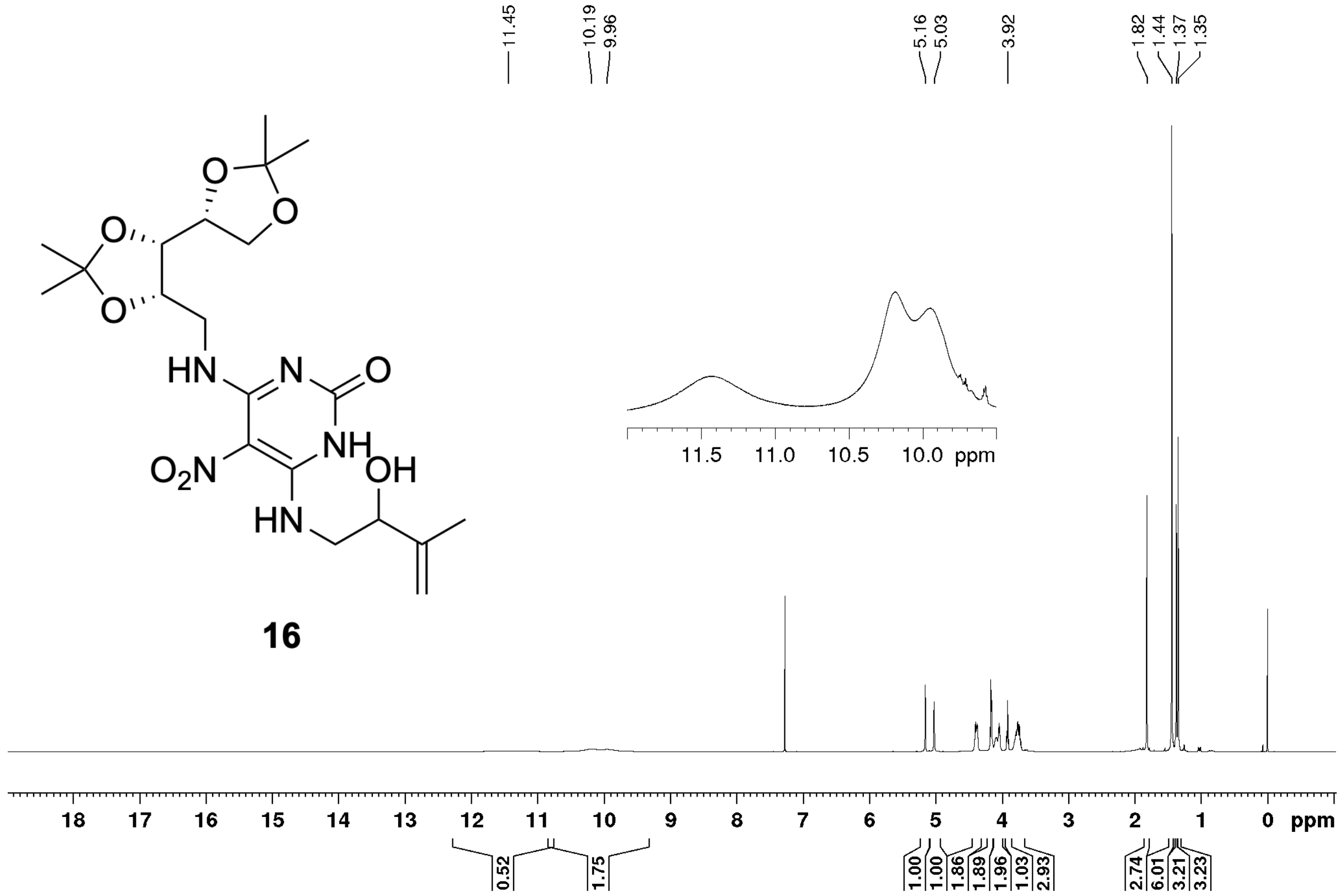

^{13}C NMR spectrum (150 MHz, $CDCl_3$)

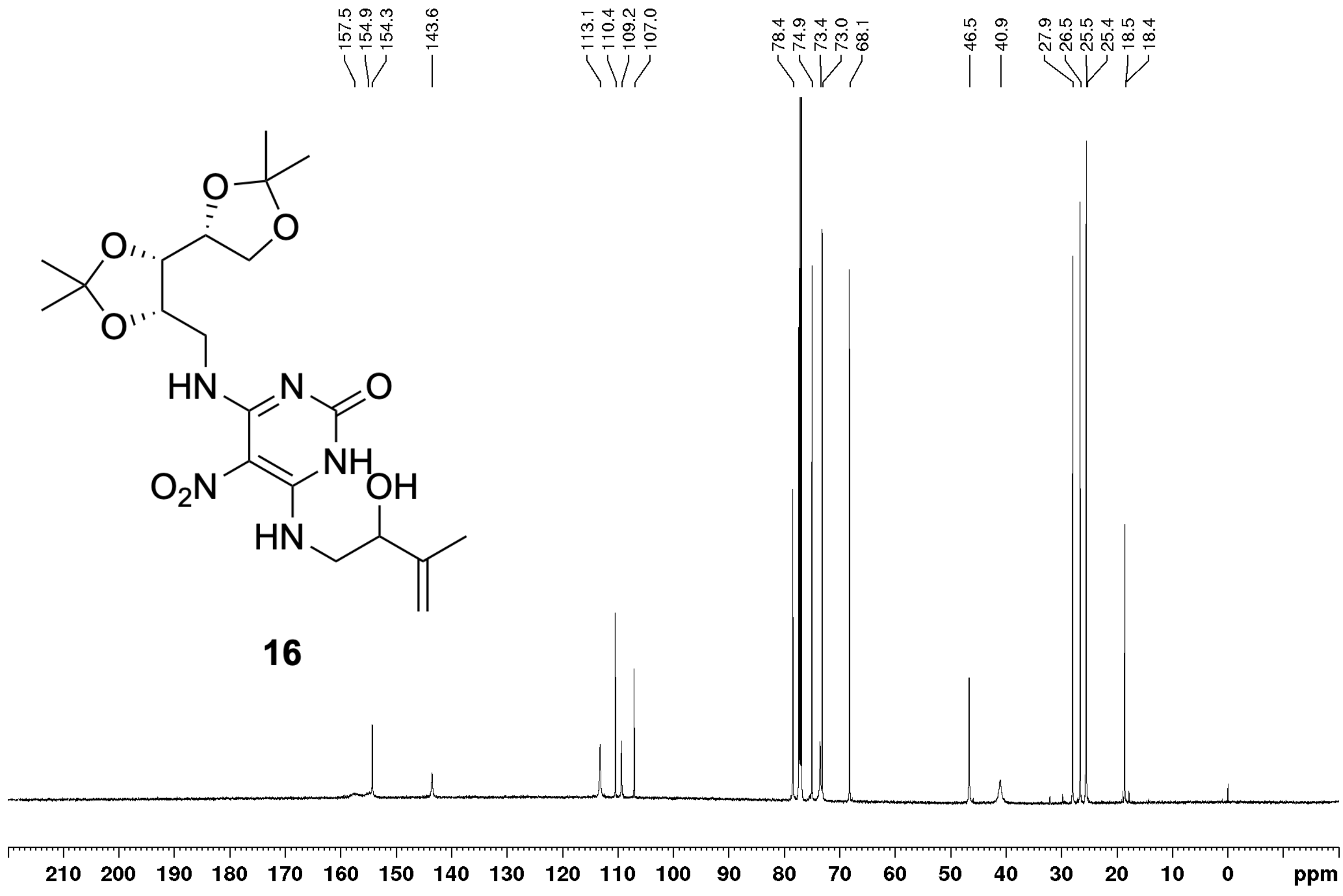

^{1}H NMR spectrum (600 MHz, $CDCl_3$)

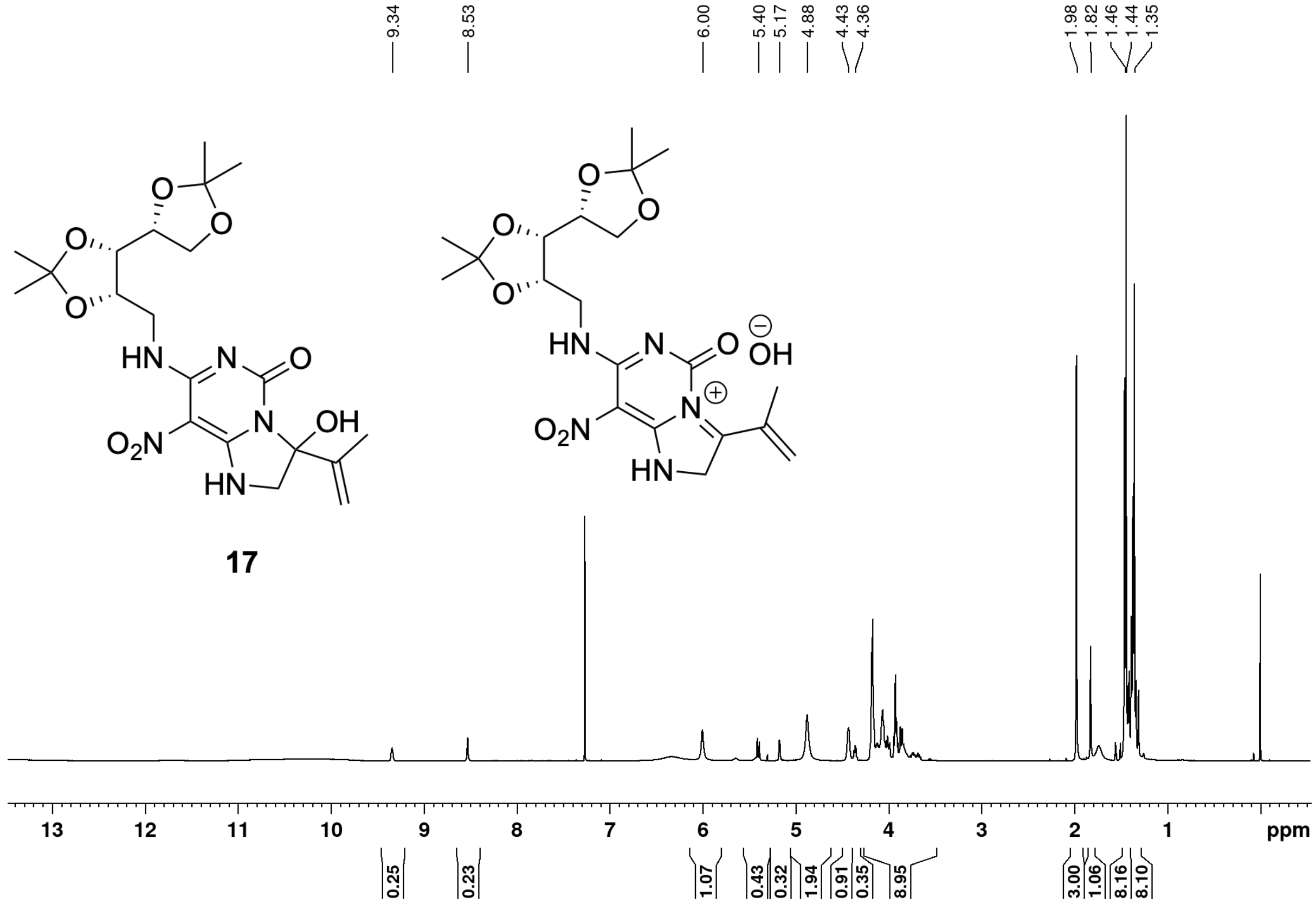

^{13}C NMR spectrum (150 MHz, $CDCl_3$)

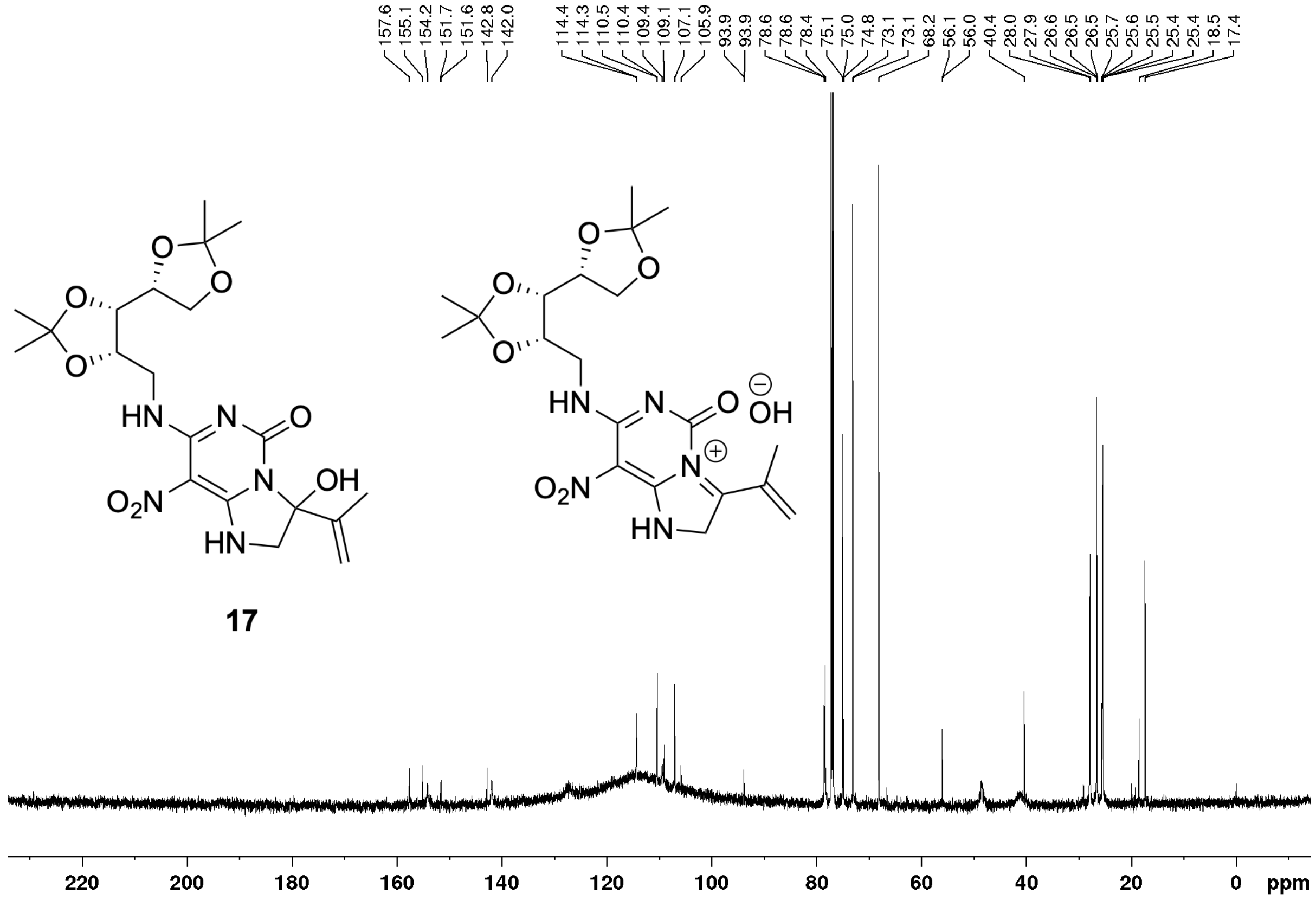

HSQC NMR spectrum ($CDCl_3$)

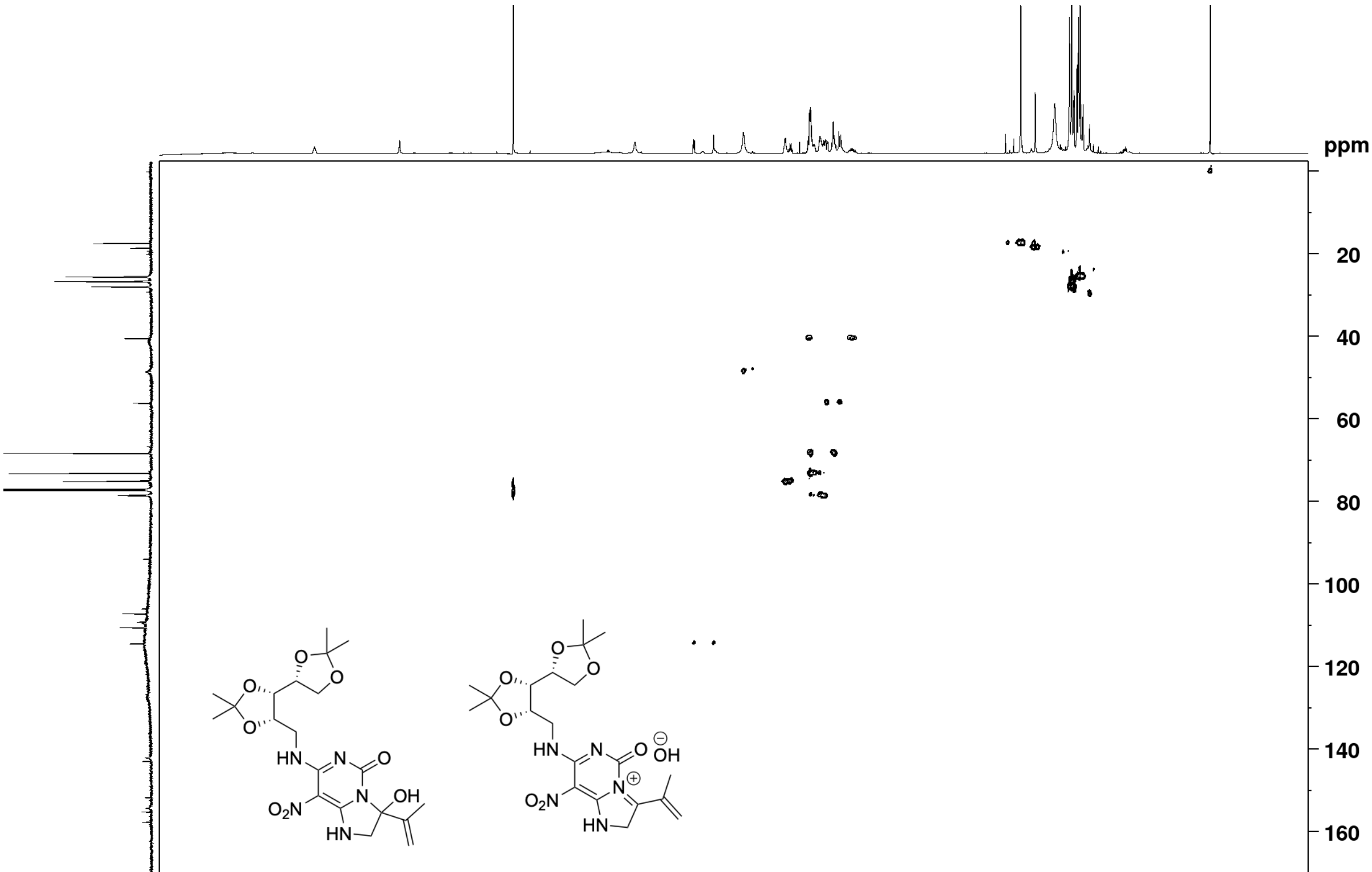

17

^{1}H NMR spectrum (600 MHz, $CDCl_3$)

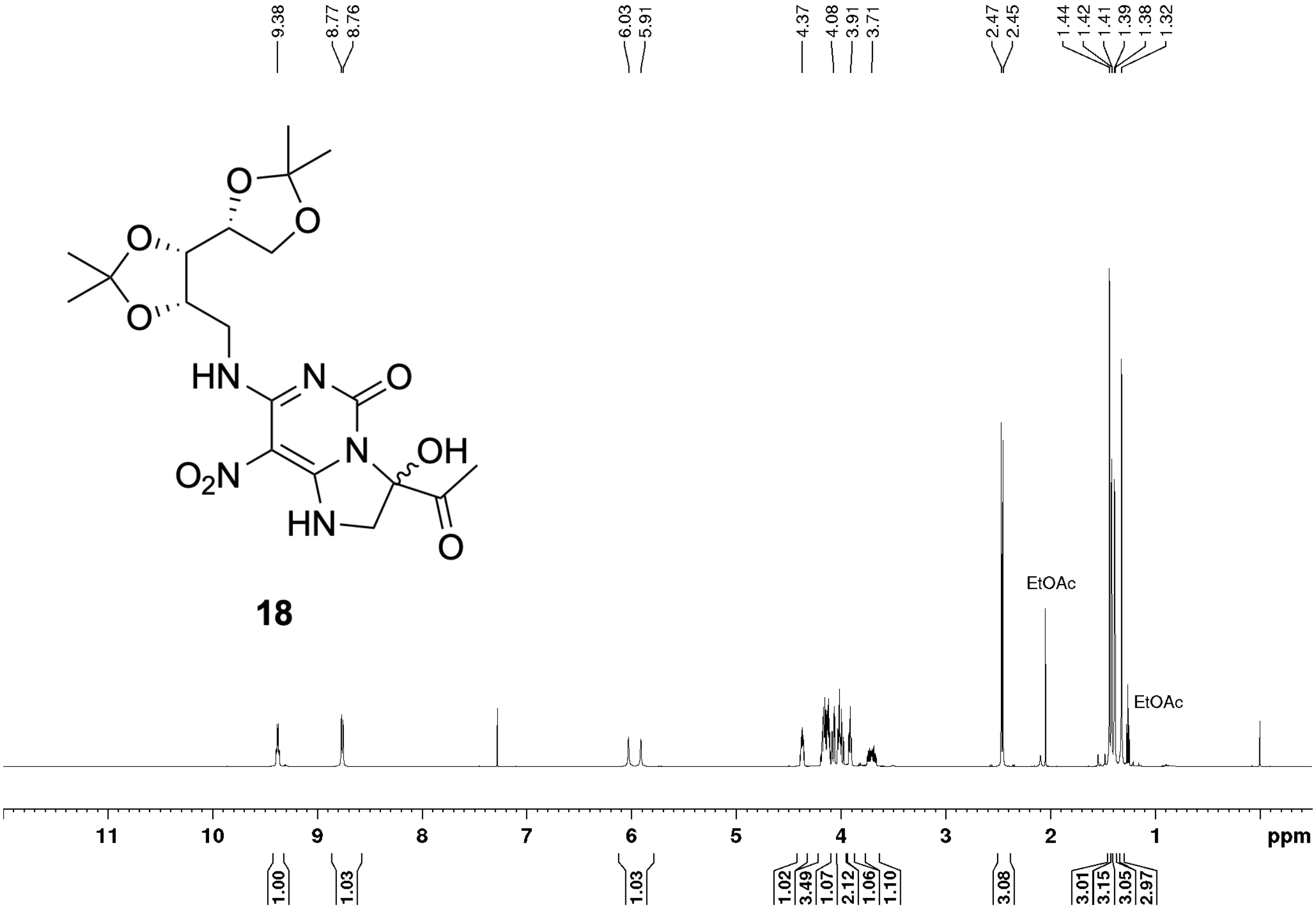

^{13}C NMR spectrum (150 MHz, $CDCl_3$)

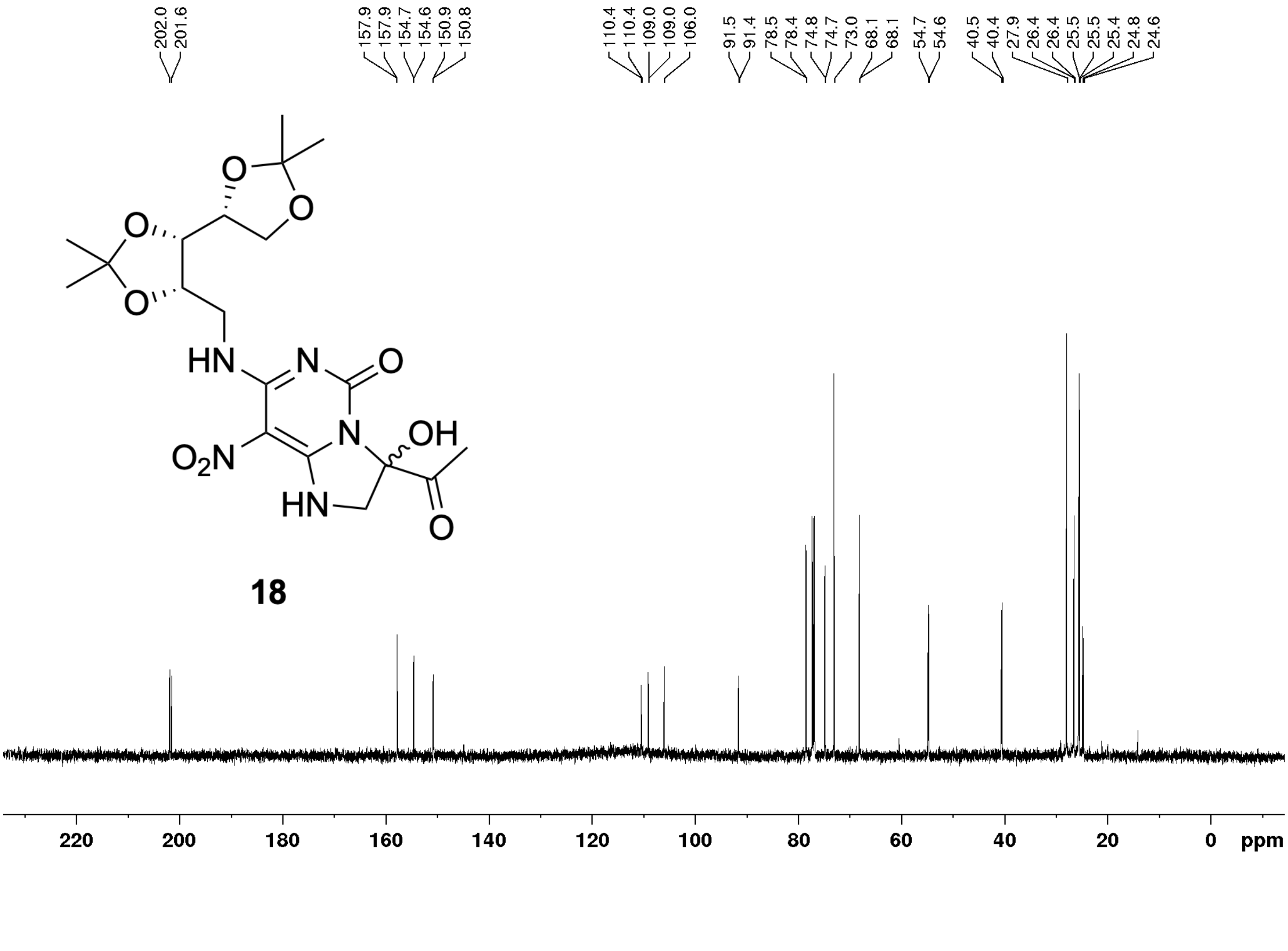

^1H NMR spectrum (600 MHz, $CDCl_3$)

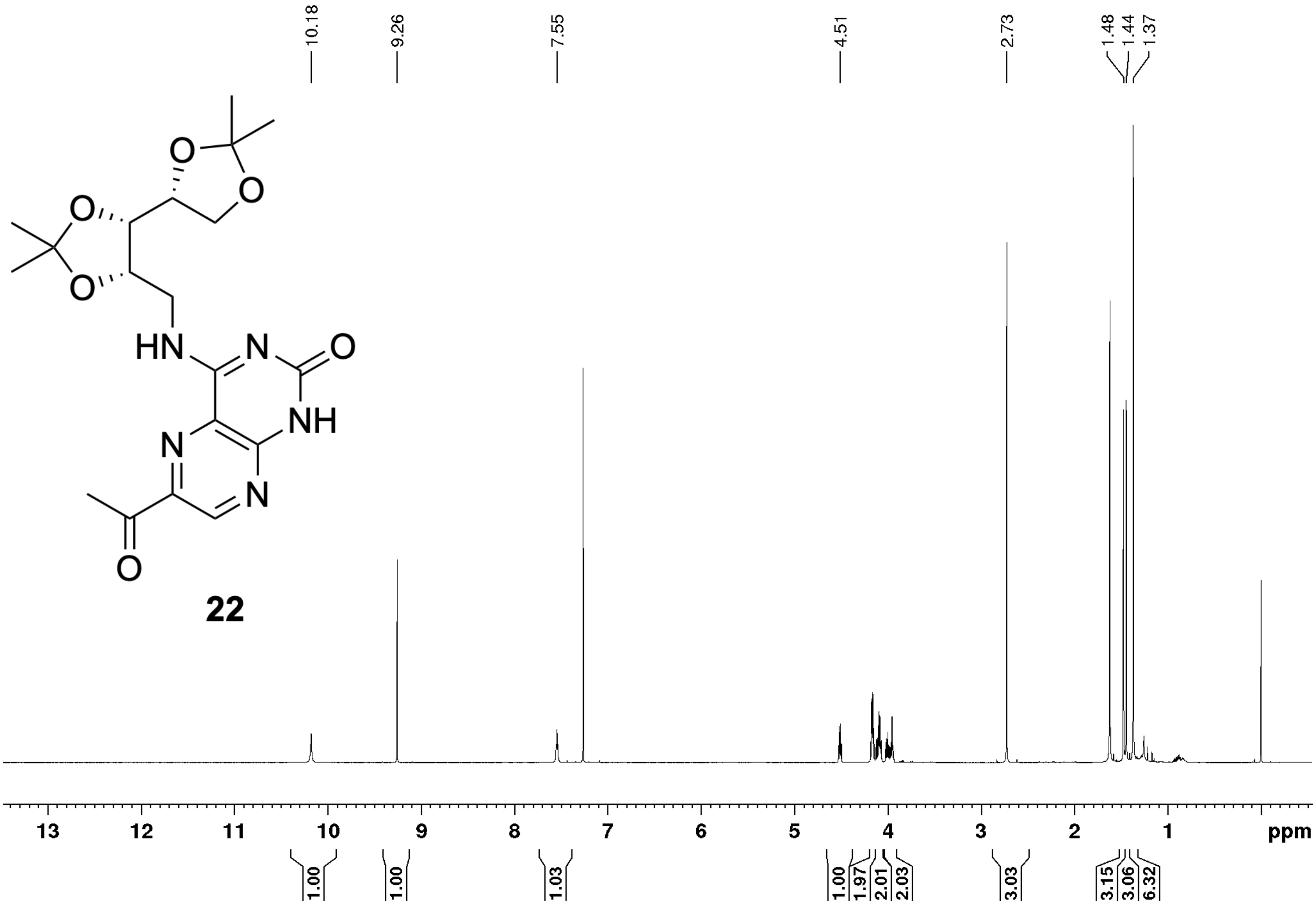

^{13}C NMR spectrum (150 MHz, $CDCl_3$)

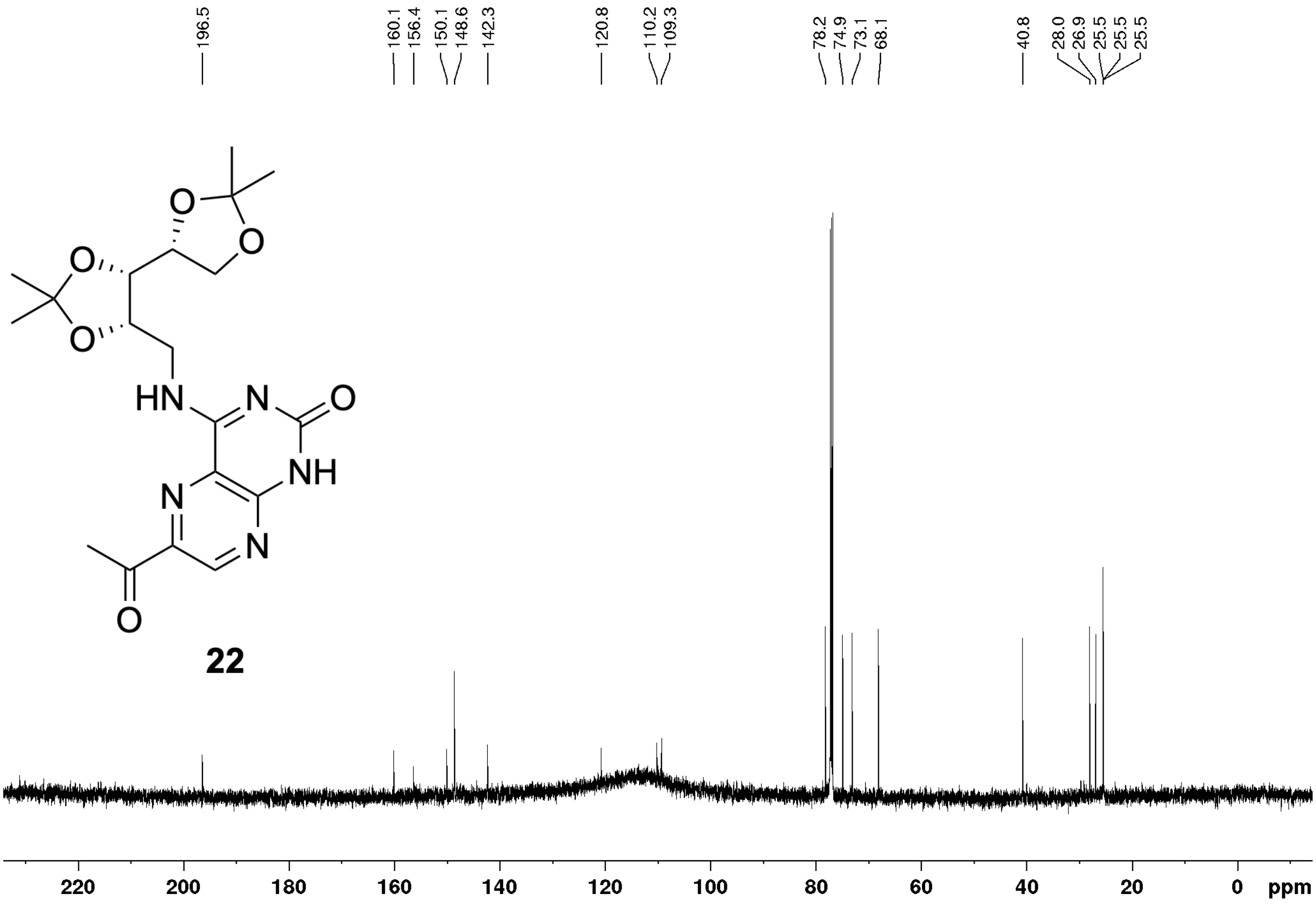

2D COSY NMR spectrum ($CDCl_3$)

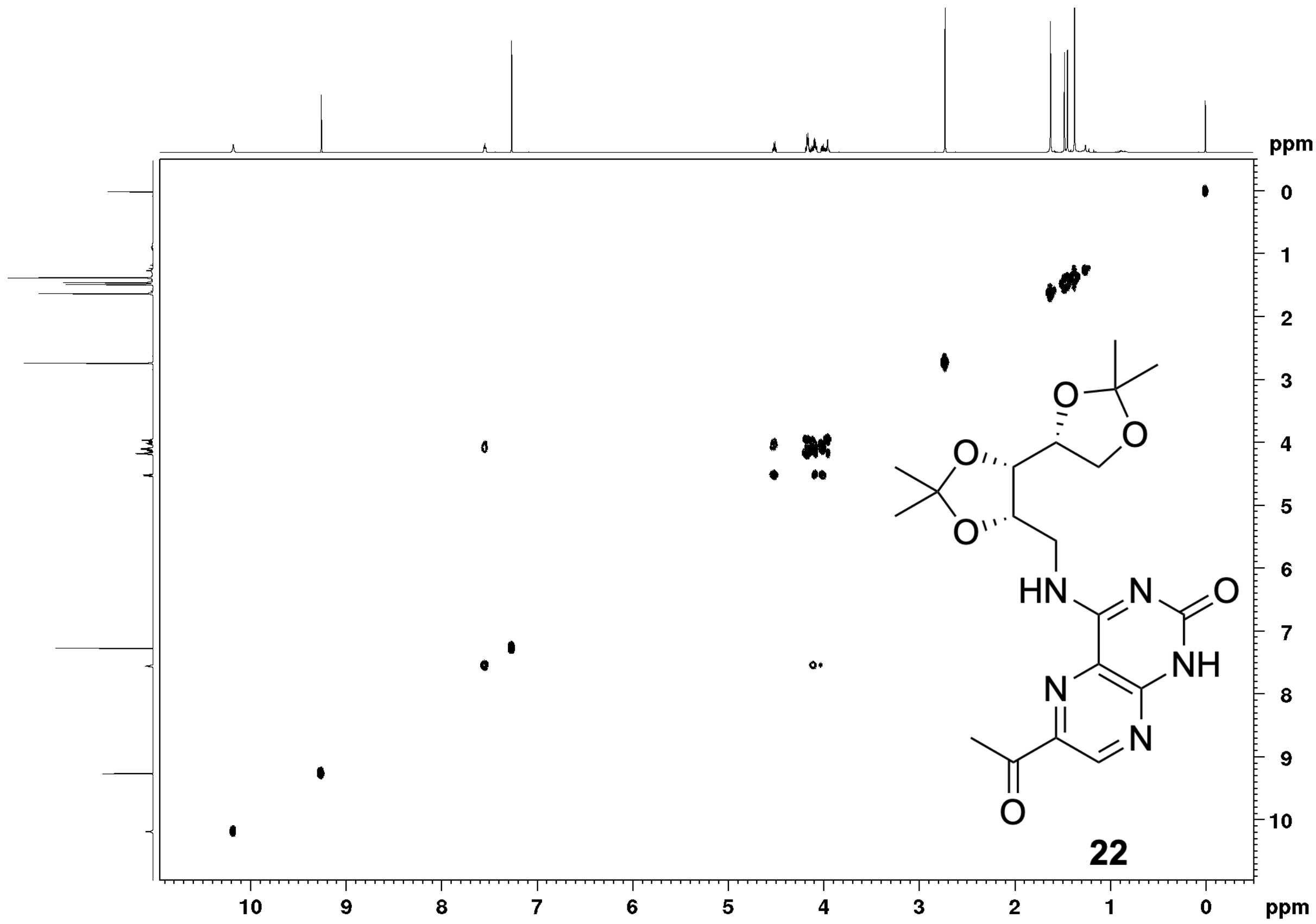

HMBC NMR spectrum ($CDCl_3$)

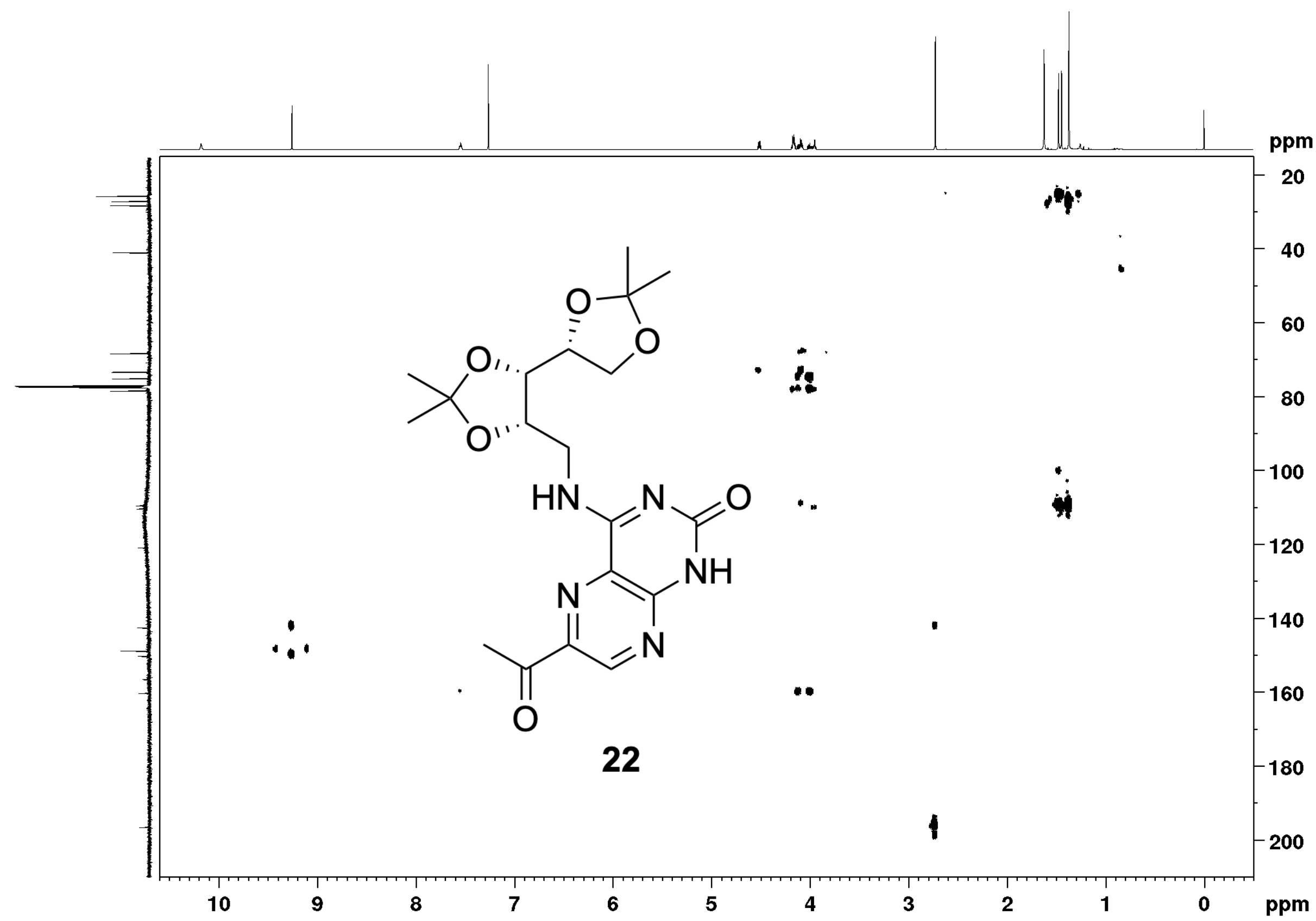

HSQC NMR spectrum ($CDCl_3$)

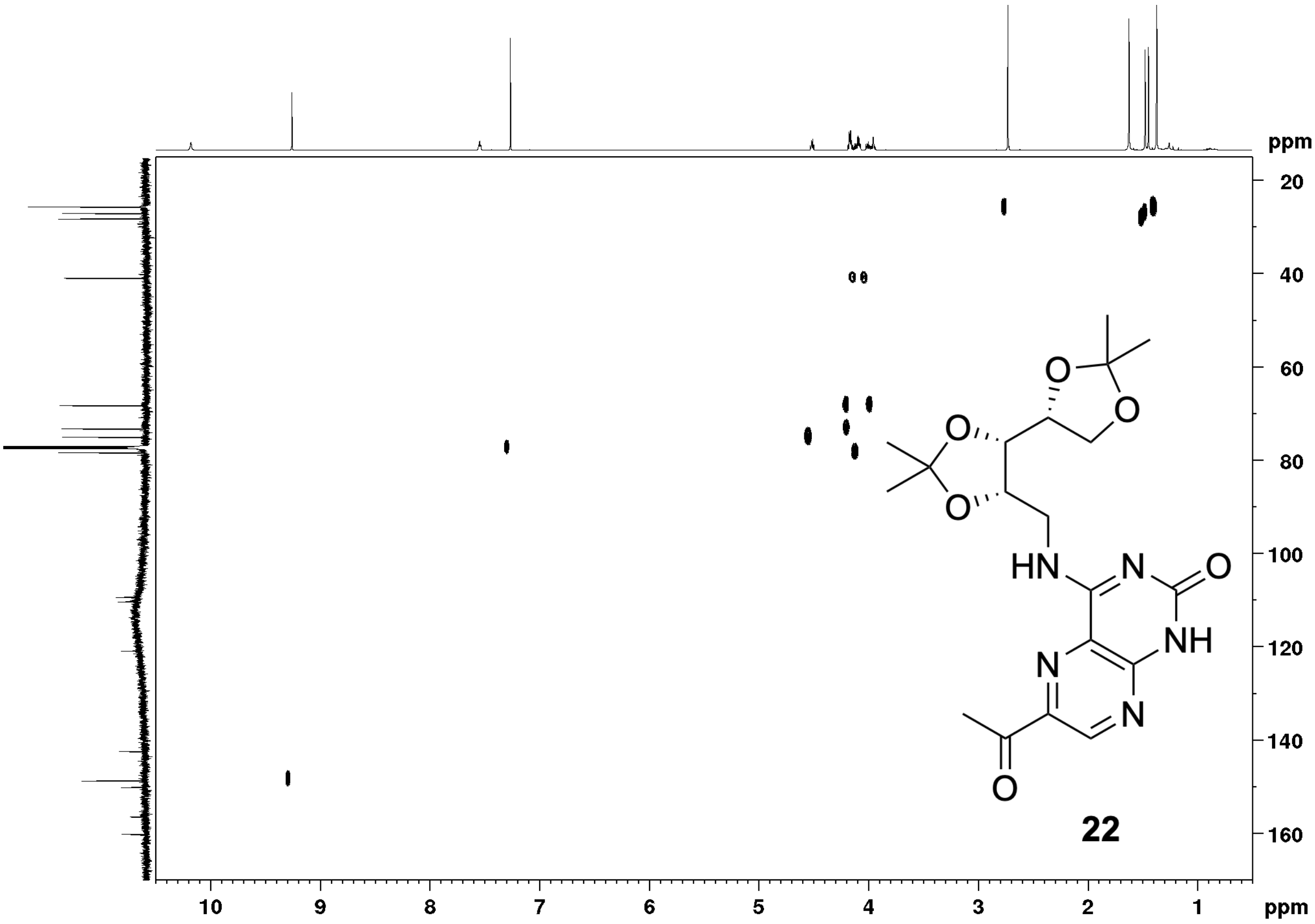

^{1}H NMR spectrum (600 MHz, 10 % D_2O/H_2O)

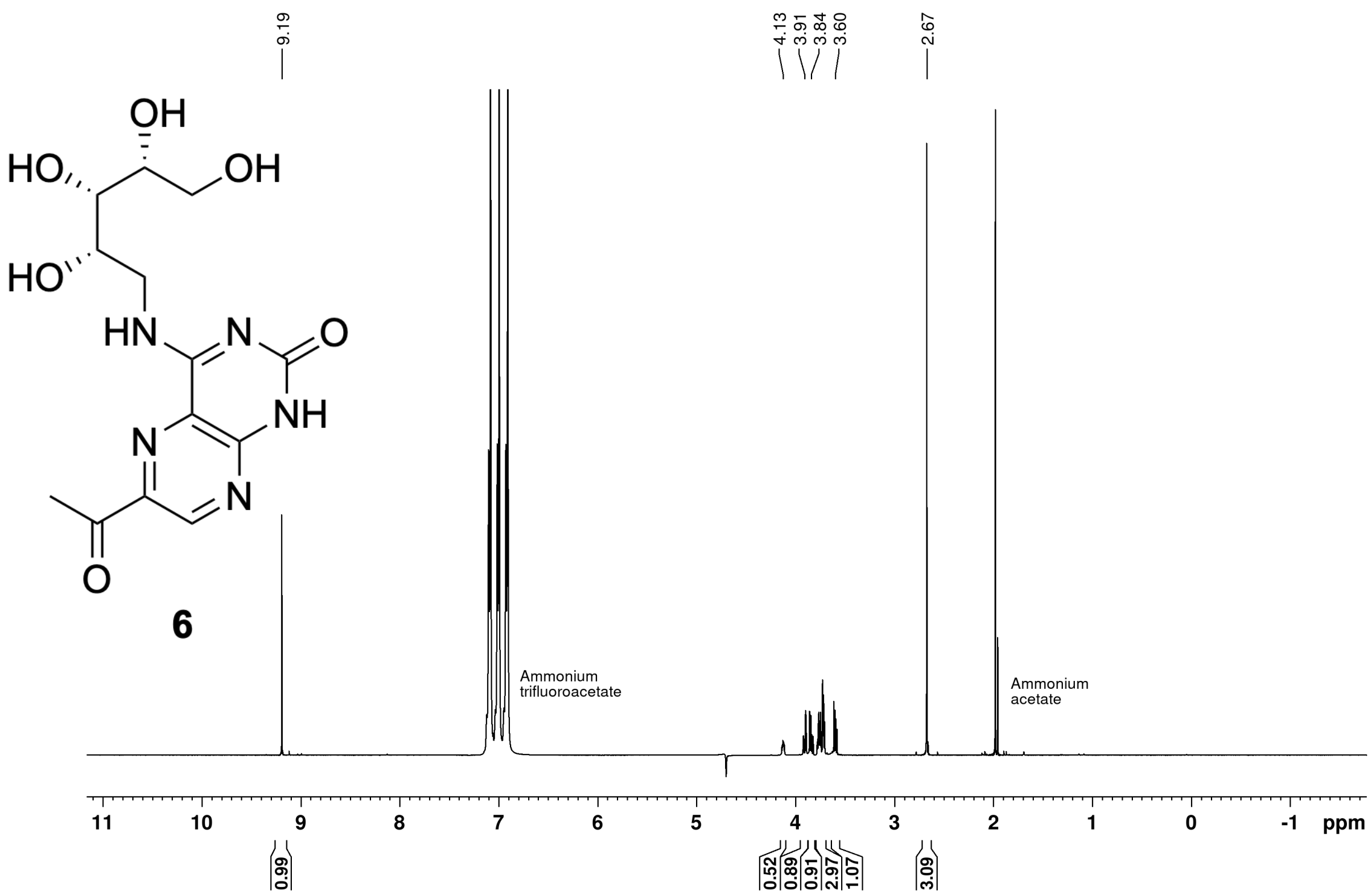

^{13}C NMR spectrum, proton coupled (150 MHz, 10 % D_2O/H_2O)

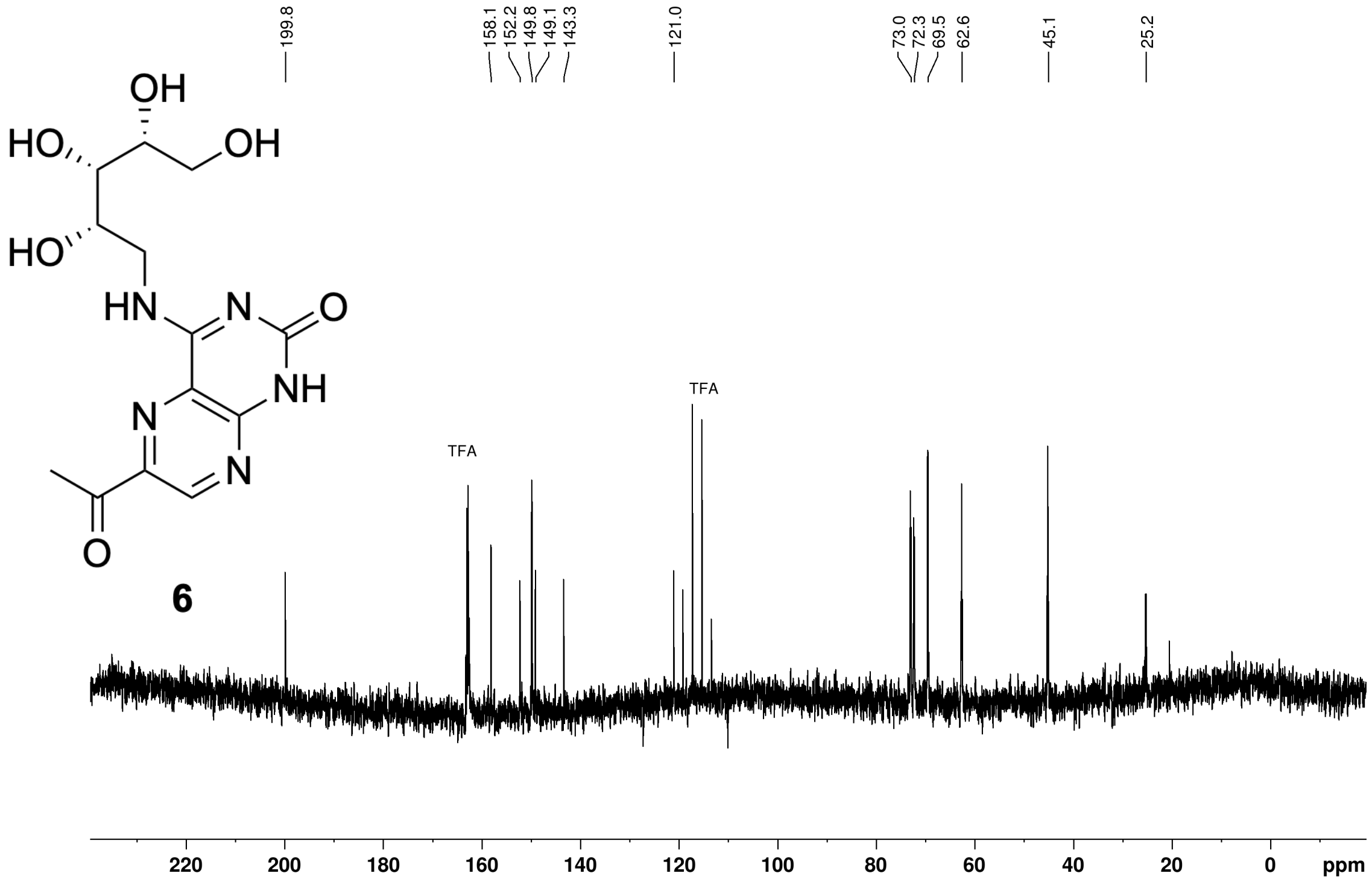

References

1. J. Y. W. Mak, R. J. D. Rivero, H. N. Hoang, X. Y. Lim, J. Deng, H. E. G. McWilliam, J. A. Villadangos, J. McCluskey, A. J. Corbett and D. P. Fairlie, *Angew. Chem. Int. Ed.*, 2024, **63**, e202400632.
2. W. Awad, G. J. M. Ler, W. Xu, A. N. Keller, J. Y. W. Mak, X. Y. Lim, L. Liu, S. B. G. Eckle, J. Le Nours, J. McCluskey, A. J. Corbett, D. P. Fairlie and J. Rossjohn, *Nat. Immunol.*, 2020, **21**, 400-411.
3. C. J. H. Wang, W. Awad, L. Liu, J. Y. W. Mak, N. Veerapen, P. T. Illing, A. W. Purcell, S. B. G. Eckle, J. McCluskey, G. S. Besra, D. P. Fairlie, J. Rossjohn and J. Le Nours, *J. Biol. Chem.*, 2022, **298**, 102714.
4. O. Patel, L. Kjer-Nielsen, J. Le Nours, S. B. Eckle, R. Birkinshaw, T. Beddoe, A. J. Corbett, L. Liu, J. J. Miles, B. Meehan, R. Reantragoon, M. L. Sandoval-Romero, L. C. Sullivan, A. G. Brooks, Z. Chen, D. P. Fairlie, J. McCluskey and J. Rossjohn, *Nat. Commun.*, 2013, **4**, 2142.
5. W. Kabsch, *Acta Crystallogr. D Biol. Crystallogr.*, 2010, **66**, 133-144.
6. P. V. Afonine, R. W. Grosse-Kunstleve, N. Echols, J. J. Headd, N. W. Moriarty, M. Mustyakimov, T. C. Terwilliger, A. Urzhumtsev, P. H. Zwart and P. D. Adams, *Acta Crystallogr. D Biol. Crystallogr.*, 2012, **68**, 352-367.
7. M. D. Winn, C. C. Ballard, K. D. Cowtan, E. J. Dodson, P. Emsley, P. R. Evans, R. M. Keegan, E. B. Krissinel, A. G. Leslie, A. McCoy, S. J. McNicholas, G. N. Murshudov, N. S. Pannu, E. A. Potterton, H. R. Powell, R. J. Read, A. Vagin and K. S. Wilson, *Acta Crystallogr. D Biol. Crystallogr.*, 2011, **67**, 235-242.
8. A. J. McCoy, *Acta Crystallogr. D Biol. Crystallogr.*, 2007, **63**, 32-41.
9. P. Emsley and K. Cowtan, *Acta Crystallogr. D Biol. Crystallogr.*, 2004, **60**, 2126-2132.
10. V. B. Chen, W. B. Arendall, 3rd, J. J. Headd, D. A. Keedy, R. M. Immormino, G. J. Kapral, L. W. Murray, J. S. Richardson and D. C. Richardson, *Acta Crystallogr. D Biol. Crystallogr.*, 2010, **66**, 12-21.
11. A. J. Corbett, S. B. G. Eckle, R. W. Birkinshaw, L. Liu, O. Patel, J. Mahony, Z. Chen, R. Reantragoon, B. Meehan, H. Cao, N. A. Williamson, R. A. Strugnell, D. Van Sinderen, J. Y. W. Mak, D. P. Fairlie, L. Kjer-Nielsen, J. Rossjohn and J. McCluskey, *Nature*, 2014, **509**, 361-365.
12. L. Kjer-Nielsen, O. Patel, A. J. Corbett, J. Le Nours, B. Meehan, L. Liu, M. Bhati, Z. Chen, L. Kostenko, R. Reantragoon, N. A. Williamson, A. W. Purcell, N. L. Dudek, M. J. McConville, R. A. J. O'Hair, G. N. Khairallah, D. I. Godfrey, D. P. Fairlie, J. Rossjohn and J. McCluskey, *Nature*, 2012, **491**, 717-723.
13. S. Rosskopf, J. Leitner, W. Paster, L. T. Morton, R. S. Hagedoorn, P. Steinberger and M. H. M. Heemskerk, *Oncotarget*, 2018, **9**, 17608-17619.
14. J. Davoll and D. D. Evans, *J. Chem. Soc.*, 1960, DOI: 10.1039/jr9600005041, 5041-5049.
15. V. Sharma, G. T. Kelly and C. M. H. Watanabe, *Org. Lett.*, 2008, **10**, 4815-4818.
16. G. Wider and L. Dreier, *J. Am. Chem. Soc.*, 2006, **128**, 2571-2576.
17. J. Y. W. Mak, *Aust. J. Chem.*, 2022, **75**, 160-164.
